# Neurochondrin promotes U5 snRNP maturation by regulating AAR2 release from PRPF8

**DOI:** 10.1093/nar/gkag685

**Published:** 2026-07-07

**Authors:** Tingrong Ren, Wanru Huang, Gaigai Wei, Haiping Zhao, Yuqi Zhang, Jingjing Yi, Zhihan Guo, Yihan Wang, Jiating Kuang, Zhaoying Sheng, Huiling Zhang, Duanwu Zhang

**Affiliations:** Fudan University Pudong Medical Center and Shanghai Key Laboratory of Medical Epigenetics, International Co-laboratory of Medical Epigenetics and Metabolism, Ministry of Science and Technology, Institutes of Biomedical Sciences, Fudan University, Shanghai 200032, China; Fudan University Pudong Medical Center and Shanghai Key Laboratory of Medical Epigenetics, International Co-laboratory of Medical Epigenetics and Metabolism, Ministry of Science and Technology, Institutes of Biomedical Sciences, Fudan University, Shanghai 200032, China; Fudan University Pudong Medical Center and Shanghai Key Laboratory of Medical Epigenetics, International Co-laboratory of Medical Epigenetics and Metabolism, Ministry of Science and Technology, Institutes of Biomedical Sciences, Fudan University, Shanghai 200032, China; Fudan University Pudong Medical Center and Shanghai Key Laboratory of Medical Epigenetics, International Co-laboratory of Medical Epigenetics and Metabolism, Ministry of Science and Technology, Institutes of Biomedical Sciences, Fudan University, Shanghai 200032, China; Fudan University Pudong Medical Center and Shanghai Key Laboratory of Medical Epigenetics, International Co-laboratory of Medical Epigenetics and Metabolism, Ministry of Science and Technology, Institutes of Biomedical Sciences, Fudan University, Shanghai 200032, China; Fudan University Pudong Medical Center and Shanghai Key Laboratory of Medical Epigenetics, International Co-laboratory of Medical Epigenetics and Metabolism, Ministry of Science and Technology, Institutes of Biomedical Sciences, Fudan University, Shanghai 200032, China; Fudan University Pudong Medical Center and Shanghai Key Laboratory of Medical Epigenetics, International Co-laboratory of Medical Epigenetics and Metabolism, Ministry of Science and Technology, Institutes of Biomedical Sciences, Fudan University, Shanghai 200032, China; Fudan University Pudong Medical Center and Shanghai Key Laboratory of Medical Epigenetics, International Co-laboratory of Medical Epigenetics and Metabolism, Ministry of Science and Technology, Institutes of Biomedical Sciences, Fudan University, Shanghai 200032, China; Fudan University Pudong Medical Center and Shanghai Key Laboratory of Medical Epigenetics, International Co-laboratory of Medical Epigenetics and Metabolism, Ministry of Science and Technology, Institutes of Biomedical Sciences, Fudan University, Shanghai 200032, China; Fudan University Pudong Medical Center and Shanghai Key Laboratory of Medical Epigenetics, International Co-laboratory of Medical Epigenetics and Metabolism, Ministry of Science and Technology, Institutes of Biomedical Sciences, Fudan University, Shanghai 200032, China; Fudan University Pudong Medical Center and Shanghai Key Laboratory of Medical Epigenetics, International Co-laboratory of Medical Epigenetics and Metabolism, Ministry of Science and Technology, Institutes of Biomedical Sciences, Fudan University, Shanghai 200032, China; Fudan University Pudong Medical Center and Shanghai Key Laboratory of Medical Epigenetics, International Co-laboratory of Medical Epigenetics and Metabolism, Ministry of Science and Technology, Institutes of Biomedical Sciences, Fudan University, Shanghai 200032, China

## Abstract

Pre-mRNA splicing is orchestrated by the spliceosome, a dynamic and highly regulated ribonucleoprotein complex composed of five small nuclear ribonucleoproteins (snRNPs). Despite extensive studies, the biogenesis of snRNPs remains incompletely understood. Here, we identify neurochondrin (NCDN) as a critical regulator of U5 snRNP biogenesis. NCDN associates with PRPF8–AAR2–EFTUD2 complex in the cytoplasm and is essential for the proper progression of this assembly intermediate toward mature U5 snRNP formation. Loss of NCDN causes the accumulation of this intermediate, resulting in a decreased level of mature U5 snRNP. Spliceosome dysregulation often leads to alternative splicing abnormalities implicated in cancer. Indeed, NCDN deficiency suppresses tumor cell proliferation and induces apoptosis, while high NCDN expression promotes tumor cell growth and correlates with poor survival in glioblastoma patients. Transcriptome analyses reveal that loss of NCDN causes widespread alternative splicing defects and changes in gene expression. Collectively, these results establish NCDN as an essential factor for U5 snRNP assembly and spliceosome function, and highlight its potential as a therapeutic target in glioma with elevated NCDN expression.

## Graphical Abstract

**Graphical Abstract:**
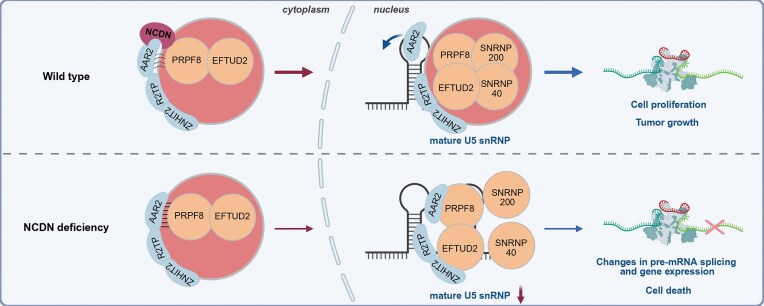

## Introduction

Pre-mRNA splicing is a fundamental step in eukaryotic gene expression, ensuring the precise removal of introns and the ligation of exons to generate mature messenger RNA (mRNA) [1, 2]. Alternative splicing enables a single gene to generate multiple mRNA isoforms through the selection of different exons and splice sites, thereby expanding transcriptomic and proteomic diversity without increasing gene number [3]. This process is catalyzed by the spliceosome, a highly dynamic and precise ribonucleoprotein complex composed of five small nuclear ribonucleoproteins (snRNPs)—U1, U2, U4, U5, and U6—along with numerous auxiliary factors [4, 5].

SnRNP assembly is a sequential and compartment-specific process. For example, the U5 snRNP core particle—comprising U5 small nuclear RNA (snRNA), a heptameric Sm-protein ring, and eight U5-specific proteins—is essential for the catalytic steps of splicing. Assembly of the Sm-ring on U5 snRNA is orchestrated in the cytoplasm by the SMN and PRMT5 complexes [6]. Once the core U5 snRNP (U5 snRNA with seven Sm proteins) is assembled, it is imported into the nucleus, where it sequentially recruits additional U5‐specific factors to complete maturation [7, 8]. Although the SMN complex’s role in Sm-core assembly has been intensively studied, the mechanisms governing the biogenesis of U5-specific proteins remain largely elusive.

In yeast, Prp8 associates with the chaperone Aar2 to form a cytoplasmic Prp8–Aar2–Snu114 complex [9–12]. This complex then binds U5 snRNA and translocates into the nucleus, where Aar2 is released and replaced by the helicase Brr2, completing particle maturation [9, 13]. A similar PRPF8–AAR2–EFTUD2 assembly intermediate has been described in human cells [14]. Recent studies have shown that the biogenesis and assembly of U5 snRNP require the intervention of HSP90 and the R2TP complex [14]. Two additional factors—Zinc finger HIT domain-containing protein 2 (ZNHIT2) [15, 16], which bridges U5-specific proteins to the R2TP complex and ECD (Ecdysoneless) [17, 18], which facilitates early steps of U5 snRNP maturation by promoting the association of Prp8 with the core U5 snRNP and maintaining its stability—have been characterized. Furthermore, CD2BP2 and TSSC4 have also been characterized as U5 snRNP and U4/U6·U5 tri-snRNP assembly factors [19–22]. However, the full spectrum of proteins involved in U5 snRNP assembly remains incompletely understood.

Aberrations in spliceosome function are increasingly recognized as contributors to tumorigenesis. Mutations or dysregulation of spliceosomal components can induce widespread splicing alterations, particularly in transcripts encoding cell cycle regulators and apoptotic factors [23–25]. In particular, several cancers exhibit a dependency on specific splicing programs, a phenomenon termed “splicing addiction,” wherein tumor cells rely on these programs for survival and proliferation [26, 27]. This dependency creates a therapeutic window whereby perturbation of spliceosome assembly selectively impairs tumor cell survival while sparing normal tissues [28–30].

Neurochondrin (NCDN) was originally characterized as a neuronal protein involved in neurite outgrowth and synaptic plasticity [31–33]. Although NCDN has not been directly implicated in RNA metabolism or spliceosome biogenesis, its interaction with SMN—a key factor in snRNP assembly—has been reported [34], and recent proteomic screens have further revealed robust interactions between NCDN and core spliceosomal proteins [14, 16, 20]. However, whether and how NCDN contributes to snRNP biogenesis remains unknown.

Here, we identify NCDN as a regulator of U5 snRNP biogenesis. We show that NCDN associates with PRPF8–AAR2–EFTUD2 complexes in the cytoplasm and is essential for the proper progression of this assembly intermediate toward mature U5 snRNP formation. Loss of NCDN leads to the accumulation of PRPF8–AAR2 assembly intermediates and a reduction in mature U5 snRNP levels. Furthermore, NCDN deficiency inhibits tumor cell proliferation and induces apoptosis, concomitant with widespread alternative splicing defects, including those affecting apoptosis-related transcripts. Collectively, our results define NCDN as a chaperone factor essential for U5 snRNP biogenesis and highlight its potential as a therapeutic target in splicing-addicted cancers with elevated NCDN expression.

## Materials and methods

### Ethics statement

Human glioma samples and normal brain samples were obtained from patients and donors under a protocol approved by Institutional Review Board at Huashan Hospital, Fudan University (KY2015-256). Written informed consent was obtained from all participants in accordance with institutional guidelines and the Declaration of Helsinki.

### Antibodies

Primary antibodies used for immunoblotting and immunofluorescence were anti-Coilin (clone F-7, Cat.# sc-55594) from Santa Cruz Biotechnology; anti-SNRNP200 (Cat.# A6063), anti-EFTUD2 (Cat.# A7040), anti-AAR2 (Cat.# A18443), anti-PRPF8 (Cat.# A6053), anti-PRPF4 (Cat.# A6052), anti-PRPF3 (Cat.# A5482), anti-PRPF19 (Cat.# A12590), anti-SART3 (Cat.# A12124), anti-SF3B1 (Cat.# A9737), anti-TXNL4A (Cat.# A10138), and anti-U2AF1 (Cat.# A13166) from ABclonal; anti-EFTUD2 (Cat.# 10208-1-AP), anti-NCDN (Cat.# 13187-1-AP), and anti-β-Actin (Cat.# 60008-1-Ig) from Proteintech; anti-FLAG (clone M2, Cat.# F1804), anti-FLAG (Cat.# F7425), anti-GST (Cat.# G1160), and anti-GAPDH (clone 6C5, Cat.# MAB374) from Sigma–Aldrich; anti-HA (clone C29F4, Cat.# 3724), anti-His (clone 27E8, Cat.# 2366), and anti-Histone H3 (Cat.# 9715S) from Cell Signaling Technology. Secondary antibodies used for immunoblotting were: Peroxidase-conjugated AffiniPure Goat Anti-Rabbit IgG (H + L) (Cat.# 111-035-003, Jackson Lab), and Peroxidase-conjugated AffiniPure Goat Anti-Mouse IgG (H + L) (Cat.# 115-035-003, Jackson Lab). Secondary antibodies used for immunofluorescence were: DyLight 594 anti-Rabbit IgG (Cat.# A23410, Abbkine), DyLight 488 anti-Rabbit IgG (Cat.# 35552, Thermo Fisher), and DyLight 594 anti-Mouse IgG (Cat.# 35510, Thermo Fisher).

### Cell lines and culture

EL4, HeLa, U251, U87, and HEK293T cell lines were purchased from the American Type Culture Collection. Cells were maintained in Dulbecco’s modified Eagle’s medium supplemented with 10% fetal bovine serum, 100 U/ml of penicillin and 100 µg/ml of streptomycin. All cells were cultured in a humidified incubator at 37°C with 5% CO_2_. Mycoplasma contamination was not detected in any of the cell lines. Cell line authentication was confirmed by short tandem repeat (STR) profiling.

### Plasmids and shRNA constructs

Flag- and HA-tagged expression plasmids were generated by inserting the corresponding coding sequence (CDS) into pcDNA6-N-Flag and pcDNA6-N-3 × HA vectors, respectively. EGFP-fusion constructs were generated by inserting the CDS into pLV-N-EGFP vector. GST- and His-tagged constructs were generated by inserting the CDS into pGEX-4T-1 and pET-28a (+) vectors, respectively. Lentiviral knockdown plasmids were constructed by inserting the annealed shRNAs into pLV-H1-EF1α-puro vector (Cat.# SORT-B19; Biosettia). The short-hairpin RNA (shRNA) sequences specifically targeting *NCDN* were: 5′-GAATGACAGCGAGCAGTTTGC-3′ (sh*NCDN*#1), 5′-GAAGGAGCCCTTTGTGTTTGC-3′ (sh*NCDN*#2), and 5′- GCAAGTATTTCCTGCAGCAGT-3′ (sh*NCDN*#3). The nontargeting control shRNA sequence was 5′-CAACAAGATGAAGAGCACC-3′ (shCtrl). Lentiviral expression plasmids were constructed by inserting the CDS into pLV-EF1α-MCS-IRES-puro vector (Cat.# cDNA-pLV01; Biosettia). All constructs were verified by Sanger sequencing.

### Lentiviral production and infection

Lentivirus was produced in HEK293T cells by co-transfecting the indicated lentiviral expression vectors with the packaging plasmids pMDLg/pRRE, pRSV-REV, and VSV-G using calcium phosphate–mediated transfection. Viral supernatants were collected 48 h after transfection and cleared by centrifugation at 500 × *g* for 5 min before use. For lentiviral infection, target cells were incubated with viral supernatants in the presence of polybrene (8 μg/ml). To enhance infection efficiency, plates were centrifuged at 1500 × *g* for 30 min.

### Immunoprecipitation and mass spectrometry analysis

Cells were washed with ice-cold phosphate-buffered saline (PBS) and lysed in buffer containing 25 mM Tris–HCl (pH 7.4), 150 mM NaCl, 1% NP-40, 1 mM EDTA, 1 mM EGTA, 2.5 mM sodium pyrophosphate, 1 mM β-glycerophosphate, 1× protease, and phosphatase inhibitor cocktail. Lysates were incubated on ice for 30 min and sonicated for 15 s. Cell lysates were centrifuged at 12 000 × *g* for 15 min at 4°C. 10% of the supernatant was saved as input. For immunoprecipitation of Flag-tagged proteins, the supernatant was incubated with anti-Flag M2 magnetic beads (Sigma‒Aldrich) at 4°C for 4 h or overnight on a rotor. After immunoprecipitation, beads were washed four times with lysis buffer. Then, 3 × Flag peptide (Sigma‒Aldrich) was added to elute the bound proteins from beads. Eluates were resolved by SDS‒PAGE and detected by immunoblotting or silver staining. To identify NCDN-interacting candidates, protein samples were separated by SDS–PAGE, and gel bands were subjected to semi-quantitative mass spectrometry (liquid chromatography–tandem mass spectrometry) analysis by the Proteomics Core Facility at the Institutes of Biomedical Sciences, Fudan University. All NCDN-interacting candidates are listed in Supplementary Data sheet S1.

For endogenous immunoprecipitation, cell lysates were clarified by centrifugation, and the supernatants were incubated overnight at 4°C with the indicated antibodies or control IgG under gentle rotation. On the following day, Protein A/G magnetic beads (Sera-Mag™ SpeedBeads, Cytiva) were added to the antibody–lysate mixtures and incubated for 1 h at room temperature with continuous mixing. Beads were then washed three times with wash buffer (25 mM Tris–HCl, pH 7.5, 0.65 M NaCl, and 0.05% Tween-20). Bound proteins were eluted using low-pH elution buffer (0.1 M glycine, pH 2.5) for 10 min, and eluates were neutralized by the addition of 1/10 volume of 1 M Tris–HCl (pH 9.0). Eluted proteins were analyzed by SDS–PAGE and immunoblotting.

### Immunoblotting

Protein concentrations were quantified using a bicinchoninic acid (BCA) assay. Lysates were resolved by SDS‒PAGE and transferred to NC membranes. Membranes were blocked with 5% nonfat milk in Tris-buffered saline containing 0.1% Tween-20 (TBST) for 1 h at room temperature and then incubated with primary antibodies overnight at 4°C. After washing with TBST, membranes were incubated with horseradish peroxidase (HRP)-conjugated secondary antibodies for 1 h at room temperature, the protein bands were visualized using an enhanced chemiluminescence (ECL) system according to the manufacturer’s protocol.

### GST pulldown assay

GST-tagged proteins were expressed and purified as previously described [35]. Briefly, BL21 (DE3) bacteria were grown in LB medium to an OD600 of 0.8‒1. Then, the cultures were induced overnight at 18°C with 0.2 mM isopropyl β-d-1-thiogalactopyranoside (Sigma‒Aldrich). Bacterial pellets were lysed by sonication in PBS supplemented with protease inhibitors, followed by centrifugation at 12 000 × *g* for 15 min at 4°C. The supernatants were incubated with glutathione agarose beads (Thermo) on a rotating mixer for 2 h at 4°C. The beads were washed three times with PBS and used for subsequent pulldown assays. His-tagged proteins were purified using Ni-NTA resin and eluted with imidazole. For pulldown assays, purified His-tagged proteins were incubated with GST or GST-tagged proteins bound to glutathione agarose beads in binding buffer (0.1% NP40/PBS) for 4 h at 4°C with rotation. After washing, bound proteins were eluted with elution buffer (50 mM Tris‒HCl, pH 8.0, 10 mM reduced glutathione). The samples were boiled at 95°C for 3 min and analyzed by SDS–PAGE followed by immunoblotting.

### Immunofluorescence and fluorescence *in situ* hybridization

Cells were cultured on coverslips, fixed with 4% paraformaldehyde (PFA) for 15 min at room temperature (RT), and washed three times with PBS. Cells were permeabilized with 0.2% Triton X-100/PBS for 10 min on ice and washed three times with PBS. Samples were then blocked in 1% bovine serum albumin (BSA)/PBS for 1 h at RT. Coverslips were incubated overnight at 4°C with primary antibodies diluted (1:200) in 1% BSA/PBS. After washing with PBS, secondary antibodies (1:500) were applied for 1 h at room temperature in the dark. Following washing, samples were either mounted directly or further processed for RNA fluorescence *in situ* hybridization (FISH). U5 snRNA was detected using fluorescent *in situ* hybridization as previously described [20]. DNA probes were 5′-labeled with fluorescein amidite (FAM), and the sequence is 5′-CTCTCCACGGAAATCTTTAGTAAAAGGCGAAAGATTT ATACGATTTGAAGAG-3′. Cells were post-fixed in 4% PFA/PIPES for 10 min, followed by sequential treatment with 0.1 M glycine/0.2 M Tris–HCl (pH 7.4) for 10 min and 50% formamide/2× SSC for at least 10 min. Hybridization was performed in 50% formamide/10% dextran sulfate/1% BSA/2× SSC with 2 μM probe at 37°C for ≥2 h. Coverslips were washed sequentially in 50% formamide/2× SSC at 37°C for 20 min, 2× SSC at 37°C for 20 min, and 1× SSC at room temperature for 20 min. All coverslips were mounted with Fluoromount-G containing 4′,6-diamidino-2-phenylindole (DAPI). Images were captured using a LSM900 Zeiss confocal microscope system.

### mRNA library constructing, sequencing, and data analysis

One microgram of total RNA was used for library preparation. The poly(A) mRNA isolation was performed using Oligo(dT) beads. The mRNA fragmentation was performed using divalent cations and high temperature. Priming was performed using Random Primers. First strand cDNA and the second-strand cDNA were synthesized. The purified double-stranded cDNA was then treated to repair both ends and add a dA-tailing in one reaction, followed by a T-A ligation to add adaptors to both ends. Size selection of Adaptor-ligated DNA was then performed using DNA Clean Beads. Each sample was then amplified by PCR using P5 and P7 primers and the PCR products were validated. Then, libraries with different indexs were multiplexed and loaded on an Illumina HiSeq instrument for sequencing using a 2 × 150 paired-end (PE) configuration according to manufacturer’s instructions. Pass filter data in fastq format were processed by Cutadapt (v1.9.1, phred cutoff: 20, error rate: 0.1, adapter overlap: 1 bp, min. length: 75, proportion of *N*: 0.1) to obtain high-quality clean data. Clean data were aligned to reference genome via software Hisat2 (v2.2.1). Differential expression analysis of the two conditions was performed using the edgeR R package (3.22.5). *P*-values were adjusted using the Benjamini and Hochberg method. *P *< 0.05 and Fold change > 1.5 were set as the thresholds for significantly differential expression. rMATS (4.1.0) software was used to analyze the alternative splicing events. The alternative splicing events and differentially expressed genes (DEGs) are listed in Supplementary Data sheet S2 and S3, respectively.

### Gene Ontology (GO) enrichment analysis

Gene Ontology (GO) enrichment analysis was performed using DAVID database (https://davidbioinformatics.nih.gov/). For the analysis of the protein interaction dataset, the input list consisted of proteins in Supplementary Data Sheet S1, including those classified as “NCDN only” or with a ratio > 2. For the analysis of the differentially expressed gene dataset, the input list was derived from Supplementary Data Sheet S3. GO Biological Process terms were analyzed using the default human background provided by DAVID. Terms with FDR < 0.05 were considered statistically significant. Enriched GO terms were ranked based on a combination of statistical significance (FDR) and gene count. Full GO term annotations and corresponding gene lists are provided in the Supplementary Data Sheet S4.

### Quantitative real-time PCR (RT-qPCR) and reverse-transcription PCR (RT-PCR)

Total RNA was extracted using TRIzol reagent (Thermo) and reverse-transcribed into cDNA. RT-qPCR was performed with 2× SYBR Green PCR Master Mix (Vazyme) on a QuantStudio1 Real-Time PCR system (Applied Biosystems). Fold changes were calculated by relative quantification (2^–ΔΔCt^). Semi-quantitative RT-PCR and a Tanon Bio-Fragment Analyzer (BiOptic) were used to analyze alternative spliced products. Primer sequences were designed to target the constitutively expressed flanking exons, and 2× Taq Plus Master Mix (Vazyme) was used to synchronously amplify isoforms that included or skipped the target exon. The primer sequences are listed in Supplementary Tables S1 and S2.

### Subcellular fractionation

For nuclear extract preparation used in glycerol gradient (10%–30%) fractionation, cells were lysed in hypotonic buffer (10 mM Tris–HCl, pH 7.9, 1.5 mM MgCl_2_, 10 mM KCl, 1 mM dithiothreitol (DTT), 1 mM phenylmethylsulfonyl fluoride (PMSF), and 1× protease inhibitor cocktail) by a Dounce homogenizer with 40 strokes. Nuclear pellets were separated from cytoplasmic fraction by centrifugation at 1000 × *g* for 10 min at 4°C. The supernatants which contain the cytoplasmic extract were carefully removed and transferred into new tubes. Nuclear pellets were washed twice with hypotonic buffer and subsequently resuspended in the nuclear extraction buffer (50 mM Tris–HCl, pH 7.9, 1 mM MgCl_2_, 1 mM DTT, 0.1% NP-40, 1 mM PMSF, and 1× protease inhibitor cocktail). The nuclear extracts were acquired in the supernatants by centrifuging the sonicated mixture at 17 000 × *g* for 10 min at 4°C. For routine subcellular fractionation, cells were lysed in 0.1% NP-40 in PBS by gentle pipetting and incubated on ice for 1 min to release the cytoplasmic fraction. The lysates were centrifuged at 10 000 × *g* for 20–30 s at 4°C and the supernatant containing the cytoplasmic fraction was collected. The nuclear pellet was washed twice with PBS and centrifuged at 10 000 × *g* for 20–30 s at 4°C. The pellet was then lysed in buffer containing 25 mM Tris–HCl (pH 7.4), 150 mM NaCl, 1% NP-40, 1 mM EDTA, 1 mM EGTA, 2.5 mM sodium pyrophosphate, 1 mM β-glycerophosphate, sonicated, and centrifuged at 12 000 × *g* for 10 min at 4°C to obtain the nuclear extract.

### Glycerol-gradient (10%−30%) fractionation

To analyze snRNP distribution by glycerol gradient fractionation, 200 μg of NCDN-depleted or control HeLa or U251 nuclear extracts were diluted in 200 μl of IP150 buffer (20 mM HEPES, pH 7.9, 150 mM NaCl, 1.5 mM MgCl_2_, and 0.5 mM DTT, supplemented with RNase inhibitor, protease and phosphatase inhibitors) and then sedimented onto a linear 4 ml of 10%−30% (v/v) glycerol gradient prepared with IP150 buffer. The samples were then centrifuged at 29 000 rpm (~114 000 × *g*) for 14 h at 4°C with a Sorvall TH-660 rotor, and the gradients were separated into 24 equal fractions. 4 × SDS sample buffer was added to each fraction and the proteins were subjected to SDS−PAGE and immunoblot analysis. In parallel, aliquots of each fraction were subjected to RNA extraction using TRIzol reagent, followed by reverse transcription and RT-qPCR analysis to assess the distribution of snRNAs across the gradient.

### Colony formation assay

Cancer cells with NCDN overexpression or knockdown were seeded in six-well plates (1000 cells per well) and cultured for 1−2 weeks. Colonies were fixed with 4% PFA and stained with 0.1% crystal violet. The number of colonies was counted using ImageJ.

### Cell proliferation analysis

Three days after infection, cells were plated at a density of 1000 cells per well in 96-well plate. Cell number was assessed every 48 h for 6 days using the CCK-8 kit (Beyotime) according to the manufacturer’s instructions.

### Apoptosis assay

Floating and adherent cells were collected and centrifuged. Then, the cells were incubated for 15 min at room temperature in the dark with 400 µl of Annexin V binding buffer, including propidium iodide (PI) solution and FITC-conjugated Annexin V. Stained cells were detected by flow cytometry (BD Biosciences), and the data were analyzed using FlowJo v10 software (Tree Star).

### Software and algorithms

GraphPad Prism v8.0 (GraphPad), ZEN 3.5 (Zeiss), QuantStudio™ Design and Analysis Software v1.5.1 (Applied Biosystems), FlowJo v10 (Tree Star), ImageJ v1.8.0, BioRender.

### Statistical analysis

Data in the study are presented as means ± SD and analyzed statistically using GraphPad Prism 8.0 software. *P*-values were calculated using unpaired two-tailed Student’s *t*-test between two groups and one-way or two-way ANOVA for multiple groups. *P *< 0.05 was defined as significantly different.

## Results

### NCDN associates with U5 snRNP and directly binds to AAR2

**Figure 1.**
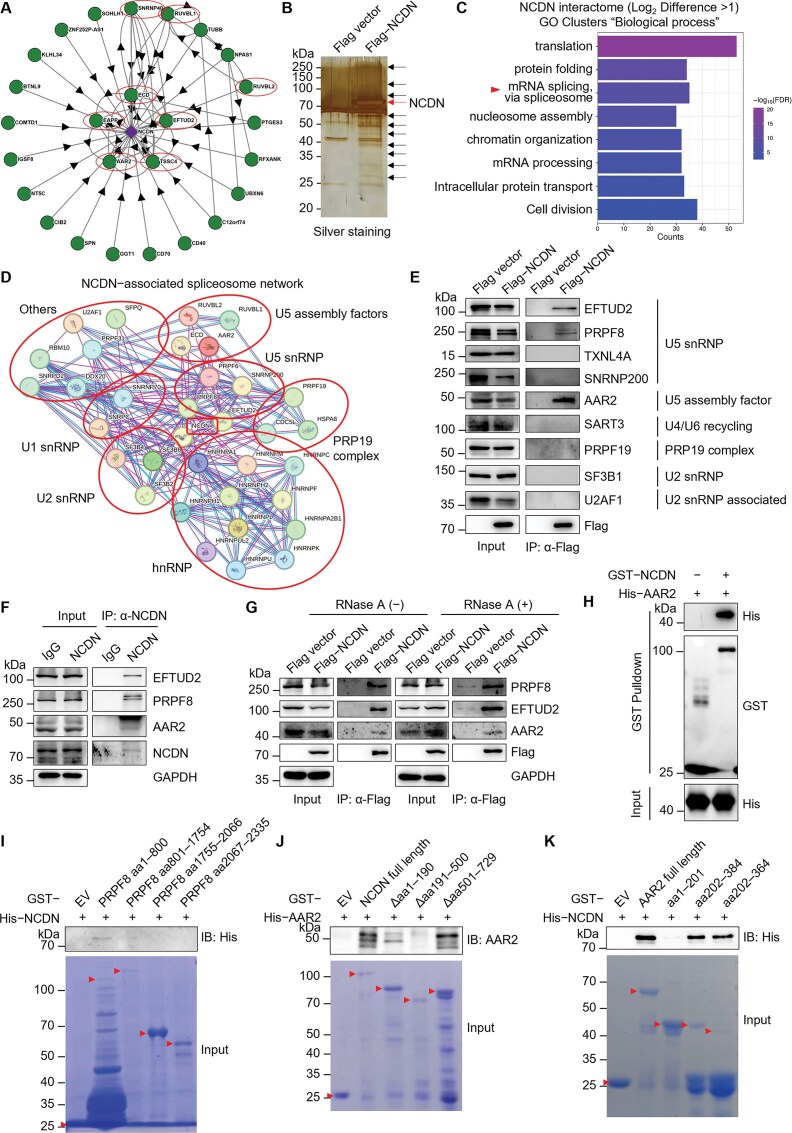
NCDN associates with the U5 snRNP and directly interacts with AAR2 and PRPF8. (**A**) NCDN-interacting proteins revealed by the BioPlex dataset (https://bioplex.hms.harvard.edu). Splicing-related proteins are highlighted with circles. (**B**) HeLa cells were infected with lentivirus expressing Flag (vector) or Flag-tagged NCDN. Cells from three independent lentiviral transductions were subjected to immunoprecipitation separately using anti-Flag beads, and the eluates were pooled for subsequent analysis. Bound proteins were analyzed by silver staining. Arrows indicate protein bands specifically present in the NCDN sample. (**C**) Gene Ontology (GO) enrichment analysis of NCDN-interacting proteins identified by co-immunoprecipitation followed by mass spectrometry (Co-IP/MS). (**D**) Spliceosome-associated protein interaction network enriched among NCDN-binding partners identified by Co-IP/MS, visualized using STRING database analysis. Circles indicate proteins that are part of the same complex. (**E**) HeLa cell lysates from Flag (vector) or Flag-tagged NCDN infected cells were immunoprecipitated with anti-Flag beads and analyzed by immunoblotting (IB) with the indicated antibodies. (**F**) Endogenous NCDN was immunoprecipitated from U251 cell lysates using anti-NCDN antibodies or control IgG, and co-precipitated proteins were detected by immunoblotting. (**G**) U251 cell lysates, either untreated or treated with RNase A (100 μg/ml, 1 h at 37°C) were immunoprecipitated with anti-Flag beads and analyzed by immunoblotting with the indicated antibodies to assess RNA-dependent interactions. (**H**) Bacterially expressed GST or GST-tagged NCDN proteins were incubated with purified His-tagged AAR2, followed by GST pulldown. Bound proteins were detected by IB using for anti-His and anti-GST antibodies. (**I**) GST pulldown assays were performed using different GST-tagged PRPF8 truncation constructs and purified His-NCDN. Upper panel, detection of His-NCDN co-precipitated with each GST-PRPF8 fragment (IB: His). Lower panel, Coomassie-stained gel showing the expression and loading of GST-PRPF8 fusion proteins. Arrows indicate the positions of the target proteins. PRPF8 (aa 1–800) showed relatively low stability during bacterial expression and purification, requiring increased bacterial input to achieve detectable levels. aa, amino acid. (**J**) GST pulldown assays were performed using different GST-tagged NCDN truncation constructs and purified His-AAR2. Upper panel, detection of His-AAR2 co-precipitated with each GST-NCDN fragment (IB: AAR2). Lower panel, Coomassie-stained gel showing expression and loading of GST-NCDN fusion proteins. (**K**) GST pulldown assays were performed using different GST-tagged AAR2 truncation constructs and purified His-NCDN. Upper panel, detection of His-NCDN co-precipitated with each GST-AAR2 fragment (IB: His). Lower panel, Coomassie-stained gel showing expression and loading of GST-AAR2 fusion proteins.

Our previous study identified an interaction between NCDN and SNRNP40, a core component of the U5 snRNP (Supplementary Fig. S1A), suggesting a potential role for NCDN in spliceosome regulation. To further investigate the function of NCDN, we analyzed publicly available protein–protein interaction databases, which revealed that the NCDN interactome is enriched for multiple spliceosomal components (Fig. 1A, and Supplementary Fig. S1B and C). To systematically identify NCDN-associated proteins, we performed co-immunoprecipitation combined with mass spectrometry (Co-IP/MS) (Fig. 1B and Supplementary Fig. S1D; Supplementary Data sheet S1). Gene Ontology (GO) enrichment analysis revealed that NCDN-associated proteins are enriched in splicing-related processes (Fig. 1C). The splicing-related subnetwork indicated a predominant association of NCDN with U5 snRNP components. Notably, several known U5 snRNP chaperones—including AAR2, RUVBL1/2, and ECD—were identified among the interactors (Fig. 1D and Supplementary Fig. S1E).

The mass spectrometry results were validated by immunoblotting, confirming that NCDN interacts with core U5 proteins (PRPF8 and EFTUD2), as well as the chaperone AAR2 (Fig. 1E and F, and Supplementary Fig. S1F). To determine whether these interactions are RNA-dependent, cell lysates were treated with RNase prior to immunoprecipitation. The associations between NCDN and U5 components remained intact, indicating that the interactions are RNA-independent (Fig. 1G).

GST pulldown assays further demonstrated that NCDN directly binds to AAR2 but not EFTUD2 (Fig. 1H and Supplementary Fig. S1G). To investigate whether NCDN directly interacts with PRPF8, we performed pulldown assays using truncated PRPF8 fragments due to the large size of the full-length protein [14, 36]. PRPF8 was divided into four fragments based on its domain architecture, and we found that the N-terminal region (amino acids 1–800) mediates its interaction with NCDN (Fig. 1I and Supplementary Fig. S1H). To further dissect the structural domains of NCDN required for its interactions with AAR2, we constructed NCDN deletion mutants based on protein structure predictions generated by AlphaFold (Supplementary Fig. S1I). Deletion of amino acids 191–500 significantly reduced NCDN’s interaction with AAR2 (Fig. 1J). We next examined the structural basis of AAR2 involved in this interaction. Based on previous studies, AAR2 was divided into an N-terminal and a C-terminal fragment [37]. We found that deletion of the C-terminal region (amino acids 202–384) impaired its interaction with NCDN (Fig. 1K). Moreover, previous studies have shown that the extreme C-terminal tail of AAR2 (amino acids 364–384) is required for its interaction with PRPF8 [37]. We therefore examined whether this region is also necessary for binding to NCDN. Notably, deletion of amino acids 364–384 did not affect the interaction between AAR2 and NCDN (Fig. 1K).

Collectively, these results establish NCDN as a U5 snRNP-associated factor that directly interacts with U5 snRNP component PRPF8 and the chaperone AAR2, supporting its potential role in U5 snRNP assembly.

### NCDN colocalizes with U5-associated proteins in the cytoplasm

**Figure 2.**
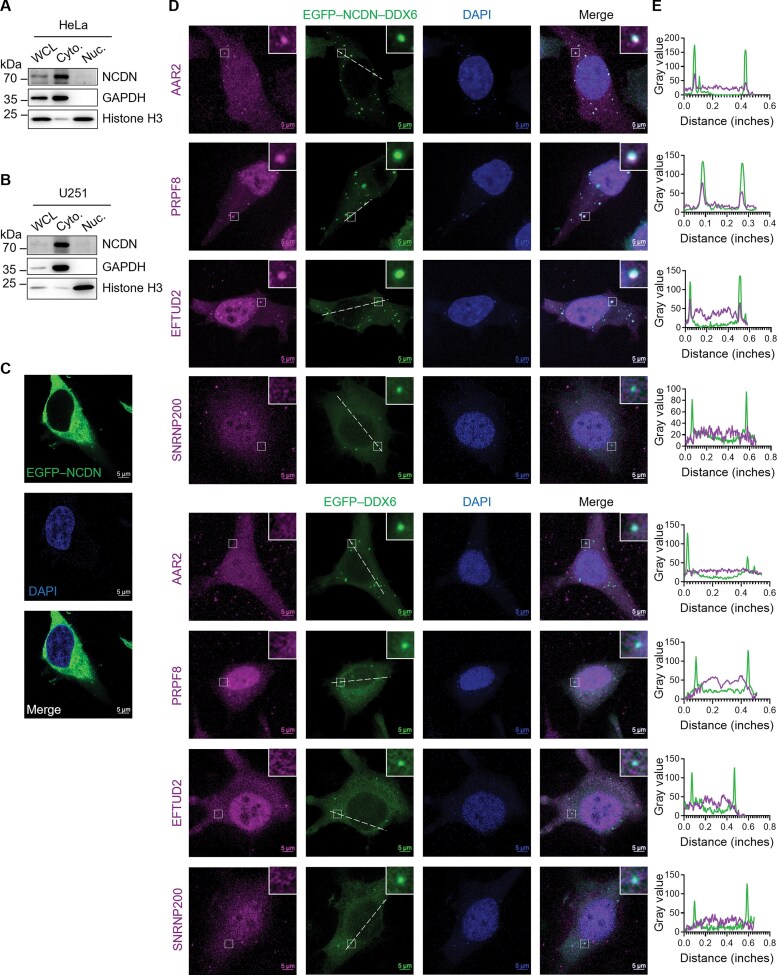
NCDN colocalizes with PRPF8, EFTUD2, and AAR2 in the cytoplasm. (**A** and **B**) HeLa (A) and U251 (B) cells were fractionated into whole cell lysates (WCL), cytoplasmic (Cyto), and nuclear (Nuc) fractions, followed by immunoblotting to assess the subcellular distribution of NCDN. Histone H3 and GAPDH were used as markers for nuclear and cytoplasmic fractions, respectively. (**C**) HeLa cells were infected with lentivirus expressing EGFP-tagged NCDN. Nuclei were stained with DAPI (blue). Scale bar: 5 μm. (**D**) HeLa cells were infected with lentivirus expressing EGFP–NCDN–DDX6 fusion protein or EGFP–DDX6 (green). The DDX6/p54 peptide directs EGFP–NCDN–DDX6 protein into cytoplasmic P-bodies. Endogenous U5 proteins (AAR2, PRPF8, EFTUD2, and SNRNP200) were stained by corresponding antibodies (magenta; red/green fluorescence was recolored to magenta/green for color-blind accessibility). Co-recruitment of individual U5 proteins into P-bodies indicates their interaction with NCDN. The dashed line indicates the region of interest (ROI) used for fluorescence intensity profiling. Scale bar: 5 μm. (**E**) Quantitative colocalization analysis between NCDN and U5 snRNP proteins was performed using ImageJ.

The interaction of NCDN with AAR2 and PRPF8 suggests that NCDN may function at an early stage of U5 snRNP biogenesis. Subcellular fractionation analysis revealed that NCDN was predominantly distributed in the cytoplasm (Fig. 2A and B). Consistently, immunofluorescence analysis further confirmed its mainly cytoplasmic distribution (Fig. 2C).

To determine whether NCDN interacts with U5-associated proteins in the cytoplasm, we performed a tethering assay by fusing NCDN with DDX6/p54, which targets the fusion protein to P-bodies [38], along with EGFP to monitor localization. We expressed the fusion protein EGFP–NCDN–DDX6 in HeLa cells to detect co-localization with endogenous U5 snRNP-associated proteins and we observed strong P-body localization of AAR2, PRPF8, and EFTUD2 (Fig. 2D and E), indicating that NCDN associates with U5 snRNP components during their biogenesis in the cytoplasm. While SNRNP200 was not recruited to p54-NCDN P**-**bodies, this observation suggests that SNRNP200 may not stably associate with these early cytoplasmic intermediates and is instead incorporated at a later stage, likely during or after nuclear import.

Collectively, these results support a model in which NCDN engages with early U5 snRNP assembly intermediates in the cytoplasm prior to their nuclear maturation.

### NCDN deficiency reduces the level of mature U5 snRNP

**Figure 3.**
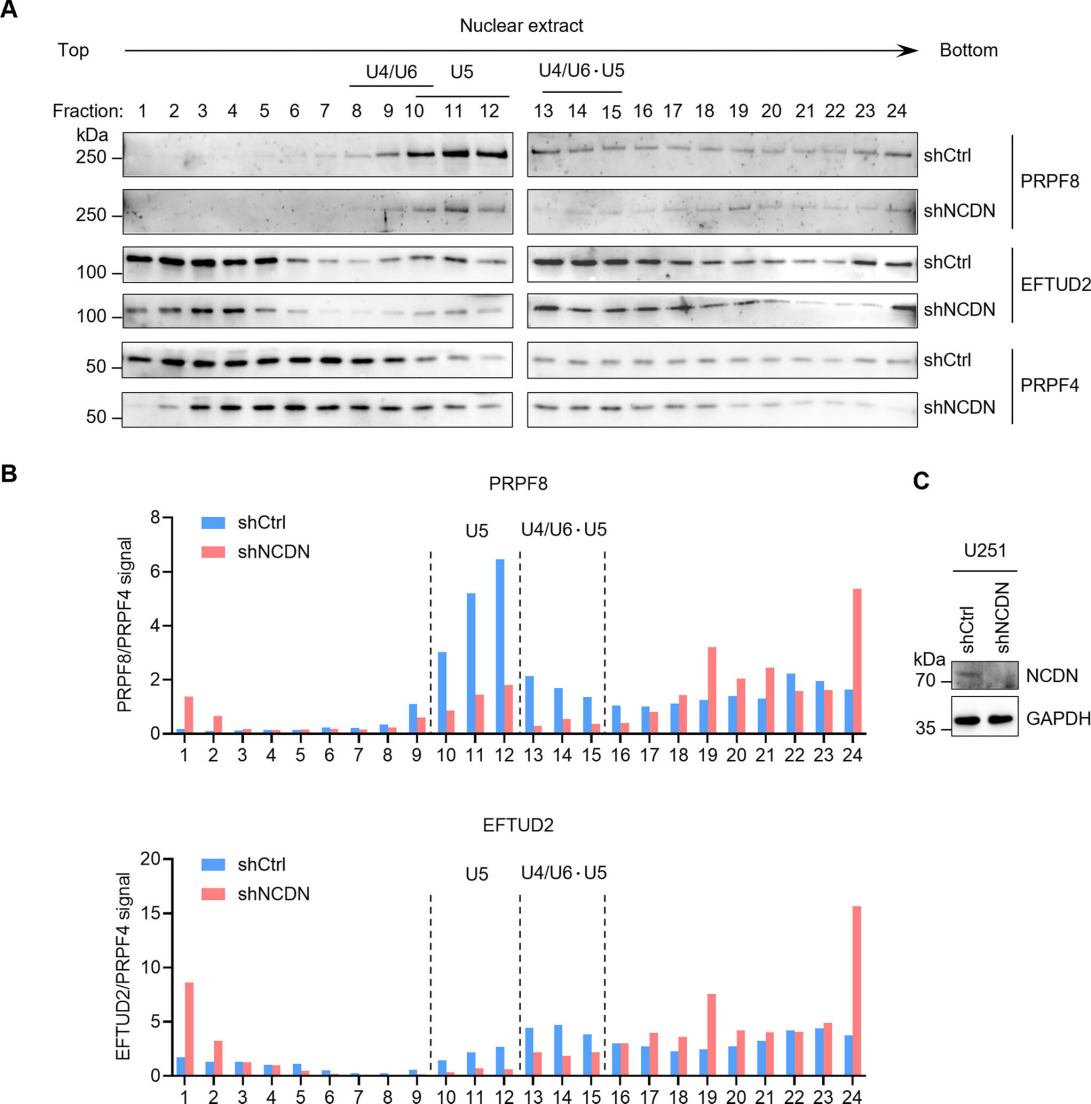
NCDN deficiency hinders U5 snRNP biogenesis. (**A**) Nuclear extracts from control (shCtrl) and NCDN-knockdown (shNCDN) U251 cells were subjected to 10%–30% glycerol gradient centrifugation, followed by immunoblotting with the indicated antibodies to assess the distribution and relative abundance of U5 snRNP components (PRPF8 and EFTUD2) and U4/U6 snRNP components (PRPF4) across the gradient. (**B**) Immunoblot band intensities from panel (A) were measured using ImageJ. For each fraction, the intensity of the indicated protein was normalized to the intensity of PRPF4 within the same fraction, which served as an internal control due to its stable expression across different samples. The graph shows the distribution of representative U5 snRNP proteins from a representative experiment. (**C**) Immunoblot analysis of NCDN expression in lysates from U251 cells infected with lentivirus encoding control shRNA (shCtrl) or shRNA targeting NCDN (shNCDN), showing NCDN knockdown efficiency in panel (A).

Given the NCDN interactome, we hypothesized that NCDN may play a role in U5 snRNP metabolism. To test whether NCDN is required for U5 snRNP and subsequently U4/U6·U5 tri-snRNP assembly, we knocked down NCDN and analyzed snRNP complexes by glycerol gradient ultracentrifugation. NCDN deficiency led to a reduction in U5 snRNP and U4/U6·U5 tri-snRNP, which are represented by PRPF8 and EFTUD2 (Fig. 3A–C and Supplementary Fig. S2A–C). To further assess snRNP integrity at the RNA level, we isolated RNA from gradient fractions and quantified snRNAs by RT-qPCR. Consistent with the protein-based analysis, we observed a global reduction in U4, U5, and U6 snRNAs in NCDN-depleted extracts (Supplementary Fig. S2D), supporting a defect in U5 snRNP and tri-snRNP assembly.

### Depletion of NCDN impairs AAR2 release and arrests U5 snRNP assembly at an intermediate stage

Previous studies demonstrated that the HSP90/R2TP complex can bind unassembled U5 proteins in the cytoplasm, stabilize them, and promote the formation of the U5 snRNP [14]. The zinc-finger protein ZNHIT2 serves as a bridge between U5 snRNP and R2TP complex [16]. Our NCDN interactome revealed substantial amounts of both the R2TP/PFDL complex and the U5 snRNP proteins (Supplementary Fig. S3A). We further confirmed direct binding of NCDN to R2TP complex components RUVBL1 and RUVBL2 (Supplementary Fig. S3B), raising the possibility that NCDN might act as a bridging factor between the U5 snRNP and R2TP complex. This hypothesis was tested by Co-IP experiments in control and NCDN-depleted cells (Supplementary Fig. S3C and D). Flag-tagged RUVBL1 or RUVBL2 were independently purified in both cases, and association with U5 snRNP components EFTUD2 was confirmed. However, when combined with NCDN knockdown, R2TP/PFDL subunits co-purified the same amount of EFTUD2 proteins as compared to control cells, supporting that NCDN is not essential for mediating the interaction between these complexes under the conditions tested.

**Figure 4.**
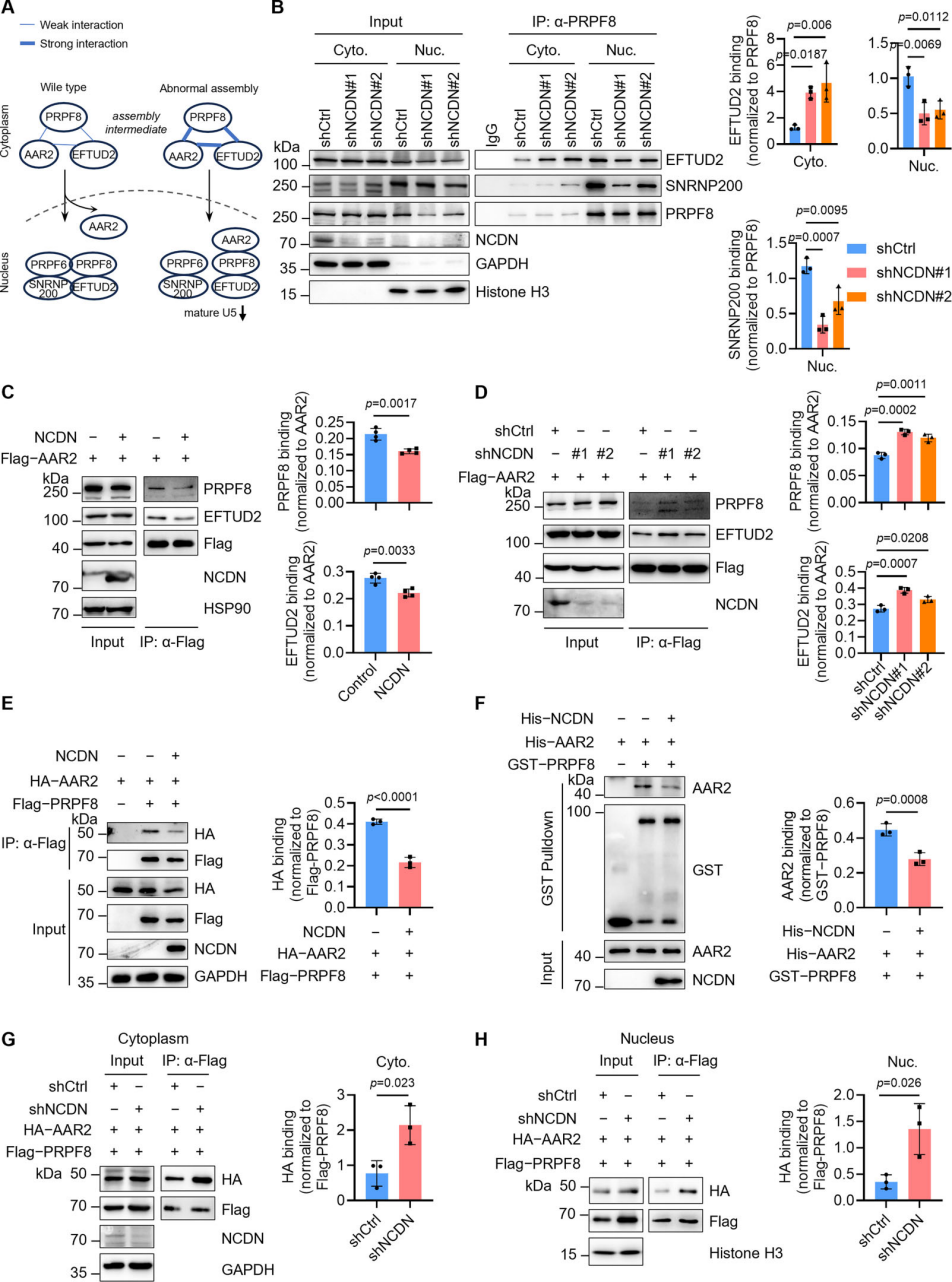
NCDN deficiency stabilizes PRPF8–AAR2–EFTUD2 intermediates and impairs U5 snRNP maturation. (**A**) Schematic illustration of the U5 snRNP assembly intermediate. Enhanced interactions between PRPF8 and EFTUD2/AAR2 are associated with reduced binding of PRPF8 to other U5-specific proteins, as previously described (Malinová *et al.*, 2017). (**B**) U251 cells were fractionated into nuclear and cytoplasmic compartments, followed by immunoprecipitation with anti-PRPF8 antibody. Co-immunoprecipitated proteins were detected by immunoblotting with the indicated antibodies. Quantification of the immunoprecipitated proteins is shown on the right. (**C** and **D**) Immunoprecipitation of overexpressed Flag-tagged AAR2 from HEK293T cells transfected with vector or NCDN (C) or infected with lentivirus encoding control shRNA (shCtrl) or NCDN-targeting shRNA (shNCDN) (D). Interacting endogenous proteins were detected by immunoblotting. Quantification of co-immunoprecipitated proteins is shown on the right. (**E**) HEK293T cells were co-transfected with Flag-tagged PRPF8 (amino acid 1755–2335), HA-tagged AAR2, with or without NCDN. Cell lysates were subjected to anti-Flag immunoprecipitation and analyzed by immunoblotting with the indicated antibodies. Quantification of the co-precipitated proteins is shown on the right. (**F**) Bacterially expressed GST or GST-tagged PRPF8 (amino acid 1755–2335) proteins were incubated with His-tagged AAR2 in the presence or absence of His-tagged NCDN. GST pulldown was performed followed by immunoblotting with the indicated antibodies. Quantification of bound proteins is shown on the right. (**G** and **H**) HEK293T cells infected with lentivirus encoding control shRNA (shCtrl) or NCDN-targeting shRNA (shNCDN) were co-transfected with Flag-tagged PRPF8 (amino acid 1755–2335) and HA-tagged AAR2. Cell lysates were fractionated into cytoplasmic (G) and nuclear (H) compartments followed by immunoprecipitation with anti-Flag and analyzed by immunoblotting with the indicated antibodies. Quantification of the co-precipitated proteins is shown on the right. Data are shown as mean ± SD. *P*-values were determined by one-way ANOVA with Dunnett’s multiple comparisons test (B and D) or unpaired two-tailed Student’s *t*-test (C and E–H). Data are representative of at least three independent experiments.

In yeast, Prp8 and Snu114 form an assembly intermediate with the chaperone Aar2 before joining U5 snRNA [12]. Similarly, in human cells, enhanced PRPF8–EFTUD2–AAR2 interactions coincide with reduced PRPF8 association with other U5 proteins, indicating formation of a similar assembly intermediate [14] (Fig. 4A). To explore whether NCDN deficiency affects U5 snRNP assembly dynamics, we separated nuclear and cytoplasmic fractions and assessed PRPF8–EFTUD2 binding. PRPF8 was immunoprecipitated from each fraction using anti-PRPF8 antibodies, and co-precipitated proteins were detected by immunoblotting. To quantitatively evaluate the PRPF8 interaction, the amount of coprecipitated proteins was quantified and normalized to the PRPF8 signal. We observed that NCDN loss was associated with increased PRPF8–EFTUD2 binding in the cytoplasmic fraction. In addition, we examined the interaction between PRPF8 and SNRNP200 in the nuclear fraction. Notably, NCDN depletion reduced the association of PRPF8 with SNRNP200 in the nucleus, consistent with impaired formation of mature U5 snRNP complexes (Fig. 4B).

Since AAR2 is predominantly localized in the cytoplasm (Supplementary Fig. S4A–C), we immunoprecipitated Flag-AAR2 to probe cytoplasmic U5 assembly intermediates. We found that overexpression of NCDN diminished the interaction of AAR2 with PRPF8, whereas knockdown of NCDN strengthened this interaction (Fig. 4C and D).

We also performed the pulldown through PRPF8 and observed that overexpression of NCDN similarly reduced the association of PRPF8 with AAR2 (Fig. 4E and F). To further examine cytoplasmic assembly intermediates, we assessed PRPF8–AAR2 binding in cytoplasmic fractions. Consistent with previous results, NCDN depletion was associated with increased interaction between AAR2 and PRPF8 in the cytoplasmic fraction (Supplementary Fig. S4D), suggesting accumulation of the AAR2–PRPF8-containing intermediate. To examine the compartment-specific regulation of the PRPF8–AAR2 interaction, we performed PRPF8 immunoprecipitation using overexpressed tagged PRPF8 following cytoplasmic and nuclear fractionation. Notably, NCDN depletion led to enhanced association between PRPF8 and AAR2 in both the cytoplasmic and nuclear fractions (Fig. 4G and H). Together, these results support a model in which NCDN modulates the stability of the AAR2–PRPF8-containing assembly intermediate and is associated with its progression toward mature U5 snRNP formation.

Previous studies used U5 protein accumulation in Cajal bodies as a surrogate for U5 snRNP assembly and recycling [39, 40]. However, quantification of PRPF8 and EFTUD2 signals in Cajal bodies normalized to the nucleoplasmic signal did not reveal significant changes upon NCDN depletion (Supplementary Fig. S5A and B). In contrast to U5 snRNP proteins, U5 snRNA exhibited accumulation in Cajal bodies upon NCDN depletion (Supplementary Fig. S5C), suggesting that NCDN loss compromises U5 snRNP maturation, resulting in retention of U5 snRNA within Cajal bodies. Previous studies show that defects in tri-snRNP formation are associated with accumulation of U4/U6 components in Cajal bodies [39], we next examined the localization of the U4/U6 associated-proteins PRPF3 and PRPF4. Notably, both PRPF3 and PRPF4 exhibited increased accumulation in Cajal bodies upon NCDN depletion (Supplementary Fig. S5D and E), suggesting a defect in tri-snRNP assembly.

Together, these data suggest that NCDN is involved in regulating the progression of the AAR2-containing U5 assembly intermediate toward mature U5 snRNPs. In the absence of NCDN, this intermediate becomes aberrantly stabilized, leading to defective U5 snRNP maturation and subsequent perturbation of tri-snRNP assembly.

### NCDN deficiency inhibits cell proliferation and induces cell apoptosis

**Figure 5.**
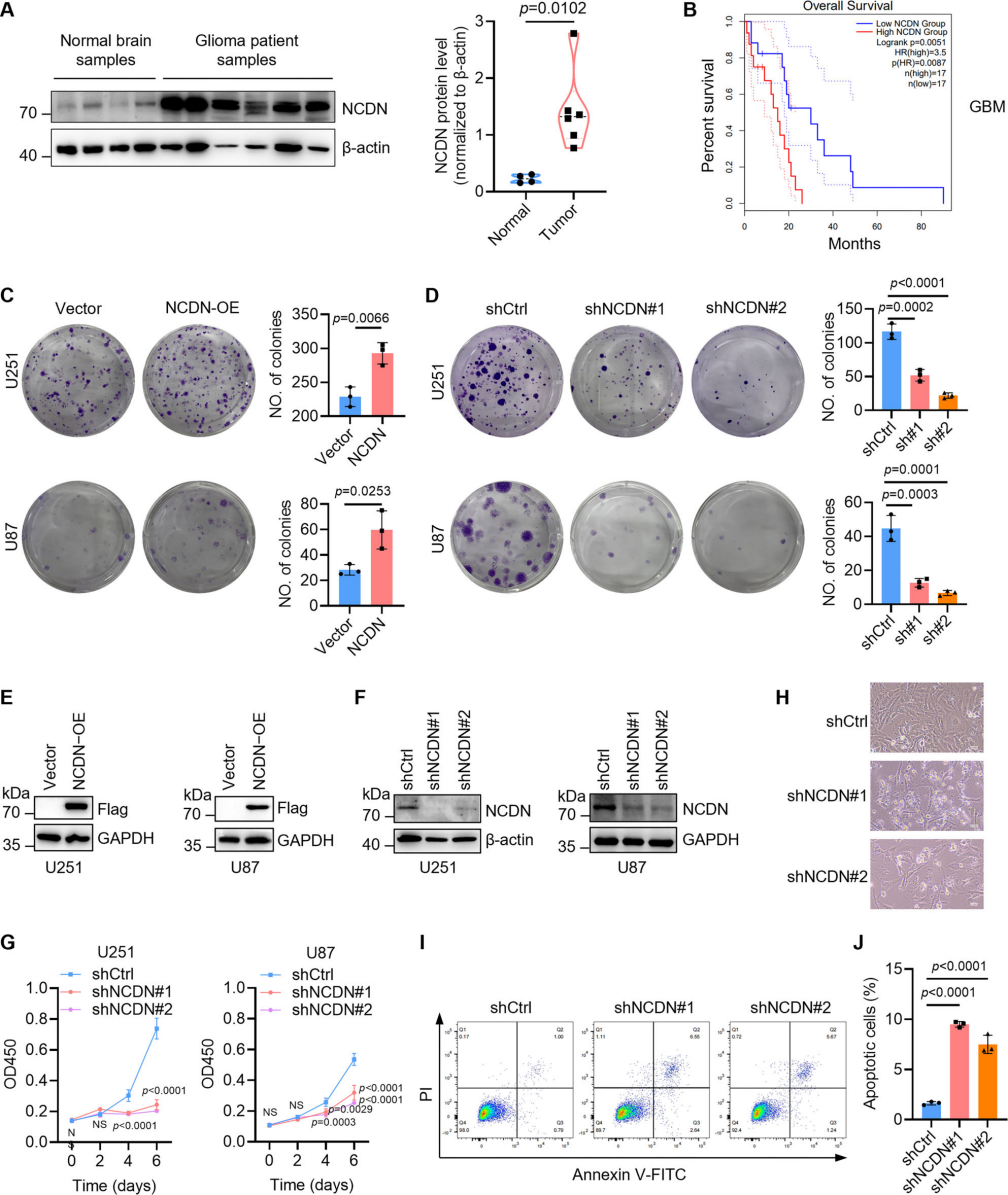
Elevated NCDN expression correlates with poor glioma patient survival and NCDN deficiency inhibits cell proliferation and induces apoptosis. (**A**) NCDN protein levels in lower-grade glioma (LGG) tumor samples (*n* = 6) and normal brain tissues (*n* = 4) were analyzed by immunoblotting. Quantification of NCDN levels is shown on the right. (**B**) Kaplan–Meier curves showing the relationship between NCDN expression and overall survival in GBM. Data were obtained from TCGA and GTEx datasets and plotted by Gepia2. GBM, Glioblastoma. (**C** and **D**) Colony formation assays were performed in U251 and U87 cells to assess the effects of NCDN overexpression (C) and knockdown (D). (**E**) Immunoblot validation of NCDN in U251 and U87 infected with lentivirus expressing Flag (vector) or Flag-tagged NCDN. Cells were harvested 48 h after infection. (**F**) Immunoblot analysis of NCDN knockdown efficiency in U251 and U87 infected with lentivirus encoding control shRNA (shCtrl) or shRNA targeting NCDN (shNCDN). Cells were harvested 48 h after infection. (**G**) Cell proliferation was measured in U251 and U87 cells using the CCK-8 assay. Cell number is expressed as OD450 values. (**H**) Representative brightfield microscopy images of control and NCDN-depleted U251 cells. Scale bars: 100 μm. (**I** and **J**) Representative flow cytometry plots (I) and qualification (J) of apoptotic cells stained with Annexin V-FITC and PI in control and NCDN-depleted U251 cells. Data are presented as mean ± SD from three independent replicates. Statistical analyses were performed using unpaired two-tailed Student’s *t*-test (A and C), one-way ANOVA with Dunnett’s multiple comparisons test (D and J), and two-way ANOVA with Dunnett’s multiple comparisons test (G).

Dysregulation of the spliceosome has emerged as a critical factor in the pathogenesis of various diseases, including cancer [41, 42]. We analyzed NCDN expression across different databases and found that NCDN is highly expressed in brain tissues compared with other organs (Supplementary Fig. S6A–C). To assess the role of NCDN in brain tumor development, we analyzed NCDN expression in clinical glioma samples and found that NCDN is highly expressed in tumor samples compared with controls (Fig. 5A). Consistently, analysis of The Cancer Genome Atlas (TCGA) and Genotype-Tissue Expression (GTEx) datasets revealed that higher NCDN expression is associated with poorer overall survival in glioma patients (Fig. 5B), suggesting a potential link between NCDN and glioma progression.

To examine the possible role of NCDN in tumor cell growth and tumor progression, we stably overexpressed or knocked down NCDN in U251 and U87 glioma cells. Strikingly, we found that NCDN deficiency inhibited tumor cell growth and NCDN overexpression promoted tumor cell growth, as judged by colony formation (Fig. 5C–F). Consistently, cell proliferation assays showed that NCDN deficiency inhibited cell growth (Fig. 5G). Moreover, NCDN deficiency led to more cells undergoing apoptosis (Fig. 5H–J). Together, these findings indicate that NCDN may play a potential pro-tumorigenic role in glioma.

### NCDN deficiency results in widespread splicing abnormalities and gene expression changes

Given that NCDN affects spliceosome assembly, we wondered whether its effect on tumor development is associated with alterations in alternative splicing (AS). To address this, we conducted RNA sequencing (RNA-seq) in NCDN-deficient U251 cells.

**Figure 6.**
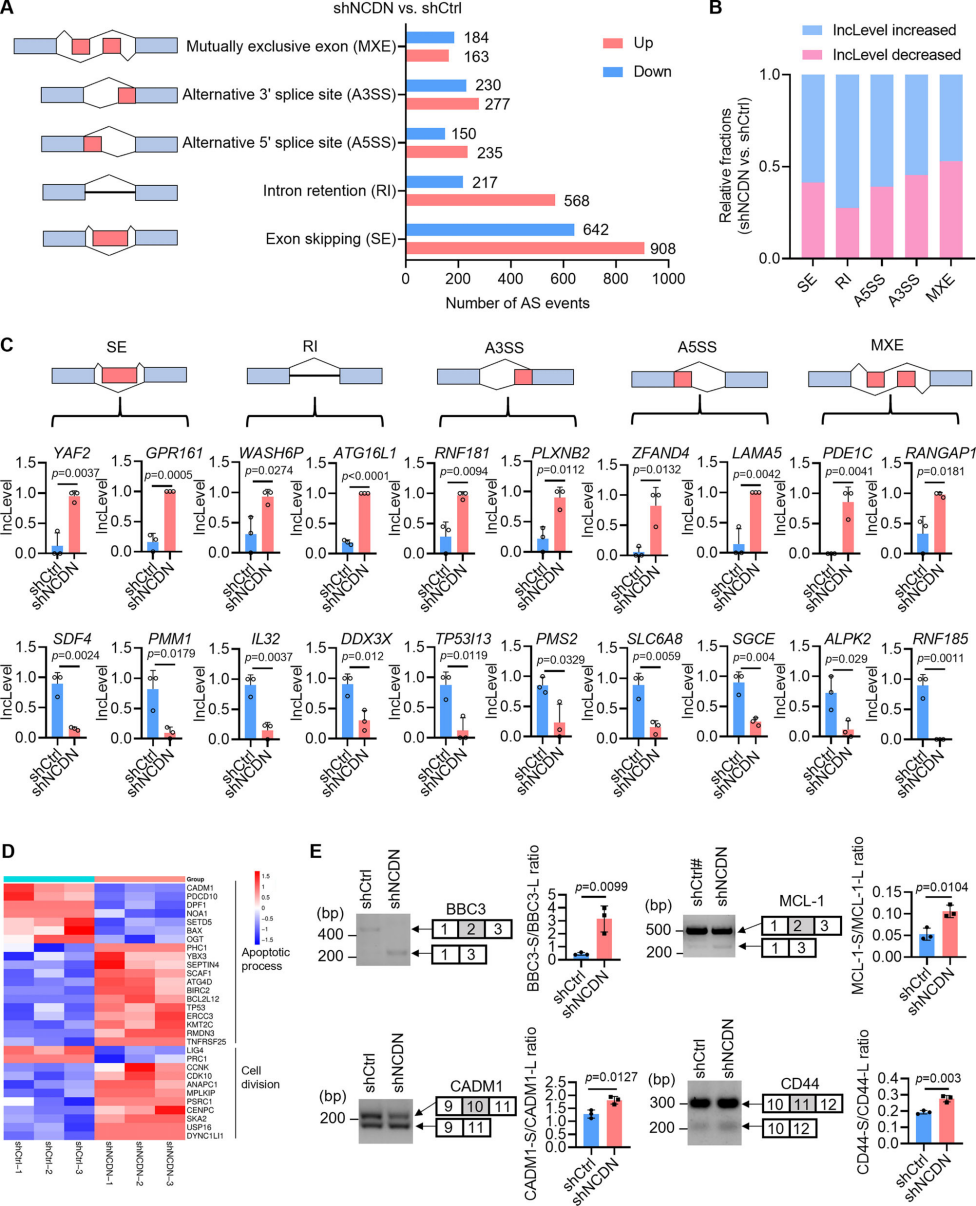
NCDN deficiency causes global alterations in alternative splicing patterns. (**A**) Quantification of AS event types significantly affected by NCDN knockdown in U251 cells, including skipped exon (SE), mutually exclusive exon (MXE), alternative 5′ splice site (A5SS), alternative 3′ splice site (A3SS), and retained intron (RI) events. (**B**) Proportions of AS events exhibiting increased and decreased inclusion levels (*Inclevel*) in NCDN-depleted versus control U251 cells. IncLevel represents the fraction of transcripts that include a specific alternative exon or splice site, with values ranging from 0 to 1. (**C**) Representative examples of different AS event types altered by NCDN deficiency in U251 cells. (**D**) Heatmap displaying *Inclevel* values of NCDN-regulated alternative splicing events in apoptotic process and cell division. (**E**) Reverse transcription PCR (RT-PCR) validation of selected skipped exon (SE) events in control and NCDN-depleted U251 cells. Fragment analysis of PCR products showing exon inclusion and exclusion isoforms is displayed on the right. RNA-seq data represent three independent biological replicates. Statistical analyses were performed using unpaired two-tailed Student’s *t*-test.

We identified thousands of NCDN-regulated AS events with an obvious change in inclusion levels (|∆IncLevel|>0.02, FDR ≤ 0.05) (Supplementary Data Sheet S2). We found that various types of AS events can be regulated by NCDN, including skipped exons (SE), retained introns (RI), alternative 5' splice site (A5SS), alternative 3' splice site (A3SS), and mutually exclusive exons (MXE) (Fig. 6A). As revealed by RNA-seq analysis, AS events exhibited increased inclusion levels upon NCDN depletion (Fig. 6B). Several specific splicing alterations were identified from the sequencing results (Fig. 6C). Consistent with previous results, we found that NCDN-regulated AS events were enriched in apoptotic process and cell division (Fig. 6D). SEs in representative apoptotic process genes in NCDN-deficient U251 cells were validated by RT-PCR (Fig. 6E). Collectively, these results suggest that NCDN contributes to the regulation of AS programs linked to cell survival.

**Figure 7.**
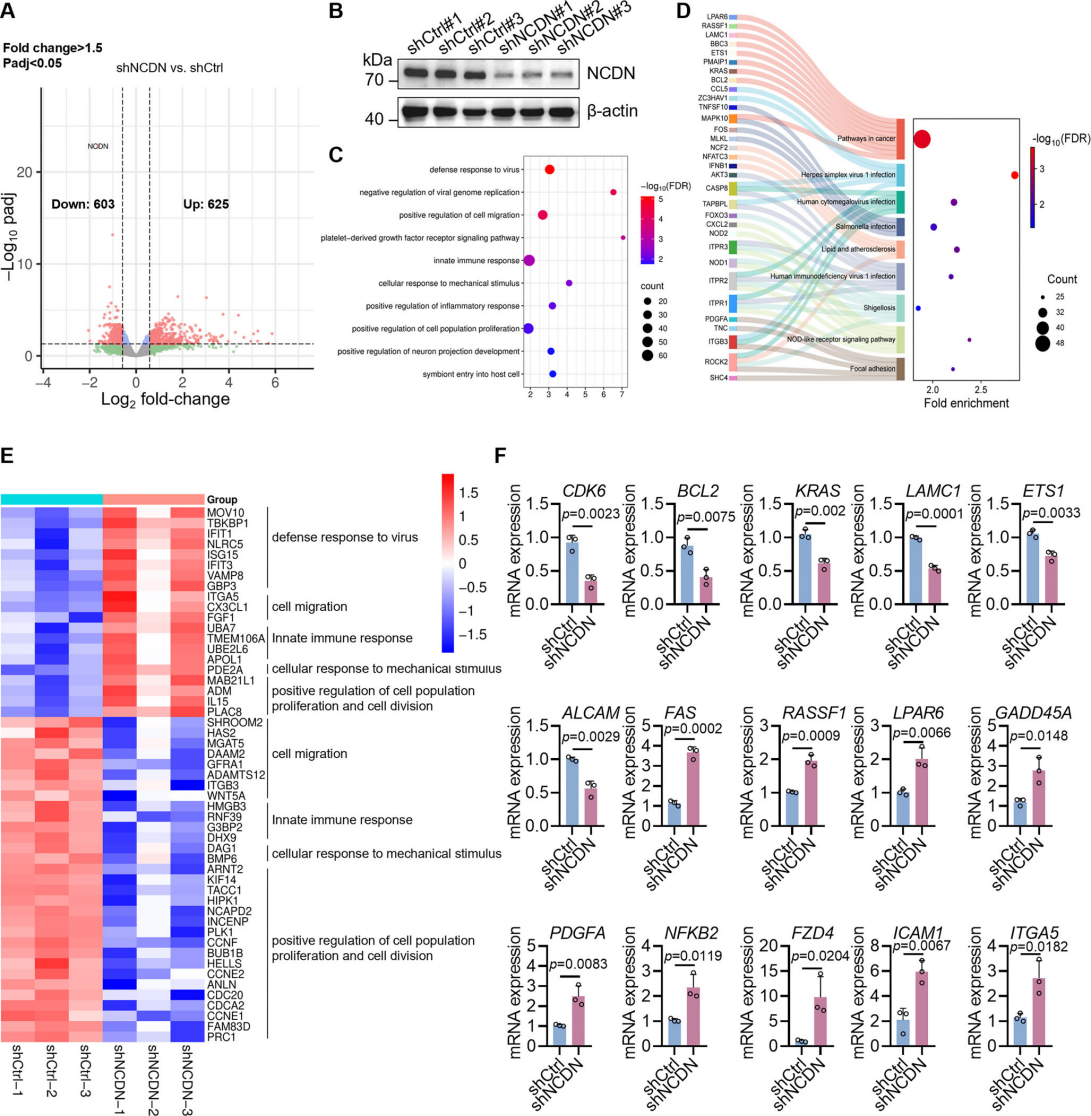
NCDN deficiency leads to transcriptome-wide changes in gene expression. (**A**) Volcano plot displaying DEGs in NCDN-depleted versus control U251 cells. Genes with a fold change > 1.5 and *Padj *< 0.05 were considered significantly upregulated or downregulated. Each dot represents an individual gene. (**B**) U251 cells were infected with lentivirus encoding control shRNA (shCtrl) or shRNA targeting NCDN (shNCDN). Seventy-two hours after infection, cells were either lysed for western blotting or harvested for RNA-seq analysis. (**C**) Gene Ontology (GO) enrichment analysis of biological process based on DEGs identified in NCDN-depleted U251 cells. Top 10 enriched GO terms are shown. (**D**) KEGG pathway enrichment analysis of DEGs upon NCDN knockdown. (**E**) Heatmap showing relative expression levels of selected upregulated and downregulated genes in NCDN-depleted U251 cells, clustered by enriched GO terms. Data are log_2_*X* transformed normalized FPKM. Color scale represents *Z*-scores of normalized expression values. (**F**) Independent RT-qPCR validation of representative genes involved in cancer-related pathways identified by RNA-seq analysis. Data represent three independent biological replicates. Statistical analysis was performed using unpaired two-tailed Student’s *t*-test.

Considering that alternative splicing can directly or indirectly affect gene expression levels, we further investigated the impact of NCDN deficiency on the transcriptome. Transcriptome profiling identified 625 upregulated genes and 603 downregulated genes (Fig. 7A and B, and Supplementary Data Sheet S3). DEG analysis highlighted enrichment in cancer-related pathways (Fig. 7C–E). Importantly, the differential expression of several candidates was validated by RT-qPCR (Fig. 7F). Taken together, these results demonstrate that NCDN loss causes both splicing aberrations and genome-wide changes in gene expression consistent with a more general role of NCDN in governing U5 snRNP biogenesis.

### Identification of glioblastoma prognosis-related AS events

**Figure 8.**
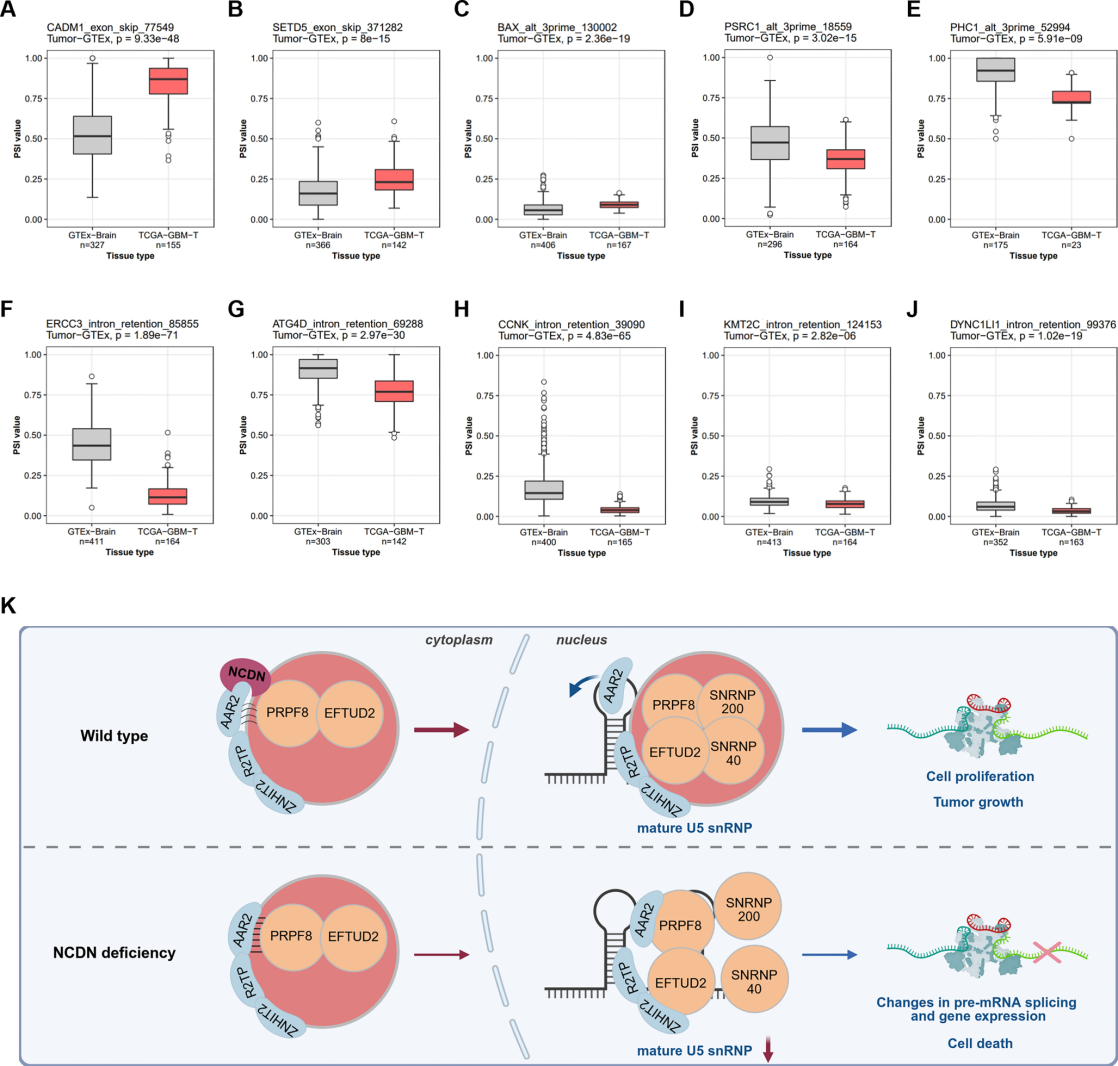
Identification of glioblastoma prognosis-related alternative splicing events and proposed model of NCDN in spliceosome biogenesis. (**A–J**) Percent splices-in (PSI) values of representative AS events in glioblastoma versus normal brain samples for the representative genes: (A) *CADM1*, (B) *SETD5*, (C) *BAX*, (D) *PSRC1*, (E) *PHC1*, (F) *ERCC3*, (G) *ATG4D*, (H) *CCNK*, (I) *KMT2C*, and (J) *DYNC1LI1*. Data were obtained from the TCGA SplAdder database. Each dot represents an individual patient sample. (**K**) Proposed model illustrating the role of NCDN in U5 snRNP biogenesis. In the presence of NCDN, PRPF8–AAR2 interactions are relatively loose, allowing efficient release of AAR2 and progression toward mature U5 snRNP formation. Loss of NCDN stabilizes the PRPF8–AAR2 intermediate in both the cytoplasm and nucleus, thereby impairing U5 snRNP maturation. The defect in U5 snRNP maturation cause widespread transcriptome-wide splicing dysregulation, contributing to reduced cell proliferation and increased apoptosis.

Aberrant spliceosome activity in cancer often gives rise to tumor-specific AS events that contribute to oncogenesis and disease progression [23, 43, 44]. To investigate whether NCDN-regulated AS events have clinical relevance in glioblastoma, we analyzed splicing patterns and associated clinical data from the TCGA SplAdder database. We focused on ten representative NCDN-regulated genes. We observed that the AS events for *CADM1, SETD5*, and *BAX* displayed a significant higher PSI value in cancer patients than in normal samples (Fig. 8A–C), while the PSI values were significantly lower for *PSRC1, PHC1, ERCC3, ATG4D, CCNK, KMT2C*, and *DYNC1LI1* in patients with glioblastoma (Fig. 8D–J). These results suggest that NCDN-regulated AS events are not only functionally relevant in glioma biology but may also serve as potential prognostic biomarkers for glioblastoma. The tumor-specific splicing signatures defined here highlight the broader clinical significance of NCDN-mediated spliceosome regulation.

## Discussion

The spliceosome is an exceptionally efficient and accurate molecular machine that is essential for pre-mRNA processing. To produce functional spliceosomes, individual snRNPs require sequential, compartment-specific assembly, coordinated both temporally and spatially [4]. U5 snRNP biogenesis, in particular, represents a complex multistep process that depends on several assembly chaperones, including the R2TP/PFDL complex, ZNHIT2 and ECD [14, 16, 18]. Disruption of splicing factors often leads to defective spliceosome assembly, resulting in widespread alternative splicing alterations and pathological consequences, including cancer [45, 46].

To date, studies of NCDN have primarily centered on its roles in neuronal development. However, NCDN’s interactome hints at a previously unappreciated function in spliceosome assembly. Here, we uncover a role for NCDN in the biogenesis and assembly of the U5 snRNP complex and demonstrate that dysregulation of NCDN perturbs spliceosome function, leading to defects in pre-mRNA splicing and widespread gene expression changes, thereby affecting tumor progression.

Our proteomic and biochemical data establish NCDN as a U5 snRNP-associated protein. NCDN’s interaction with the U5 core proteins PRPF8 and EFTUD2, as well as with the dedicated chaperone AAR2 and R2TP/PFDL complex, positions it alongside known assembly factors such as ZNHIT2. However, unlike ZNHIT2, which primarily functions as a linker between U5 components and chaperone complexes, our data suggest that NCDN contributes to the proper progression of the PRPF8–AAR2 assembly intermediate. NCDN directly associates with AAR2, and loss of NCDN strengthens the interaction between PRPF8 and AAR2, leading to accumulation of an immature U5 assembly state. Finally, depletion of NCDN reduces the levels of mature U5 snRNP, consequently impairing the assembly of the U4/U6·U5 tri-snRNP.

Dysregulation of the spliceosome leads to aberrant splicing of downstream targets, many of which contribute to oncogenesis [47]. Recurrent mutations in splicing factors such as SF3B1, U2AF1, and SRSF2 cause splicing dysregulation that drives cancer progression [48–50]. Our data show that NCDN deficiency inhibits tumor cell growth and promotes apoptosis, whereas NCDN overexpression promotes tumor cell growth. Consistent with this result, high NCDN expression is closely associated with poor prognosis of glioma patients. NCDN loss leads to widespread alterations in alternative splicing and gene expression, including changes in cancer-related pathway. These dysregulated events likely contribute to tumor cell survival, offering mechanistic insight into how aberrant spliceosome activity can support oncogenesis. Indeed, downregulation of many components involved in snRNP biogenesis or spliceosome function would be expected to produce similar phenotypes.

Based on our data, we propose a model in which NCDN associates with PRPF8–EFTUD2–AAR2 complexes in the cytoplasm and regulates the progression of this assembly intermediate toward mature U5 snRNP formation. In the absence of NCDN, PRPF8–AAR2 complexes become aberrantly stabilized, leading to reduced U5 availability for spliceosome formation and widespread splicing defects. Notably, the effects of NCDN on AAR2 dissociation are relatively limited, suggesting that additional mechanisms, such as phosphorylation of AAR2 [12, 51] and the coordinated action of other assembly factors and chaperones, also contribute to U5 snRNP maturation [14, 18]. Several limitations of this study remain. In particular, structural and kinetic characterization of NCDN–AAR2–PRPF8 complexes will be essential to define the precise molecular mechanism by which NCDN promotes U5 snRNP assembly progression. Given its oncogenic role, targeting NCDN may represent a novel therapeutic strategy in spliceosome‐addicted cancers. Small molecules that disrupt NCDN function could restore normal splicing patterns and sensitize tumor cells to apoptosis.

In summary, our work identifies NCDN as a regulator of U5 snRNP biogenesis that contributes to efficient spliceosome assembly and cancer cell survival. These findings expand the repertoire of spliceosome assembly factors and underscore the importance of regulated snRNP maturation in tumor biology, highlighting spliceosome modulation as a promising avenue for therapeutic intervention.

## Supplementary Material

gkag685_Supplemental_Files

## Data Availability

The RNA-seq data presented in this study have been deposited in the GEO repository (https://www.ncbi.nlm.nih.gov/geo/), accession number GSE299993. The mass spectrometry proteomics data have been deposited to the ProteomeXchange Consortium via the PRIDE partner repository (https://www.ebi.ac.uk/pride/) with the dataset identifier PXD068384. All the other data supporting the findings of the paper are presented in the manuscript and supplementary materials.
