# Supplementary material for: Neurochondrin promotes U5 snRNP maturation by regulating AAR2 release from PRPF8
Source: Nucleic Acids Res. 2026 Jul 7;54(13):gkag685. doi: 10.1093/nar/gkag685 (PMC13338715; doi:10.1093/nar/gkag685)
Supplement: gkag685_Supplemental_Files [file gkag685_supplemental_files.zip › Supplementary Data Sheet S1. Identified NCDN-interacting proteins.pdf]

Supplementary Dataset S1. NCDN-interacting proteins identified by co-immunoprecipitation coupled with mass spectrometry analysis

Accession	Description	Exp. q-value: Combined	Sum PEP Score	Coverage [%]	# Peptides	# PSMs	# Unique Peptides	# AAs	MW [kDa]	calc. pI	Score Sequest HT: Sequest HT	# Peptides (by Search Engine): Sequest HT	# Razor Peptides	Abundances (Grouped): NCDN	Abundances (Grouped): Vector	Ratio(NCDN/Ve
P62736	Actin, aortic smooth muscle OS=Homo sapiens OX=9606 GN=ACTA2 PE=1 SV=1	0	92.969	27	11	176	2	377	42	5.39	361.01	11	0	4064071.25		NCDN only
P78386	Keratin, type II cuticular Hb5 OS=Homo sapiens OX=9606 GN=KRT85 PE=1 SV=1	0	71.556	20	14	139	5	507	55.8	6.55	203.19	14	1	41885181.5		NCDN only
Q13509	Tubulin beta-3 chain OS=Homo sapiens OX=9606 GN=TUBB3 PE=1 SV=2	0	51.169	20	8	45	1	450	50.4	4.93	80.87	8	0	134941.3789		NCDN only
P35232	Prohibitin OS=Homo sapiens OX=9606 GN=PHB PE=1 SV=1	0	25.425	22	7	35	7	272	29.8	5.76	58.79	7	0	14200569.91		NCDN only
Q9NX63	MICOS complex subunit MIC19 OS=Homo sapiens OX=9606 GN=CHCHD3 PE=1 SV=1	0	12.072	15	4	34	4	227	26.1	8.28	64.49	4	0	15691354.3		NCDN only
Q7Z434	Mitochondrial antiviral-signaling protein OS=Homo sapiens OX=9606 GN=MAVS PE=1 SV=2	0	23.821	8	4	31	4	540	56.5	5.52	73.28	4	0	5124184.734		NCDN only
Q9Y3B4	Splicing factor 3B subunit 6 OS=Homo sapiens OX=9606 GN=SF3B6 PE=1 SV=1	0.002	2.558	6	1	26	1	125	14.6	9.38	37.66	1	0	680951.5313		NCDN only
Q05639	Elongation factor 1-alpha 2 OS=Homo sapiens OX=9606 GN=EEF1A2 PE=1 SV=1	0	19.707	11	6	25	1	463	50.4	9.03	52.4	6	0	28476.62695		NCDN only
Q5BKT4	Dol-P-Glc:Glc(2)Man(9)GlcNAc(2)-PP-Dol alpha-1,2-glucosyltransferase OS=Homo sapiens OX=9606 GN=ALG10 PE=1 SV=1	0.002	2.308	1	1	25	1	473	55.6	9.29	39.77	1	0	1547834.789		NCDN only
P61981	14-3-3 protein gamma OS=Homo sapiens OX=9606 GN=YWHAG PE=1 SV=2	0	8.954	13	4	25	2	247	28.3	4.89	40.63	4	0	147827.5625		NCDN only
P51572	B-cell receptor-associated protein 31 OS=Homo sapiens OX=9606 GN=BCAP31 PE=1 SV=3	0	49.809	27	11	25	11	246	28	8.44	44.91	11	0	17199894.77		NCDN only
P11021	Endoplasmic reticulum chaperone BiP OS=Homo sapiens OX=9606 GN=HSPA5 PE=1 SV=2	0	20.628	11	7	24	6	654	72.3	5.16	46.88	7	0	13673954.64		NCDN only
Q6NTF9	Rhomboid domain-containing protein 2 OS=Homo sapiens OX=9606 GN=RHBDD2 PE=1 SV=2	0	6.201	5	2	23	2	364	39.2	9.32	28.01	2	0	3257830.578		NCDN only
P27348	14-3-3 protein theta OS=Homo sapiens OX=9606 GN=YWHAQ PE=1 SV=1	0	12.873	12	3	23	1	245	27.7	4.78	43.04	3	0	1701953.75		NCDN only
P53007	Tricarboxylate transport protein, mitochondrial OS=Homo sapiens OX=9606 GN=SLC25A1 PE=1 SV=2	0	8.464	8	3	22	3	311	34	9.89	52.65	3	0	4919394.281		NCDN only
Q9UNF1	Melanoma-associated antigen D2 OS=Homo sapiens OX=9606 GN=MAGED2 PE=1 SV=2	0	11.959	6	4	21	4	606	64.9	9.32	52.51	4	0	1374153.793		NCDN only
Q96I24	Far upstream element-binding protein 3 OS=Homo sapiens OX=9606 GN=FUBP3 PE=1 SV=2	0	8.808	5	3	20	2	572	61.6	8.38	30.13	3	0	177807.1523		NCDN only
P43304	Glycerol-3-phosphate dehydrogenase, mitochondrial OS=Homo sapiens OX=9606 GN=GPD2 PE=1 SV=3	0	5.44	2	2	19	2	727	80.8	7.69	27.2	2	0	136360.793		NCDN only
Q8NBM4	Ubiquitin-associated domain-containing protein 2 OS=Homo sapiens OX=9606 GN=UBAC2 PE=1 SV=1	0	5.813	5	2	19	2	344	38.9	9.01	41.96	2	0	1011965.734		NCDN only
P51148	Ras-related protein Rab-5C OS=Homo sapiens OX=9606 GN=RAB5C PE=1 SV=2	0	6.299	6	1	19	1	216	23.5	8.41	35.4	1	0	600187.9688		NCDN only
Q6Y1H2	Very-long-chain (3R)-3-hydroxyacyl-CoA dehydratase 2 OS=Homo sapiens OX=9606 GN=HACD2 PE=1 SV=1	0	6.672	8	2	19	2	254	28.4	9.55	27.48	2	0	21487.19727		NCDN only
Q8TB61	Adenosine 3'-phospho 5'-phosphosulfate transporter 1 OS=Homo sapiens OX=9606 GN=SLC35B2 PE=1 SV=1	0	20.36	12	5	19	5	432	47.5	9.16	34.89	5	0	10607061.55		NCDN only
Q9BVC6	Transmembrane protein 109 OS=Homo sapiens OX=9606 GN=TMEM109 PE=1 SV=1	0	15.999	13	4	19	4	243	26.2	10.48	40.35	4	0	43785705.99		NCDN only
Q9Y312	Protein AAR2 homolog OS=Homo sapiens OX=9606 GN=AAR2 PE=1 SV=2	0	24.74	12	4	18	4	384	43.4	5.96	32.53	4	0	7759358.156		NCDN only
O60725	Protein-S-isoprenylcysteine O-methyltransferase OS=Homo sapiens OX=9606 GN=ICMT PE=1 SV=1	0.002	2.384	3	1	17	1	284	31.9	7.96	12.24	1	0	2390185.203		NCDN only
P16615	Sarcoplasmic/endoplasmic reticulum calcium ATPase 2 OS=Homo sapiens OX=9606 GN=ATP2A2 PE=1 SV=1	0	23.891	7	7	17	7	1042	114.7	5.34	17.53	7	0	3486229.561		NCDN only
Q9P270	SLAIN motif-containing protein 2 OS=Homo sapiens OX=9606 GN=SLAIN2 PE=1 SV=2	0	8.329	3	2	16	2	581	62.5	9.45	27.69	2	0	103295.2188		NCDN only
Q9H3K2	Growth hormone-inducible transmembrane protein OS=Homo sapiens OX=9606 GN=GHITM PE=1 SV=2	0	23.392	9	3	16	3	345	37.2	9.94	43.58	3	0	12096468.2		NCDN only
O95905	Protein ecdysoneless homolog OS=Homo sapiens OX=9606 GN=ECD PE=1 SV=1	0	12.805	7	6	15	6	644	72.7	4.87	22.42	6	0	19592196.32		NCDN only
P08962	CD63 antigen OS=Homo sapiens OX=9606 GN=CD63 PE=1 SV=2	0	6.287	6	2	15	2	238	25.6	7.81	17.68	2	0	2071105.32		NCDN only
P42167	Lamina-associated polypeptide 2, isoforms beta/gamma OS=Homo sapiens OX=9606 GN=TMPO PE=1 SV=2	0	11.684	6	3	15	3	454	50.6	9.38	24.16	3	0	7777697.326		NCDN only
Q9UGP8	Translocation protein SEC63 homolog OS=Homo sapiens OX=9606 GN=SEC63 PE=1 SV=2	0.002	2.698	1	1	15	1	760	87.9	5.31	27.6	1	0	996157.0938		NCDN only
P46977	Dolichyl-diphosphooligosaccharide--protein glycosyltransferase subunit STT3A OS=Homo sapiens OX=9606 GN=STT3A PE=1 SV=2	0	13.872	4	3	14	3	705	80.5	8.07	14.6	3	0	611171.9375		NCDN only
O75431	Metaxin-2 OS=Homo sapiens OX=9606 GN=MTX2 PE=1 SV=1	0	11.422	4	1	14	1	263	29.7	6.29	25.32	1	0	1055583.133		NCDN only
Q8N1F7	Nuclear pore complex protein Nup93 OS=Homo sapiens OX=9606 GN=NUP93 PE=1 SV=2	0	6.904	3	3	14	3	819	93.4	5.72	21.91	3	0	533281.8906		NCDN only
Q9NXS2	Glutaminyl-peptide cyclotransferase-like protein OS=Homo sapiens OX=9606 GN=QPCTL PE=1 SV=2	0	14.764	10	4	13	4	382	42.9	9.82	23.81	4	0	28603893		NCDN only
Q9Y5M8	Signal recognition particle receptor subunit beta OS=Homo sapiens OX=9606 GN=SRPRB PE=1 SV=3	0.002	3.156	3	1	13	1	271	29.7	9.04	13.49	1	0	1183566.563		NCDN only
P26599	Polypyrimidine tract-binding protein 1 OS=Homo sapiens OX=9606 GN=PTBP1 PE=1 SV=1	0	5.643	4	2	13	2	531	57.2	9.17	13.73	2	0	232605.7695		NCDN only
P60468	Protein transport protein Sec61 subunit beta OS=Homo sapiens OX=9606 GN=SEC61B PE=1 SV=2	0	10.211	28	2	13	2	96	10	11.56	23.05	2	0	1116062.336		NCDN only
P12268	Inosine-5'-monophosphate dehydrogenase 2 OS=Homo sapiens OX=9606 GN=IMPDH2 PE=1 SV=2	0	19.206	8	4	13	2	514	55.8	6.9	19.13	4	1	1015143.109		NCDN only
Q7L8L6	FAST kinase domain-containing protein 5, mitochondrial OS=Homo sapiens OX=9606 GN=FASTKD5 PE=1 SV=1	0	4.405	1	1	12	1	764	86.5	8.13	14.75	1	0	372912.2344		NCDN only
P42704	Leucine-rich PPR motif-containing protein, mitochondrial OS=Homo sapiens OX=9606 GN=LRPPRC PE=1 SV=3	0	11.375	2	3	12	3	1394	157.8	6.13	23.55	3	0	672767.7324		NCDN only
P11717	Cation-independent mannose-6-phosphate receptor OS=Homo sapiens OX=9606 GN=IGF2R PE=1 SV=3	0	12.536	2	5	12	5	2491	274.2	5.94	10.39	5	0	169122.8398		NCDN only
P14209	CD99 antigen OS=Homo sapiens OX=9606 GN=CD99 PE=1 SV=1	0	4.613	5	1	12	1	185	18.8	4.75	20.87	1	0	303854.6406		NCDN only
Q9P035	Very-long-chain (3R)-3-hydroxyacyl-CoA dehydratase 3 OS=Homo sapiens OX=9606 GN=HACD3 PE=1 SV=2	0	21.774	7	4	11	4	362	43.1	8.94	28.84	4	0	17657735.41		NCDN only
Q5RI15	Cytochrome c oxidase assembly protein COX20, mitochondrial OS=Homo sapiens OX=9606 GN=COX20 PE=1 SV=2	0	4.371	11	1	11	1	118	13.3	8.76	15.01	1	0	637143.3047		NCDN only
A0FGR8	Extended synaptotagmin-2 OS=Homo sapiens OX=9606 GN=ESYT2 PE=1 SV=1	0	18.017	6	5	11	5	921	102.3	9.26	20.58	5	0	17909777.16		NCDN only
Q86Y82	Syntaxin-12 OS=Homo sapiens OX=9606 GN=STX12 PE=1 SV=1	0	8.471	9	3	10	3	276	31.6	5.59	12.57	3	0	1077601.914		NCDN only
P54886	Delta-1-pyrroline-5-carboxylate synthase OS=Homo sapiens OX=9606 GN=ALDH18A1 PE=1 SV=2	0	10.443	3	3	10	3	795	87.2	7.12	20.09	3	0	898403.7969		NCDN only
Q9BVV7	Mitochondrial import inner membrane translocase subunit Tim21 OS=Homo sapiens OX=9606 GN=TIMM21 PE=1 SV=1	0	8.942	10	3	10	3	248	28.2	9.7	13.84	3	0	1022699.578		NCDN only
P23588	Eukaryotic translation initiation factor 4B OS=Homo sapiens OX=9606 GN=EIF4B PE=1 SV=2	0	5.609	2	1	10	1	611	69.1	5.73	14.64	1	0	157792.668		NCDN only
Q9Y2W6	Tudor and KH domain-containing protein OS=Homo sapiens OX=9606 GN=TDRKH PE=1 SV=2	0	10.76	3	2	10	2	561	62	5.02	17.68	2	0	1810008.297		NCDN only
O14654	Insulin receptor substrate 4 OS=Homo sapiens OX=9606 GN=IRS4 PE=1 SV=1	0	13.382	3	3	10	3	1257	133.7	8.44	11.39	3	0	399191.7754		NCDN only
O15269	Serine palmitoyltransferase 1 OS=Homo sapiens OX=9606 GN=SPTLC1 PE=1 SV=1	0	9.341	5	3	10	3	473	52.7	6.01	10.75	3	0	354155.3906		NCDN only
P35542	Serum amyloid A-4 protein OS=Homo sapiens OX=9606 GN=SAA4 PE=1 SV=2	0.002	2.212	6	1	10	1	130	14.7	9.07	10.53	1	0	135407.8125		NCDN only
Q9UM00	Calcium load-activated calcium channel OS=Homo sapiens OX=9606 GN=TMCO1 PE=1 SV=2	0	14.896	13	3	9	3	239	27.1	10.26	21.94	3	0	27332313.63		NCDN only
Q96K37	Solute carrier family 35 member E1 OS=Homo sapiens OX=9606 GN=SLC35E1 PE=1 SV=2	0	21.337	8	2	9	2	410	44.7	9.79	19.73	2	0	1323387.75		NCDN only
Q12931	Heat shock protein 75 kDa, mitochondrial OS=Homo sapiens OX=9606 GN=TRAP1 PE=1 SV=3	0	16.01	8	5	9	5	704	80.1	8.21	10.56	5	0	3072090.789		NCDN only
Q8WUY1	Protein THEM6 OS=Homo sapiens OX=9606 GN=THEM6 PE=1 SV=2	0	18.083	10	3	9	3	208	23.9	9.55	19.96	3	0	4185909.602		NCDN only
P10321	HLA class I histocompatibility antigen, C alpha chain OS=Homo sapiens OX=9606 GN=HLA-C PE=1 SV=3	0	3.881	2	1	9	1	366	40.6	6.04	17.14	1	0	1836969.563		NCDN only
P56192	Methionine--tRNA ligase, cytoplasmic OS=Homo sapiens OX=9606 GN=MARS1 PE=1 SV=2	0	6.159	3	3	8	3	900	101.1	6.16	8.64	3	0	303766.7266		NCDN only
P49327	Fatty acid synthase OS=Homo sapiens OX=9606 GN=FASN PE=1 SV=3	0	8.649	1	3	8	3	2511	273.3	6.44	13.51	3	0	359337.8281		NCDN only
Q969E2	Secretory carrier-associated membrane protein 4 OS=Homo sapiens OX=9606 GN=SCAMP4 PE=1 SV=1	0	9.026	16	2	8	2	229	25.7	8.82	19.43	2	0	2653388.188		NCDN only
Q9UBI6	Guanine nucleotide-binding protein G(I)/G(S)/G(O) subunit gamma-12 OS=Homo sapiens OX=9606 GN=GNG12 PE=1 SV=3	0	4.809	15	1	8	1	72	8	8.97	16.98	1	0	76106		NCDN only
Q9BV68	E3 ubiquitin-protein ligase RNF126 OS=Homo sapiens OX=9606 GN=RNF126 PE=1 SV=2	0.002	2.872	3	1	8	1	311	33.8	5.47	18	1	0	53738.26172		NCDN only
P30153	Serine/threonine-protein phosphatase 2A 65 kDa regulatory subunit A alpha isoform OS=Homo sapiens OX=9606 GN=PPP2R1A PE=1 SV=4	0	11.599	6	4	8	3	589	65.3	5.11	11.63	4	1	1679101.391		NCDN only
Q9NZJ7	Mitochondrial carrier homolog 1 OS=Homo sapiens OX=9606 GN=MTCH1 PE=1 SV=1	0	3.922	3	1	8	1	389	41.5	9.32	14.25	1	0	380662.8516		NCDN only
Q6UB35	Monofunctional C1-tetrahydrofolate synthase, mitochondrial OS=Homo sapiens OX=9606 GN=MTHFD1L PE=1 SV=1	0	5.914	1	1	8	1	978	105.7	8.06	6.21	1	0	129444.207		NCDN only
O00264	Membrane-associated progesterone receptor component 1 OS=Homo sapiens OX=9606 GN=PGRMC1 PE=1 SV=3	0	22.614	20	3	8	3	195	21.7	4.7	23.18	3	0	11848893.59		NCDN only
Q9P258	Protein RCC2 OS=Homo sapiens OX=9606 GN=RCC2 PE=1 SV=2	0	6.634	3	2	8	2	522	56	8.78	10.04	2	0	49394.46875		NCDN only
Q6YN16	Hydroxysteroid dehydrogenase-like protein 2 OS=Homo sapiens OX=9606 GN=HSDL2 PE=1 SV=1	0	7.24	6	3	8	3	418	45.4	7.99	5.12	3	0	1015673.828		NCDN only
P23526	Adenosylhomocysteinase OS=Homo sapiens OX=9606 GN=AHCY PE=1 SV=4	0	7.013	4	2	8	2	432	47.7	6.34	13.56	2	0	660369.9414		NCDN only
O15228	Dihydroxyacetone phosphate acyltransferase OS=Homo sapiens OX=9606 GN=GNPAT PE=1 SV=1	0	10.362	5	4	8	4	680	77.1	6.57	15.45	4	0	767952.2725		NCDN only
O95801	Tetratricopeptide repeat protein 4 OS=Homo sapiens OX=9606 GN=TTC4 PE=1 SV=3	0	6.994	7	3	8	3	387	44.7	5.6	15.43	3	0	2084437.563		NCDN only
Q969Z3	Mitochondrial amidoxime reducing component 2 OS=Homo sapiens OX=9606 GN=MTARC2 PE=1 SV=1	0	4.764	5	2	8	2	335	38	9.16	13.98	2	0	209234.5156		NCDN only
P10586	Receptor-type tyrosine-protein phosphatase F OS=Homo sapiens OX=9606 GN=PTPRF PE=1 SV=2	0	7.541	2	3	8	3	1907	212.7	6.3	11.9	3	0	4538328.697		NCDN only
Q9NXE4	Sphingomyelin phosphodiesterase 4 OS=Homo sapiens OX=9606 GN=SMPD4 PE=1 SV=3	0	6.672	4	4	7	4	866	97.7	8.27	5.51	4	0	228492.4805		NCDN only
P18031	Tyrosine-protein phosphatase non-receptor type 1 OS=Homo sapiens OX=9606 GN=PTPN1 PE=1 SV=1	0.002	3.134	2	1	7	1	435	49.9	6.27	13.35	1	0	398147.0723		NCDN only
Q96TC7	Regulator of microtubule dynamics protein 3 OS=Homo sapiens OX=9606 GN=RMDN3 PE=1 SV=2	0	14.636	10	4	7	4	470	52.1	5.1	15.39	4	0	2943814.938		NCDN only
Q8NBI6	Xyloside xylosyltransferase 1 OS=Homo sapiens OX=9606 GN=XXYLT1 PE=1 SV=1	0	4.089	3	1	7	1	393	43.8	8.13	15.4	1	0	246260.75		NCDN only
Q96LL9	DnaJ homolog subfamily C member 30, mitochondrial OS=Homo sapiens OX=9606 GN=DNAJC30 PE=1 SV=3	0.002	2.244	4	1	7	1	226	25.9	10.49	13.31	1	0	263752.5625		NCDN only
Q8N4H5	Mitochondrial import receptor subunit TOM5 homolog OS=Homo sapiens OX=9606 GN=TOMM5 PE=1 SV=1	0	6.386	41	3	7	3	51	6	9.7	7.07	3	0	5027994.697		NCDN only
P06493	Cyclin-dependent kinase 1 OS=Homo sapiens OX=9606 GN=CDK1 PE=1 SV=3	0	3.713	3	1	7	1	297	34.1	8.4	5.32	1	0	131712.5117		NCDN only
P25205	DNA replication licensing factor MCM3 OS=Homo sapiens OX=9606 GN=MCM3 PE=1 SV=3	0	12.26	5	4	7	4	808	90.9	5.77	14.56	4	0	859145.4297		NCDN only
Q6IAN0	Dehydrogenase/reductase SDR family member 7B OS=Homo sapiens OX=9606 GN=DHRS7B PE=1 SV=2	0	19.585	9	3	7	3	325	35.1	9.55	21.3	3	0	3873146.078		NCDN only
P49902	Cytosolic purine 5'-nucleotidase OS=Homo sapiens OX=9606 GN=NT5C2 PE=1 SV=1	0.001	3.271	2	2	7	2	561	64.9	6.14	3.37	2	0	155699.6055		NCDN only
P62873	Guanine nucleotide-binding protein G(I)/G(S)/G(T) subunit beta-1 OS=Homo sapiens OX=9606 GN=GNB1 PE=1 SV=3	0	8.011	6	2	7	1	340	37.4	6	11.7	2	0	111542.2734		NCDN only
Q7KZN9	Cytochrome c oxidase assembly protein COX15 homolog OS=Homo sapiens OX=9606 GN=COX15 PE=1 SV=1	0	3.94	4	2	7	2	410	46	9.82	9.43	2	0	2841077.992		NCDN only
Q9Y4R8	Telomere length regulation protein TEL2 homolog OS=Homo sapiens OX=9606 GN=TELO2 PE=1 SV=2	0.002	2.703	1	1	7	1	837	91.7	5.76	5.35	1	0	61862.33203		NCDN only
Q9HCC0	Methylcrotonoyl-CoA carboxylase beta chain, mitochondrial OS=Homo sapiens OX=9606 GN=MCCC2 PE=1 SV=1	0	4.404	3	2	7	2	563	61.3	7.68	5.27	2	0	28940.60547		NCDN only
Q86X52	Chondroitin sulfate synthase 1 OS=Homo sapiens OX=9606 GN=CHSY1 PE=1 SV=3	0	6.121	2	2	7	2	802	91.7	9.23	10.23	2	0	151287.0996		NCDN only
Q6ZXV5	Protein O-mannosyl-transferase TMTC3 OS=Homo sapiens OX=9606 GN=TMTC3 PE=1 SV=2	0	5.526	2	3	7	3	915	103.9	8.87	8.66	3	0	974320.2148		NCDN only
Q15005	Signal peptidase complex subunit 2 OS=Homo sapiens OX=9606 GN=SPCS2 PE=1 SV=3	0	4.1	4	1	7	1	226	25	8.47	13.13	1	0	46133.47656		NCDN only
Q5T6F0	DDB1- and CUL4-associated factor 12 OS=Homo sapiens OX=9606 GN=DCAF12 PE=1 SV=1	0	7.606	5	3	7	3	453	50.5	9.1	8.81	3	0	722773.207		NCDN only
P20930	Filaggrin OS=Homo sapiens OX=9606 GN=FLG PE=1 SV=3	0	7.897	1	4	7	4	4061	434.9	9.25	7.45	4	0	51146.12109		NCDN only
Q9H9B4	Sideroflexin-1 OS=Homo sapiens OX=9606 GN=SFXN1 PE=1 SV=4	0	5.7	7	2	7	2	322	35.6	9.07	9.88	2	0	264831.3906		NCDN only
O60884	DnaJ homolog subfamily A member 2 OS=Homo sapiens OX=9606 GN=DNAJA2 PE=1 SV=1	0	4.124	2	1	7	1	412	45.7	6.48	8.48	1	0	912114.0938		NCDN only
Q9P2N5	RNA-binding protein 27 OS=Homo sapiens OX=9606 GN=RBM27 PE=1 SV=2	0.002	2.716	1	1	7	1	1060	118.6	9.19	0	1	0	43313.46094		NCDN only
Q9UBM7	7-dehydrocholesterol reductase OS=Homo sapiens OX=9606 GN=DHCR7 PE=1 SV=1	0	5.087	4	2	7	2	475	54.5	8.7	8.21	2	0	868631.5625		NCDN only
Q9Y5Z9	UbiA prenyltransferase domain-containing protein 1 OS=Homo sapiens OX=9606 GN=UBIAD1 PE=1 SV=1	0	5.182	3	1	7	1	338	36.8	8.15	18.1	1	0	1562208.281		NCDN only
Q9UHQ4	B-cell receptor-associated protein 29 OS=Homo sapiens OX=9606 GN=BCAP29 PE=1 SV=2	0	16.723	10	3	7	3	241	28.3	9.54	11.07	3	0	230139.0156		NCDN only
P32189	Glycerol kinase OS=Homo sapiens OX=9606 GN=GK PE=1 SV=3	0	6.338	3	2	7	2	559	61.2	6.54	16.66	2	0	1383937.438		NCDN only
P62820	Ras-related protein Rab-1A OS=Homo sapiens OX=9606 GN=RAB1A PE=1 SV=3	0	7.142	13	3	6	3	205	22.7	6.21	1.82	3	0	131464.9727		NCDN only
P13535	Myosin-8 OS=Homo sapiens OX=9606 GN=MYH8 PE=2 SV=3	0	7.586	1	3	6	1	1937	222.6	5.74	5.57	3	0	1413235.219		NCDN only
O75695	Protein XRP2 OS=Homo sapiens OX=9606 GN=RP2 PE=1 SV=4	0	3.761	4	2	6	2	350	39.6	5.12	3.6	2	0	231846.7031		NCDN only
P17152	Transmembrane protein 11, mitochondrial OS=Homo sapiens OX=9606 GN=TMEM11 PE=1 SV=1	0.001	3.331	6	1	6	1	192	21.5	7.36	13.16	1	0	495425.9531		NCDN only
Q6P3W7	SCY1-like protein 2 OS=Homo sapiens OX=9606 GN=SCYL2 PE=1 SV=1	0	5.691	3	3	6	3	929	103.6	8.22	10.77	3	0	196465.6406		NCDN only
Q99615	DnaJ homolog subfamily C member 7 OS=Homo sapiens OX=9606 GN=DNAJC7 PE=1 SV=2	0	10.782	5	3	6	3	494	56.4	6.96	11.3	3	0	359934.9063		NCDN only
O75534	Cold shock domain-containing protein E1 OS=Homo sapiens OX=9606 GN=CSDE1 PE=1 SV=2	0	9.695	4	3	6	3	798	88.8	6.25	12.55	3	0	1403660.609		NCDN only
Q96HV5	Transmembrane protein 41A OS=Homo sapiens OX=9606 GN=TMEM41A PE=1 SV=1	0	3.711	4	1	6	1	264	29.6	9.03	17.18	1	0	2687522.5		NCDN only
Q969V5	Mitochondrial ubiquitin ligase activator of NFKB 1 OS=Homo sapiens OX=9606 GN=MUL1 PE=1 SV=1	0	17.152	10	3	6	3	352	39.8	8.13	10.1	3	0	3301901.625		NCDN only
Q9C0C4	Semaphorin-4C OS=Homo sapiens OX=9606 GN=SEMA4C PE=1 SV=2	0	5.59	3	3	6	3	833	92.6	7.28	9.34	3	0	969177.0781		NCDN only
Q9Y512	Sorting and assembly machinery component 50 homolog OS=Homo sapiens OX=9606 GN=SAMM50 PE=1 SV=3	0	3.809	3	2	6	2	469	51.9	6.9	9.38	2	0	1246717.477		NCDN only
Q7Z2K6	Endoplasmic reticulum metallopeptidase 1 OS=Homo sapiens OX=9606 GN=ERMP1 PE=1 SV=2	0	6.531	2	2	6	2	904	100.2	7.52	7.34	2	0	138406.2461		NCDN only
P31040	Succinate dehydrogenase [ubiquinone] flavoprotein subunit, mitochondrial OS=Homo sapiens OX=9606 GN=SDHA PE=1 SV=2	0	10.849	6	4	6	4	664	72.6	7.39	7.01	4	0	2536341.082		NCDN only
Q9Y5V3	Melanoma-associated antigen D1 OS=Homo sapiens OX=9606 GN=MAGED1 PE=1 SV=3	0.002	2.206	1	1	6	1	778	86.1	5.83	1.86	1	0	73180.90625		NCDN only
P07814	Bifunctional glutamate/proline--tRNA ligase OS=Homo sapiens OX=9606 GN=EPRS1 PE=1 SV=5	0	8.949	2	3	6	3	1512	170.5	7.33	9.65	3	0	1105011.244		NCDN only
O14949	Cytochrome b-c1 complex subunit 8 OS=Homo sapiens OX=9606 GN=UQCRQ PE=1 SV=4	0.002	3.213	11	1	6	1	82	9.9	10.08	7.4	1	0	121607.0547		NCDN only
Q7LGA3	Heparan sulfate 2-O-sulfotransferase 1 OS=Homo sapiens OX=9606 GN=HS2ST1 PE=1 SV=1	0	15.148	5	2	6	2	356	41.9	8.69	14.43	2	0	874363.2813		NCDN only
Q14498	RNA-binding protein 39 OS=Homo sapiens OX=9606 GN=RBM39 PE=1 SV=2	0	10.606	4	2	6	2	530	59.3	10.1	11.83	2	0	182897.7969		NCDN only
Q9UKX2	Myosin-2 OS=Homo sapiens OX=9606 GN=MYH2 PE=1 SV=1	0	8.966	1	3	6	1	1941	222.9	5.82	11.12	3	1	738427.4258		NCDN only
Q14344	Guanine nucleotide-binding protein subunit alpha-13 OS=Homo sapiens OX=9606 GN=GNA13 PE=1 SV=2	0	11.647	7	3	6	2	377	44	8	10.42	3	0	309415.9063		NCDN only
O95071	E3 ubiquitin-protein ligase UBR5 OS=Homo sapiens OX=9606 GN=UBR5 PE=1 SV=2	0	4.383	1	2	6	2	2799	309.2	5.85	6.93	2	0	4998447.5		NCDN only
P56545	C-terminal-binding protein 2 OS=Homo sapiens OX=9606 GN=CTBP2 PE=1 SV=1	0	6.633	5	3	6	1	445	48.9	6.95	10.03	3	2	544710.5156		NCDN only

Q8TED1	Probable glutathione peroxidase 8 OS=Homo sapiens OX=9606 GN=GPX8 PE=1 SV=2	0	6.355	9	2	6	2	209	23.9	9.35	6.4	2	0	3373758.922	NCDN only
Q13148	TAR DNA-binding protein 43 OS=Homo sapiens OX=9606 GN=TARDBP PE=1 SV=1	0	10.891	5	2	6	2	414	44.7	6.19	11.01	2	0	88952.08594	NCDN only
O75477	Erlin-1 OS=Homo sapiens OX=9606 GN=ERLIN1 PE=1 SV=2	0	4.339	3	1	5	1	348	39.1	7.87	11.46	1	0	739312.3125	NCDN only
O75054	Immunoglobulin superfamily member 3 OS=Homo sapiens OX=9606 GN=IGSF3 PE=2 SV=3	0.002	2.087	1	1	5	1	1194	135.1	6.07	0	1	0	96940.61328	NCDN only
P50914	60S ribosomal protein L14 OS=Homo sapiens OX=9606 GN=RPL14 PE=1 SV=4	0.001	3.404	3	1	5	1	215	23.4	10.93	1.66	1	0	124011.2227	NCDN only
O14662	Syntaxin-16 OS=Homo sapiens OX=9606 GN=STX16 PE=1 SV=3	0.007	1.77	2	1	5	1	325	37	6.11	3.52	1	0	35205.66797	NCDN only
P33176	Kinesin-1 heavy chain OS=Homo sapiens OX=9606 GN=KIF5B PE=1 SV=1	0	3.996	2	2	5	2	963	109.6	6.51	3.32	2	0	177956.457	NCDN only
Q04637	Eukaryotic translation initiation factor 4 gamma 1 OS=Homo sapiens OX=9606 GN=EIF4G1 PE=1 SV=4	0	5.025	1	2	5	2	1599	175.4	5.33	7.44	2	0	151951.918	NCDN only
Q96PK6	RNA-binding protein 14 OS=Homo sapiens OX=9606 GN=RBM14 PE=1 SV=2	0	4.16	3	2	5	2	669	69.4	9.67	0	2	0	28237.88281	NCDN only
Q8TC07	TBC1 domain family member 15 OS=Homo sapiens OX=9606 GN=TBC1D15 PE=1 SV=2	0	3.802	1	1	5	1	691	79.4	5.67	8.41	1	0	93502.70508	NCDN only
O14757	Serine/threonine-protein kinase Chk1 OS=Homo sapiens OX=9606 GN=CHEK1 PE=1 SV=2	0.002	2.425	2	1	5	1	476	54.4	8.25	5.42	1	0	59138.32422	NCDN only
Q9UI12	V-type proton ATPase subunit H OS=Homo sapiens OX=9606 GN=ATP6V1H PE=1 SV=1	0	7.243	4	2	5	2	483	55.8	6.48	2.7	2	0	127697.3164	NCDN only
Q01814	Plasma membrane calcium-transporting ATPase 2 OS=Homo sapiens OX=9606 GN=ATP2B2 PE=1 SV=2	0	5.748	1	1	5	1	1243	136.8	5.91	12.01	1	0	1058188.043	NCDN only
Q5SWX8	Protein odr-4 homolog OS=Homo sapiens OX=9606 GN=ODR4 PE=1 SV=1	0	13.207	8	4	5	4	454	51.1	5.92	6.49	4	0	198172.9141	NCDN only
Q9HC21	Mitochondrial thiamine pyrophosphate carrier OS=Homo sapiens OX=9606 GN=SLC25A19 PE=1 SV=1	0	4.135	5	2	5	2	320	35.5	9.55	7.62	2	0	984381.7031	NCDN only
P07237	Protein disulfide-isomerase OS=Homo sapiens OX=9606 GN=P4HB PE=1 SV=3	0	13.871	7	3	5	3	508	57.1	4.87	10.62	3	0	305932.1445	NCDN only
Q8TCG1	Protein CIP2A OS=Homo sapiens OX=9606 GN=CIP2A PE=1 SV=2	0	7.206	3	3	5	3	905	102.1	6.23	9.78	3	0	239459.4297	NCDN only
P49006	MARCKS-related protein OS=Homo sapiens OX=9606 GN=MARCKSL1 PE=1 SV=2	0	5.355	7	1	5	1	195	19.5	4.67	9.49	1	0	263746.3438	NCDN only
Q13308	Inactive tyrosine-protein kinase 7 OS=Homo sapiens OX=9606 GN=PTK7 PE=1 SV=2	0	6.841	2	2	5	2	1070	118.3	7.09	8.22	2	0	828123.8203	NCDN only
Q9BQ61	Telomerase RNA component interacting RNase OS=Homo sapiens OX=9606 GN=TRIR PE=1 SV=1	0	6.671	13	2	5	2	176	18.4	9.44	4.37	2	0	361573.4063	NCDN only
Q96EA4	Protein Spindly OS=Homo sapiens OX=9606 GN=SPDL1 PE=1 SV=2	0	6.127	3	2	5	2	605	70.1	5.47	4.37	2	0	99236.04883	NCDN only
Q9Y679	Lipid droplet-regulating VLDL assembly factor AUP1 OS=Homo sapiens OX=9606 GN=AUP1 PE=1 SV=2	0	7.911	7	3	5	3	410	45.8	8.65	9.87	3	0	387380.0313	NCDN only
Q96CS3	FAS-associated factor 2 OS=Homo sapiens OX=9606 GN=FAF2 PE=1 SV=2	0	4.402	4	2	5	2	445	52.6	5.62	7.7	2	0	396786.125	NCDN only
Q9H2V7	Protein spinster homolog 1 OS=Homo sapiens OX=9606 GN=SPNS1 PE=1 SV=1	0.002	2.532	2	1	5	1	528	56.6	6.64	7.77	1	0	187153.6328	NCDN only
P62937	Peptidyl-prolyl cis-trans isomerase A OS=Homo sapiens OX=9606 GN=PPIA PE=1 SV=2	0	6.385	11	2	5	2	165	18	7.81	6.48	2	0	709916.9922	NCDN only
Q6ZRP7	Sulfhydryl oxidase 2 OS=Homo sapiens OX=9606 GN=QSOX2 PE=1 SV=3	0	15.789	7	5	5	5	698	77.5	7.72	10.07	5	0	1438694.563	NCDN only
Q9HC07	Transmembrane protein 165 OS=Homo sapiens OX=9606 GN=TMEM165 PE=1 SV=1	0	48.496	18	4	5	4	324	34.9	7.02	20.76	4	0	14936144.5	NCDN only
P25490	Transcriptional repressor protein YY1 OS=Homo sapiens OX=9606 GN=YY1 PE=1 SV=2	0	4.644	4	2	5	2	414	44.7	6.25	5.9	2	0	419869.8906	NCDN only
O95139	NADH dehydrogenase [ubiquinone] 1 beta subcomplex subunit 6 OS=Homo sapiens OX=9606 GN=NDUFB6 PE=1 SV=3	0	4.307	13	2	5	2	128	15.5	9.63	5.77	2	0	196972.4531	NCDN only
P38919	Eukaryotic initiation factor 4A-III OS=Homo sapiens OX=9606 GN=EIF4A3 PE=1 SV=4	0	4.451	3	1	5	1	411	46.8	6.73	7.45	1	0	84457.56641	NCDN only
Q13363	C-terminal-binding protein 1 OS=Homo sapiens OX=9606 GN=CTBP1 PE=1 SV=2	0	6.552	5	3	5	1	440	47.5	6.77	7.55	3	0	52419.53516	NCDN only
Q14974	Importin subunit beta-1 OS=Homo sapiens OX=9606 GN=KPNB1 PE=1 SV=2	0	5.784	1	1	4	1	876	97.1	4.78	7.51	1	0	1366241.688	NCDN only
P49069	Guided entry of tail-anchored proteins factor CAMLG OS=Homo sapiens OX=9606 GN=CAMLG PE=1 SV=1	0	14.191	12	3	4	3	296	32.9	8.05	4.68	3	0	413845.6445	NCDN only
Q96N66	Lysophospholipid acyltransferase 7 OS=Homo sapiens OX=9606 GN=MBOAT7 PE=1 SV=2	0	6.263	4	2	4	2	472	52.7	8.97	4.17	2	0	1921640.375	NCDN only
Q10471	Polypeptide N-acetylgalactosaminyltransferase 2 OS=Homo sapiens OX=9606 GN=GALNT2 PE=1 SV=1	0	4.315	3	2	4	2	571	64.7	8.35	3.57	2	0	245872.3281	NCDN only
O43761	Synaptogyrin-3 OS=Homo sapiens OX=9606 GN=SYNGR3 PE=1 SV=2	0	5.137	7	1	4	1	229	24.5	8.18	7.2	1	0	89933.22656	NCDN only
Q9HBH5	Retinol dehydrogenase 14 OS=Homo sapiens OX=9606 GN=RDH14 PE=1 SV=1	0	5.859	5	2	4	2	336	36.8	8.79	6.74	2	0	123378.5195	NCDN only
P60866	40S ribosomal protein S20 OS=Homo sapiens OX=9606 GN=RPS20 PE=1 SV=1	0	8.686	23	3	4	3	119	13.4	9.94	7.61	3	0	891942.8125	NCDN only
Q08J23	RNA cytosine C(5)-methyltransferase NSUN2 OS=Homo sapiens OX=9606 GN=NSUN2 PE=1 SV=2	0	6.029	2	2	4	2	767	86.4	6.77	4.22	2	0	34184.95313	NCDN only
Q8IZR5	CKLF-like MARVEL transmembrane domain-containing protein 4 OS=Homo sapiens OX=9606 GN=CMTM4 PE=1 SV=1	0.002	3.045	4	1	4	1	234	25.8	7.75	2.16	1	0	75538.99219	NCDN only
P62241	40S ribosomal protein S8 OS=Homo sapiens OX=9606 GN=RPS8 PE=1 SV=2	0.002	2.618	4	1	4	1	208	24.2	10.32	5.67	1	0	17471.23633	NCDN only
Q14318	Peptidyl-prolyl cis-trans isomerase FKBP8 OS=Homo sapiens OX=9606 GN=FKBP8 PE=1 SV=2	0	5.247	4	2	4	2	412	44.5	4.84	5.48	2	0	317996.9297	NCDN only
Q9Y446	Plakophilin-3 OS=Homo sapiens OX=9606 GN=PKP3 PE=1 SV=1	0	3.568	1	1	4	1	797	87	9.32	8.97	1	0	319432.0234	NCDN only
P20339	Ras-related protein Rab-5A OS=Homo sapiens OX=9606 GN=RAB5A PE=1 SV=2	0.002	2.307	6	1	4	1	215	23.6	8.15	0	1	0	34865.91016	NCDN only
Q8NF37	Lysophosphatidylcholine acyltransferase 1 OS=Homo sapiens OX=9606 GN=LPCAT1 PE=1 SV=2	0	4.819	2	1	4	1	534	59.1	6.02	6.66	1	0	46092.11719	NCDN only
Q86XL3	Ankyrin repeat and LEM domain-containing protein 2 OS=Homo sapiens OX=9606 GN=ANKLE2 PE=1 SV=4	0	5.919	2	2	4	2	938	104.1	7.09	4.06	2	0	422700.1016	NCDN only
O60831	PRA1 family protein 2 OS=Homo sapiens OX=9606 GN=PRAF2 PE=1 SV=1	0	16.326	22	3	4	3	178	19.2	9.19	11.78	3	0	2874580.172	NCDN only
Q9Y3I0	RNA-splicing ligase RtcB homolog OS=Homo sapiens OX=9606 GN=RTCB PE=1 SV=1	0	6.87	3	2	4	2	505	55.2	7.23	4.22	2	0	473536.5703	NCDN only
Q5D862	Filaggrin-2 OS=Homo sapiens OX=9606 GN=FLG2 PE=1 SV=1	0	10.008	1	2	4	2	2391	247.9	8.31	5.54	2	0	1091065.303	NCDN only
P08240	Signal recognition particle receptor subunit alpha OS=Homo sapiens OX=9606 GN=SRPRA PE=1 SV=2	0	5.474	2	2	4	2	638	69.8	8.95	5.86	2	0	404640.4883	NCDN only
Q9NX14	NADH dehydrogenase [ubiquinone] 1 beta subcomplex subunit 11, mitochondrial OS=Homo sapiens OX=9606 GN=NDUFB11 PE=1 SV=1	0.002	3.079	7	1	4	1	153	17.3	5.22	6.02	1	0	424755.5625	NCDN only
Q92616	eIF-2-alpha kinase activator GCN1 OS=Homo sapiens OX=9606 GN=GCN1 PE=1 SV=6	0	6.397	1	3	4	3	2671	292.6	7.47	5.7	3	0	91145.46875	NCDN only
Q9GZM5	Protein YIPF3 OS=Homo sapiens OX=9606 GN=YIPF3 PE=1 SV=1	0	4.747	5	2	4	2	350	38.2	5.76	5.94	2	0	401933.7422	NCDN only
Q96KR6	Protein FAM210B, mitochondrial OS=Homo sapiens OX=9606 GN=FAM210B PE=1 SV=2	0.008	1.581	4	1	4	1	192	20.4	10.43	5.91	1	0	13287698	NCDN only
Q8N4V1	ER membrane protein complex subunit 5 OS=Homo sapiens OX=9606 GN=MMGT1 PE=1 SV=1	0.002	2.533	7	1	4	1	131	14.7	9.16	1.71	1	0	133761.4492	NCDN only
P09543	2',3'-cyclic-nucleotide 3'-phosphodiesterase OS=Homo sapiens OX=9606 GN=CNP PE=1 SV=2	0	5.754	4	2	4	2	421	47.5	9.07	7.26	2	0	74287.46094	NCDN only
P22830	Ferrochelatase, mitochondrial OS=Homo sapiens OX=9606 GN=FECH PE=1 SV=2	0.007	1.697	2	1	4	1	423	47.8	8.73	2.1	1	0	310111.1172	NCDN only
A1L0T0	2-hydroxyacyl-CoA lyase 2 OS=Homo sapiens OX=9606 GN=ILVBL PE=1 SV=2	0	3.78	3	2	4	2	632	67.8	8.15	6.65	2	0	223837.0313	NCDN only
Q92896	Golgi apparatus protein 1 OS=Homo sapiens OX=9606 GN=GLG1 PE=1 SV=2	0	9.299	2	3	4	3	1179	134.5	6.9	8.48	3	0	180584.9063	NCDN only
Q13620	Cullin-4B OS=Homo sapiens OX=9606 GN=CUL4B PE=1 SV=4	0	4.486	2	2	4	2	913	103.9	7.37	0	2	0	88699.24609	NCDN only
Q96S19	Methyltransferase-like 26 OS=Homo sapiens OX=9606 GN=METTL26 PE=1 SV=2	0	6.065	4	1	4	1	204	22.6	7.83	5.7	1	0	242006.5313	NCDN only
Q03252	Lamin-B2 OS=Homo sapiens OX=9606 GN=LMNB2 PE=1 SV=4	0	11.926	5	3	4	1	620	69.9	5.59	7.59	3	0	71171.05469	NCDN only
O94923	D-glucuronyl C5-epimerase OS=Homo sapiens OX=9606 GN=GLCE PE=1 SV=3	0	8.079	5	3	4	3	617	70.1	8.97	6.27	3	0	2205392.289	NCDN only
Q9HD23	Magnesium transporter MRS2 homolog, mitochondrial OS=Homo sapiens OX=9606 GN=MRS2 PE=1 SV=1	0	7.793	6	3	4	3	443	50.3	6.87	5.37	3	0	751514.6563	NCDN only
P51570	Galactokinase OS=Homo sapiens OX=9606 GN=GALK1 PE=1 SV=1	0	4.817	4	2	4	2	392	42.2	6.46	7.75	2	0	160158.4688	NCDN only
P61254	60S ribosomal protein L26 OS=Homo sapiens OX=9606 GN=RPL26 PE=1 SV=1	0.002	2.597	6	1	4	1	145	17.2	10.55	8.74	1	0	141625.3867	NCDN only
Q8TDL5	BPI fold-containing family B member 1 OS=Homo sapiens OX=9606 GN=BPIFB1 PE=1 SV=1	0.002	3.195	2	1	4	1	484	52.4	7.23	0	1	0	10655.24316	NCDN only
P46781	40S ribosomal protein S9 OS=Homo sapiens OX=9606 GN=RPS9 PE=1 SV=3	0	4.075	8	2	4	2	194	22.6	10.65	1.89	2	0	65549.0625	NCDN only
Q5VT66	Mitochondrial amidoxime-reducing component 1 OS=Homo sapiens OX=9606 GN=MTARC1 PE=1 SV=1	0.002	3.004	3	1	4	1	337	37.5	8.88	9.34	1	0	277006.6406	NCDN only
P27105	Stomatin OS=Homo sapiens OX=9606 GN=STOM PE=1 SV=3	0.002	3.194	3	1	4	1	288	31.7	7.88	5.51	1	0	235879.2422	NCDN only
Q6UXV4	MICOS complex subunit MIC27 OS=Homo sapiens OX=9606 GN=APOOL PE=1 SV=1	0	7.032	4	1	4	1	268	29.1	9.52	12.71	1	0	691634	NCDN only
P14868	Aspartate--tRNA ligase, cytoplasmic OS=Homo sapiens OX=9606 GN=DARS1 PE=1 SV=2	0	5.863	3	2	4	2	501	57.1	6.55	4.12	2	0	102226.2012	NCDN only
P48047	ATP synthase subunit O, mitochondrial OS=Homo sapiens OX=9606 GN=ATP5PO PE=1 SV=1	0	7.213	12	3	4	3	213	23.3	9.96	5.56	3	0	208821.4219	NCDN only
O95373	Importin-7 OS=Homo sapiens OX=9606 GN=IPO7 PE=1 SV=1	0	4.124	2	2	4	2	1038	119.4	4.82	3.58	2	0	68253.71094	NCDN only
Q14240	Eukaryotic initiation factor 4A-II OS=Homo sapiens OX=9606 GN=EIF4A2 PE=1 SV=2	0	6.284	5	2	4	2	407	46.4	5.48	7.62	2	0	1466890.813	NCDN only
Q9H936	Mitochondrial glutamate carrier 1 OS=Homo sapiens OX=9606 GN=SLC25A22 PE=1 SV=1	0.001	3.371	2	1	4	1	323	34.4	9.29	7.46	1	0	2220167.383	NCDN only
Q9NRW7	Vacuolar protein sorting-associated protein 45 OS=Homo sapiens OX=9606 GN=VPS45 PE=1 SV=1	0	5.21	3	2	4	2	570	65	8.24	5.51	2	0	464074.1289	NCDN only
Q01081	Splicing factor U2AF 35 kDa subunit OS=Homo sapiens OX=9606 GN=U2AF1 PE=1 SV=3	0	7.828	5	1	4	1	240	27.9	8.81	12.18	1	0	691410.3438	NCDN only
Q15645	Pachytene checkpoint protein 2 homolog OS=Homo sapiens OX=9606 GN=TRIP13 PE=1 SV=2	0	3.871	2	1	4	1	432	48.5	6.09	8.61	1	0	460645.7656	NCDN only
Q7Z406	Myosin-14 OS=Homo sapiens OX=9606 GN=MYH14 PE=1 SV=2	0	10.397	1	3	4	2	1995	227.7	5.6	3.06	3	0	36323.03223	NCDN only
Q8NC56	LEM domain-containing protein 2 OS=Homo sapiens OX=9606 GN=LEMD2 PE=1 SV=1	0	3.715	2	1	4	1	503	56.9	9	6.89	1	0	506824.2813	NCDN only
Q15003	Condensin complex subunit 2 OS=Homo sapiens OX=9606 GN=NCAPH PE=1 SV=3	0	7.014	2	2	4	2	741	82.5	5.06	9.52	2	0	422769.6973	NCDN only
Q7L1Q6	Basic leucine zipper and W2 domain-containing protein 1 OS=Homo sapiens OX=9606 GN=BZW1 PE=1 SV=1	0	5.576	6	3	4	3	419	48	5.92	1.87	3	0	279296.8242	NCDN only
O00303	Eukaryotic translation initiation factor 3 subunit F OS=Homo sapiens OX=9606 GN=EIF3F PE=1 SV=1	0	3.506	3	1	4	1	357	37.5	5.45	5.94	1	0	146126.9844	NCDN only
Q96KC8	DnaJ homolog subfamily C member 1 OS=Homo sapiens OX=9606 GN=DNAJC1 PE=1 SV=1	0	4.413	3	2	4	2	554	63.8	8.63	8.11	2	0	489535.5313	NCDN only
P02786	Transferrin receptor protein 1 OS=Homo sapiens OX=9606 GN=TFRC PE=1 SV=2	0	5.866	2	2	4	2	760	84.8	6.61	4.44	2	0	993572.9531	NCDN only
Q53S58	Transmembrane protein 177 OS=Homo sapiens OX=9606 GN=TMEM177 PE=1 SV=1	0	4.215	5	2	4	2	311	33.7	9.61	6.36	2	0	390900.6582	NCDN only
P53985	Monocarboxylate transporter 1 OS=Homo sapiens OX=9606 GN=SLC16A1 PE=1 SV=3	0	32.54	8	3	4	3	500	53.9	8.66	11.44	3	0	8631145.75	NCDN only
Q9UJZ1	Stomatin-like protein 2, mitochondrial OS=Homo sapiens OX=9606 GN=STOML2 PE=1 SV=1	0	13.244	7	2	4	2	356	38.5	7.39	10.42	2	0	1076012.227	NCDN only
Q9P0T7	Proton-transporting V-type ATPase complex assembly regulator TMEM9 OS=Homo sapiens OX=9606 GN=TMEM9 PE=1 SV=1	0	3.952	11	2	3	2	183	20.6	6.65	3.85	2	0	97652.57031	NCDN only
O75381	Peroxisomal membrane protein PEX14 OS=Homo sapiens OX=9606 GN=PEX14 PE=1 SV=1	0	6.903	7	1	3	1	377	41.2	4.94	9.71	1	0	117941.5078	NCDN only
Q9UBS4	DnaJ homolog subfamily B member 11 OS=Homo sapiens OX=9606 GN=DNAJB11 PE=1 SV=1	0.002	2.372	2	1	3	1	358	40.5	6.18	6.21	1	0	90028.10156	NCDN only
Q8NBU5	Outer mitochondrial transmembrane helix translocase OS=Homo sapiens OX=9606 GN=ATAD1 PE=1 SV=1	0	9.63	3	1	3	1	361	40.7	6.9	7.59	1	0	150016.9375	NCDN only
Q86VP6	Cullin-associated NEDD8-dissociated protein 1 OS=Homo sapiens OX=9606 GN=CAND1 PE=1 SV=2	0	9.642	2	3	3	3	1230	136.3	5.78	3.95	3	0	57039.66406	NCDN only
Q8NCW6	Polypeptide N-acetylgalactosaminyltransferase 11 OS=Homo sapiens OX=9606 GN=GALNT11 PE=1 SV=2	0	4.871	3	2	3	2	608	68.9	8.19	4	2	0	23543.86133	NCDN only
P14625	Endoplasmin OS=Homo sapiens OX=9606 GN=HSP90B1 PE=1 SV=1	0	6.95	2	2	3	1	803	92.4	4.84	5.32	2	0	86192.12109	NCDN only
P24311	Cytochrome c oxidase subunit 7B, mitochondrial OS=Homo sapiens OX=9606 GN=COX7B PE=1 SV=2	0.005	1.928	9	1	3	1	80	9.2	10.27	1.69	1	0	72007.63281	NCDN only
P28331	NADH-ubiquinone oxidoreductase 75 kDa subunit, mitochondrial OS=Homo sapiens OX=9606 GN=NDUFS1 PE=1 SV=3	0	4.697	1	1	3	1	727	79.4	6.23	6.85	1	0	478352.1875	NCDN only
O15260	Surfeit locus protein 4 OS=Homo sapiens OX=9606 GN=SURF4 PE=1 SV=3	0	5.457	4	1	3	1	269	30.4	7.78	6.59	1	0	6649401.875	NCDN only
Q9Y5Y0	Feline leukemia virus subgroup C receptor-related protein 1 OS=Homo sapiens OX=9606 GN=FLVCR1 PE=1 SV=1	0	13.473	8	3	3	3	555	59.8	5.97	5.82	3	0	18452.33789	NCDN only
P14174	Macrophage migration inhibitory factor OS=Homo sapiens OX=9606 GN=MIF PE=1 SV=4	0.016	1.461	6	1	3	1	115	12.5	7.88	4.18	1	0	1220257.75	NCDN only
P43487	Ran-specific GTPase-activating protein OS=Homo sapiens OX=9606 GN=RANBP1 PE=1 SV=1	0	4.295	4	1	3	1	201	23.3	5.29	4.3	1	0	194696.5156	NCDN only
Q9BW27	Nuclear pore complex protein Nup85 OS=Homo sapiens OX=9606 GN=NUP85 PE=1 SV=1	0	4.822	2	2	3	2	656	75	5.55	3.95	2	0	133716.3047	NCDN only
Q96LW7	Caspase recruitment domain-containing protein 19 OS=Homo sapiens OX=9606 GN=CARD19 PE=1 SV=1	0.002	2.681	4	1	3	1	228	25.6	9.2	4.25	1	0	119196.7109	NCDN only
Q16531	DNA damage-binding protein 1 OS=Homo sapiens OX=9606 GN=DDB1 PE=1 SV=1	0	5.308	2	3	3	3	1140	126.9	5.26	3.73	3	0	1615046.545	NCDN only
Q969T9	WW domain-binding protein 2 OS=Homo sapiens OX=9606 GN=WBP2 PE=1 SV=1	0	4.423	4	1	3	1	261	28.1	5.91	6.36	1	0	2395849.25	NCDN only
Q14165	Malectin OS=Homo sapiens OX=9606 GN=MLEC PE=1 SV=1	0.002	2.128	2	1	3	1	292	32.2	5.41	3.65	1	0	160226.5977	NCDN only
Q9NXW2	DnaJ homolog subfamily B member 12 OS=Homo sapiens OX=9606 GN=DNAJB12 PE=1 SV=5	0.004	2.014	2	1	3	1	375	41.8	8.69	1.65	1	0	25382.98242	NCDN only
Q9H0C8	Integrin-linked kinase-associated serine/threonine phosphatase 2C OS=Homo sapiens OX=9606 GN=ILKAP PE=1 SV=1	0.004	2.02	2	1	3	1	392	42.9	7.09	3.83	1	0	77814.04688	NCDN only
Q9UBV8	Peflin OS=Homo sapiens OX=9606 GN=PEF1 PE=1 SV=1	0.007	1.712	3	1	3	1	284	30.4	6.54	1.77	1	0	47724.04297	NCDN only
Q15046	Lysine--tRNA ligase OS=Homo sapiens OX=9606 GN=KARS1 PE=1 SV=3	0	4.929	3	2	3	2	597	68	6.35	4.52	2	0	31391.8457	NCDN only
Q9NX76	CKLF-like MARVEL transmembrane domain-containing protein 6 OS=Homo sapiens OX=9606 GN=CMTM6 PE=1 SV=1	0.001	3.407	16	2	3	2	183	20.4	5.29	4.69	2	0	395912.375	NCDN only
P51665	26S proteasome non-ATPase regulatory subunit 7 OS=Homo sapiens OX=9606 GN=PSMD7 PE=1 SV=2	0.002	2.433	2	1	3	1	324	37	6.77	6.99	1	0	169733.9141	NCDN only
O00311	Cell division cycle 7-related protein kinase OS=Homo sapiens OX=9606 GN=CDC7 PE=1 SV=1	0	5.465	3	2	3	2	574	63.8	8.73	3.29	2	0	87815.89063	NCDN only
P63218	Guanine nucleotide-binding protein G(I)/G(S)/G(O) subunit gamma-5 OS=Homo sapiens OX=9606 GN=GNG5 PE=1 SV=3	0.001	3.332	13	1	3	1	68	7.3	9.85	8.42	1	0	486611.1719	NCDN only
Q9NX40	OCIA domain-containing protein 1 OS=Homo sapiens OX=9606 GN=OCIAD1 PE=1 SV=1	0	4.897	4	1	3	1	245	27.6	7.49	8.74	1	0	1387030.125	NCDN only
Q8IYS2	Uncharacterized protein KIAA2013 OS=Homo sapiens OX=9606 GN=KIAA2013 PE=1 SV=1	0	7.197	4	2	3	2	634	69.1	8.19	6.42	2	0	2228510.25	NCDN only
Q9BXP8	Pappalysin-2 OS=Homo sapiens OX=9606 GN=PAPPA2 PE=1 SV=4	0.002	3.087	1	1	3	1	1791	198.4	5.47	8.68	1	0	2314218.75	NCDN only
Q96N67	Dedicator of cytokinesis protein 7 OS=Homo sapiens OX=9606 GN=DOCK7 PE=1 SV=4	0	5.595	1	3	3	3	2140	242.4	6.8	0	3	0	86703.40625	NCDN only
Q6P1J9	Parafibromin OS=Homo sapiens OX=9606 GN=CDC73 PE=1 SV=1	0	5.639	3	2	3	2	531	60.5	9.61	6.08	2	0	150759.2656	NCDN only
Q8WVM8	Sec1 family domain-containing protein 1 OS=Homo sapiens OX=9606 GN=SCFD1 PE=1 SV=4	0	6.972	4	3	3	3	642	72.3	6.27	2.09	3	0	22926.80859	NCDN only
Q14210	Lymphocyte antigen 6D OS=Homo sapiens OX=9606 GN=LY6D PE=1 SV=1	0.002	2.365	7	1	3	1	128	13.3	8.21	3.48	1	0	29055.66406	NCDN only
O95299	NADH dehydrogenase [ubiquinone] 1 alpha subcomplex subunit 10, mitochondrial OS=Homo sapiens OX=9606 GN=NDUFA10 PE=1 SV=1	0.004	2.022	2	1	3	1	355	40.7	8.48	0	1	0	13850.80078	NCDN only
O75663	TIP41-like protein OS=Homo sapiens OX=9606 GN=TIPRL PE=1 SV=2	0	3.775	4	1	3	1	272	31.4	5.91	5.36	1	0	229875.2422	NCDN only
O94991	SLIT and NTRK-like protein 5 OS=Homo sapiens OX=9606 GN=SLITRK5 PE=1 SV=2	0.005	1.829	1	1	3	1	958	107.4	6.95	5.54	1	0	91818.85938	NCDN only
Q9HAV4	Exportin-5 OS=Homo sapiens OX=9606 GN=XPO5 PE=1 SV=1	0	4.521	1	2	3	2	1204	136.2	5.8	5.16	2	0	49346.82813	NCDN only

Q8IWC1	MAP7 domain-containing protein 3 OS=Homo sapiens OX=9606 GN=MAP7D3 PE=1 SV=2	0	7.028	1	1	3	1	876	98.4	9.32	10.42	1	0	683759.3125	NCDN only
P60953	Cell division control protein 42 homolog OS=Homo sapiens OX=9606 GN=CDC42 PE=1 SV=2	0	8.198	12	2	3	2	191	21.2	6.55	6.24	2	0	268475.4121	NCDN only
Q9NVH0	Exonuclease 3'-5' domain-containing protein 2 OS=Homo sapiens OX=9606 GN=EXD2 PE=1 SV=2	0	5.414	4	2	3	2	621	70.3	8.32	4.12	2	0	103458.5352	NCDN only
O95674	Phosphatidate cytidylyltransferase 2 OS=Homo sapiens OX=9606 GN=CDS2 PE=1 SV=1	0	9.669	6	2	3	2	445	51.4	7.09	7.94	2	0	60044.75781	NCDN only
Q9UK73	Protein fem-1 homolog B OS=Homo sapiens OX=9606 GN=FEM1B PE=1 SV=1	0.002	2.351	2	1	3	1	627	70.2	6.61	0	1	0	35541.75391	NCDN only
O15120	1-acyl-sn-glycerol-3-phosphate acyltransferase beta OS=Homo sapiens OX=9606 GN=AGPAT2 PE=1 SV=1	0	5.047	9	2	3	2	278	30.9	9.01	1.96	2	0	27913.33984	NCDN only
P13073	Cytochrome c oxidase subunit 4 isoform 1, mitochondrial OS=Homo sapiens OX=9606 GN=COX4I1 PE=1 SV=1	0.002	3.108	5	1	3	1	169	19.6	9.51	3.95	1	0	130946.7188	NCDN only
Q96F86	Enhancer of mRNA-decapping protein 3 OS=Homo sapiens OX=9606 GN=EDC3 PE=1 SV=1	0	5.214	4	2	3	2	508	56	7.11	5.87	2	0	81171.41992	NCDN only
P06576	ATP synthase subunit beta, mitochondrial OS=Homo sapiens OX=9606 GN=ATP5F1B PE=1 SV=3	0	18.414	9	3	3	3	529	56.5	5.4	6.33	3	0	2791695	NCDN only
P14866	Heterogeneous nuclear ribonucleoprotein L OS=Homo sapiens OX=9606 GN=HNRNPL PE=1 SV=2	0	4.629	3	2	3	2	589	64.1	8.22	2.28	2	0	1171423.25	NCDN only
Q16611	Bcl-2 homologous antagonist/killer OS=Homo sapiens OX=9606 GN=BAK1 PE=1 SV=1	0	3.924	5	1	3	1	211	23.4	6.01	4.3	1	0	27476.34961	NCDN only
Q8IWI9	MAX gene-associated protein OS=Homo sapiens OX=9606 GN=MGA PE=1 SV=4	0	4.261	1	2	3	2	3065	336	6.9	1.68	2	0	53071.59766	NCDN only
Q15070	Mitochondrial inner membrane protein OXA1L OS=Homo sapiens OX=9606 GN=OXA1L PE=1 SV=3	0	7.49	2	1	3	1	435	48.5	9.45	5.44	1	0	1057913.828	NCDN only
Q8IXK2	Polypeptide N-acetylgalactosaminyltransferase 12 OS=Homo sapiens OX=9606 GN=GALNT12 PE=1 SV=3	0.007	1.689	1	1	3	1	581	66.9	6.8	3.78	1	0	76336.52344	NCDN only
O43291	Kunitz-type protease inhibitor 2 OS=Homo sapiens OX=9606 GN=SPINT2 PE=1 SV=2	0	3.678	5	1	3	1	252	28.2	8.29	5.92	1	0	300258.6328	NCDN only
P55786	Puromycin-sensitive aminopeptidase OS=Homo sapiens OX=9606 GN=NPEPPS PE=1 SV=2	0.002	3.19	2	2	3	2	919	103.2	5.72	1.65	2	0	107381.5156	NCDN only
P55265	Double-stranded RNA-specific adenosine deaminase OS=Homo sapiens OX=9606 GN=ADAR PE=1 SV=4	0	4.238	1	2	3	2	1226	136	8.65	3.47	2	0	31083.65625	NCDN only
Q9P2R3	Rabankyrin-5 OS=Homo sapiens OX=9606 GN=ANKFY1 PE=1 SV=2	0	6.451	2	2	3	2	1169	128.3	6.1	4.57	2	0	628618.1875	NCDN only
Q6P6B1	Glutamate-rich protein 5 OS=Homo sapiens OX=9606 GN=ERICH5 PE=1 SV=1	0.005	1.898	3	1	3	1	374	39.9	4.49	0	1	0	65296.27539	NCDN only
Q00610	Clathrin heavy chain 1 OS=Homo sapiens OX=9606 GN=CLTC PE=1 SV=5	0	6.695	1	3	3	3	1675	191.5	5.69	1.69	3	0	69999.36816	NCDN only
O75063	Glycosaminoglycan xylosylkinase OS=Homo sapiens OX=9606 GN=FAM20B PE=1 SV=1	0	4.345	4	2	3	2	409	46.4	6.87	1.85	2	0	107043.4844	NCDN only
P15735	Phosphorylase b kinase gamma catalytic chain, liver/testis isoform OS=Homo sapiens OX=9606 GN=PHKG2 PE=1 SV=1	0.002	2.136	2	1	3	1	406	46.4	6.38	0	1	0	704455.75	NCDN only
Q9BTE3	Mini-chromosome maintenance complex-binding protein OS=Homo sapiens OX=9606 GN=MCMBP PE=1 SV=2	0	8.722	3	2	3	2	642	72.9	5.87	7.78	2	0	544311.3438	NCDN only
P83881	60S ribosomal protein L36a OS=Homo sapiens OX=9606 GN=RPL36A PE=1 SV=2	0.002	2.259	8	1	3	1	106	12.4	10.58	1.99	1	0	62009.85156	NCDN only
Q8N2U0	Transmembrane protein 256 OS=Homo sapiens OX=9606 GN=TMEM256 PE=3 SV=1	0.002	2.178	8	1	3	1	113	11.7	8.94	2.07	1	0	553569.0313	NCDN only
O14681	Etoposide-induced protein 2.4 homolog OS=Homo sapiens OX=9606 GN=EI24 PE=1 SV=4	0.002	2.276	2	1	3	1	340	38.9	9.72	7.39	1	0	276798.9688	NCDN only
Q13435	Splicing factor 3B subunit 2 OS=Homo sapiens OX=9606 GN=SF3B2 PE=1 SV=2	0.002	3.188	1	1	3	1	895	100.2	5.67	1.87	1	0	287056.8125	NCDN only
P09651	Heterogeneous nuclear ribonucleoprotein A1 OS=Homo sapiens OX=9606 GN=HNRNPA1 PE=1 SV=5	0	12.849	5	1	3	1	372	38.7	9.13	11.67	1	0	68089.83984	NCDN only
Q9BU23	Lipase maturation factor 2 OS=Homo sapiens OX=9606 GN=LMF2 PE=1 SV=2	0.002	2.292	1	1	3	1	707	79.6	10.1	4.02	1	0	241535.75	NCDN only
P23763	Vesicle-associated membrane protein 1 OS=Homo sapiens OX=9606 GN=VAMP1 PE=1 SV=1	0.007	1.727	6	1	2	1	118	12.9	6.65	1.9	1	0	51569.66016	NCDN only
O75386	Tubby-related protein 3 OS=Homo sapiens OX=9606 GN=TULP3 PE=1 SV=2	0.022	1.415	2	1	2	1	442	49.6	7.74	0	1	0	33500.57813	NCDN only
O95772	STARD3 N-terminal-like protein OS=Homo sapiens OX=9606 GN=STARD3NL PE=1 SV=1	0.011	1.526	3	1	2	1	234	26.6	4.81	1.72	1	0	134867.9531	NCDN only
P10644	cAMP-dependent protein kinase type I-alpha regulatory subunit OS=Homo sapiens OX=9606 GN=PRKAR1A PE=1 SV=1	0	3.698	4	2	2	2	381	43	5.35	2.16	2	0	32518.5957	NCDN only
Q9NVI1	Fanconi anemia group I protein OS=Homo sapiens OX=9606 GN=FANCI PE=1 SV=4	0	5.279	1	2	2	2	1328	149.2	6.74	3.65	2	0	187979.0469	NCDN only
Q9Y3D7	Mitochondrial import inner membrane translocase subunit TIM16 OS=Homo sapiens OX=9606 GN=PAM16 PE=1 SV=2	0.002	2.921	6	1	2	1	125	13.8	9.7	1.74	1	0	106264.1484	NCDN only
P15531	Nucleoside diphosphate kinase A OS=Homo sapiens OX=9606 GN=NME1 PE=1 SV=1	0	23.851	11	1	2	1	152	17.1	6.19	10.4	1	0	6565536	NCDN only
Q8NE71	ATP-binding cassette sub-family F member 1 OS=Homo sapiens OX=9606 GN=ABCF1 PE=1 SV=2	0	3.913	1	1	2	1	845	95.9	6.8	2.16	1	0	231047.2891	NCDN only
Q9UDX5	Mitochondrial fission process protein 1 OS=Homo sapiens OX=9606 GN=MTFP1 PE=1 SV=1	0	3.722	7	1	2	1	166	18	9.31	4.83	1	0	839582.5	NCDN only
Q9NR30	Nucleolar RNA helicase 2 OS=Homo sapiens OX=9606 GN=DDX21 PE=1 SV=5	0.007	1.751	1	1	2	1	783	87.3	9.28	4.14	1	0	588580	NCDN only
P13667	Protein disulfide-isomerase A4 OS=Homo sapiens OX=9606 GN=PDIA4 PE=1 SV=2	0.006	1.802	1	1	2	1	645	72.9	5.07	1.9	1	0	32760.47656	NCDN only
Q9BUZ4	TNF receptor-associated factor 4 OS=Homo sapiens OX=9606 GN=TRAF4 PE=1 SV=1	0.008	1.622	1	1	2	1	470	53.5	8.15	3.99	1	0	297368.875	NCDN only
P63220	40S ribosomal protein S21 OS=Homo sapiens OX=9606 GN=RPS21 PE=1 SV=1	0	7.023	23	2	2	2	83	9.1	8.5	5.14	2	0	404997.3594	NCDN only
Q9Y256	CAAX prenyl protease 2 OS=Homo sapiens OX=9606 GN=RCE1 PE=1 SV=1	0.005	1.952	3	1	2	1	329	35.8	7.97	4.3	1	0	300917.1875	NCDN only
O60313	Dynamin-like 120 kDa protein, mitochondrial OS=Homo sapiens OX=9606 GN=OPA1 PE=1 SV=3	0	5.482	1	1	2	1	960	111.6	7.87	4.32	1	0	101274.8203	NCDN only
Q96CS2	HAUS augmin-like complex subunit 1 OS=Homo sapiens OX=9606 GN=HAUS1 PE=1 SV=1	0.002	2.669	3	1	2	1	278	31.8	5.53	1.76	1	0	117188.9805	NCDN only
Q8NFF5	FAD synthase OS=Homo sapiens OX=9606 GN=FLAD1 PE=1 SV=1	0	5.08	3	2	2	2	587	65.2	6.93	2.04	2	0	160648.6094	NCDN only
Q9UL15	BAG family molecular chaperone regulator 5 OS=Homo sapiens OX=9606 GN=BAG5 PE=1 SV=1	0.003	2.049	2	1	2	1	447	51.2	6.05	1.8	1	0	69255.08594	NCDN only
Q8NE62	Choline dehydrogenase, mitochondrial OS=Homo sapiens OX=9606 GN=CHDH PE=1 SV=2	0.016	1.475	1	1	2	1	594	65.3	8.28	0	1	0	29696.41211	NCDN only
Q9BZG1	Ras-related protein Rab-34 OS=Homo sapiens OX=9606 GN=RAB34 PE=1 SV=1	0.002	2.306	3	1	2	1	259	29	7.88	3.6	1	0	206845.4531	NCDN only
Q9H5V9	STING ER exit protein OS=Homo sapiens OX=9606 GN=STEEP1 PE=1 SV=1	0.005	1.963	3	1	2	1	222	25.6	8.73	3.81	1	0	51678.98438	NCDN only
Q02878	60S ribosomal protein L6 OS=Homo sapiens OX=9606 GN=RPL6 PE=1 SV=3	0.019	1.439	2	1	2	1	288	32.7	10.58	0	1	0	170354.375	NCDN only
Q96JX3	Protein SERAC1 OS=Homo sapiens OX=9606 GN=SERAC1 PE=2 SV=1	0.001	3.35	1	1	2	1	654	74.1	7.68	3.54	1	0	39850.69141	NCDN only
Q96GD4	Aurora kinase B OS=Homo sapiens OX=9606 GN=AURKB PE=1 SV=3	0.001	3.369	3	1	2	1	344	39.3	9.29	4.26	1	0	94826.94531	NCDN only
Q14739	Delta(14)-sterol reductase LBR OS=Homo sapiens OX=9606 GN=LBR PE=1 SV=2	0	4.29	3	2	2	2	615	70.7	9.36	2.31	2	0	10683145.5	NCDN only
Q6P4A7	Sideroflexin-4 OS=Homo sapiens OX=9606 GN=SFXN4 PE=1 SV=1	0	7	4	1	2	1	337	38	9.19	5.69	1	0	146193.625	NCDN only
O43583	Density-regulated protein OS=Homo sapiens OX=9606 GN=DENR PE=1 SV=2	0.002	2.601	4	1	2	1	198	22.1	5.3	3.87	1	0	67046.71875	NCDN only
P38159	RNA-binding motif protein, X chromosome OS=Homo sapiens OX=9606 GN=RBMX PE=1 SV=3	0	4.68	4	2	2	2	391	42.3	10.05	1.89	2	0	90021.69727	NCDN only
Q99460	26S proteasome non-ATPase regulatory subunit 1 OS=Homo sapiens OX=9606 GN=PSMD1 PE=1 SV=2	0.002	2.082	1	1	2	1	953	105.8	5.39	3.7	1	0	75730.66406	NCDN only
P62917	60S ribosomal protein L8 OS=Homo sapiens OX=9606 GN=RPL8 PE=1 SV=2	0.011	1.565	3	1	2	1	257	28	11.03	4.35	1	0	610876.75	NCDN only
P52948	Nuclear pore complex protein Nup98-Nup96 OS=Homo sapiens OX=9606 GN=NUP98 PE=1 SV=4	0.017	1.448	0	1	2	1	1817	197.5	6.4	0	1	0	31051.44922	NCDN only
Q9Y5S9	RNA-binding protein 8A OS=Homo sapiens OX=9606 GN=RBM8A PE=1 SV=1	0.002	2.354	5	1	2	1	174	19.9	5.72	1.74	1	0	114792.1484	NCDN only
O00422	Histone deacetylase complex subunit SAP18 OS=Homo sapiens OX=9606 GN=SAP18 PE=1 SV=1	0	3.737	10	2	2	2	153	17.6	9.35	0	2	0	35150.47656	NCDN only
O43934	UNC93-like protein MFSD11 OS=Homo sapiens OX=9606 GN=MFSD11 PE=1 SV=2	0.002	2.857	2	1	2	1	449	49.2	5.68	4.6	1	0	127787.5703	NCDN only
P62269	40S ribosomal protein S18 OS=Homo sapiens OX=9606 GN=RPS18 PE=1 SV=3	0.007	1.711	5	1	2	1	152	17.7	10.99	1.8	1	0	66173.71875	NCDN only
Q8TF66	Leucine-rich repeat-containing protein 15 OS=Homo sapiens OX=9606 GN=LRRC15 PE=2 SV=2	0	3.714	2	1	2	1	581	64.3	6.71	4.96	1	0	104730.3203	NCDN only
P07196	Neurofilament light polypeptide OS=Homo sapiens OX=9606 GN=NEFL PE=1 SV=3	0	5.138	3	2	2	2	543	61.5	4.65	2.16	2	0	59203.16406	NCDN only
Q15369	Elongin-C OS=Homo sapiens OX=9606 GN=ELOC PE=1 SV=1	0	13.932	27	2	2	2	112	12.5	4.78	4	2	0	18040.4043	NCDN only
Q15149	Plectin OS=Homo sapiens OX=9606 GN=PLEC PE=1 SV=3	0	5.838	1	2	2	2	4684	531.5	5.96	3.45	2	0	75642.96094	NCDN only
P04844	Dolichyl-diphosphooligosaccharide--protein glycosyltransferase subunit 2 OS=Homo sapiens OX=9606 GN=RPN2 PE=1 SV=3	0.002	2.857	1	1	2	1	631	69.2	5.69	2.27	1	0	832476.3203	NCDN only
Q66LE6	Serine/threonine-protein phosphatase 2A 55 kDa regulatory subunit B delta isoform OS=Homo sapiens OX=9606 GN=PPP2R2D PE=1 SV=1	0.005	1.898	2	1	2	1	453	52	6.39	3.65	1	0	44344.76172	NCDN only
Q8TCU4	Alstrom syndrome protein 1 OS=Homo sapiens OX=9606 GN=ALMS1 PE=1 SV=4	0	4.142	0	2	2	2	4168	460.8	6.28	0	2	0	20958.08398	NCDN only
Q8NBQ5	Estradiol 17-beta-dehydrogenase 11 OS=Homo sapiens OX=9606 GN=HSD17B11 PE=1 SV=3	0.002	2.842	3	1	2	1	300	32.9	9.07	3.87	1	0	22143.91406	NCDN only
A0A0U1RRL7	Protein MMP24OS OS=Homo sapiens OX=9606 GN=MMP24OS PE=1 SV=1	0	10.67	37	1	2	1	71	7.7	4.72	8.09	1	0	136855.8906	NCDN only
Q9HBH1	Peptide deformylase, mitochondrial OS=Homo sapiens OX=9606 GN=PDF PE=1 SV=1	0.002	2.359	3	1	2	1	243	27	9.16	4.25	1	0	311094.6563	NCDN only
P02675	Fibrinogen beta chain OS=Homo sapiens OX=9606 GN=FGB PE=1 SV=2	0.002	2.525	2	1	2	1	491	55.9	8.27	3.8	1	0	42075.23047	NCDN only
Q96EE3	Nucleoporin SEH1 OS=Homo sapiens OX=9606 GN=SEH1L PE=1 SV=3	0.002	2.095	2	1	2	1	360	39.6	8.09	0	1	0	44699.08984	NCDN only
Q8IW41	MAP kinase-activated protein kinase 5 OS=Homo sapiens OX=9606 GN=MAPKAPK5 PE=1 SV=2	0.005	1.959	2	1	2	1	473	54.2	7.78	2.15	1	0	25913.53125	NCDN only
P15170	Eukaryotic peptide chain release factor GTP-binding subunit ERF3A OS=Homo sapiens OX=9606 GN=GSPT1 PE=1 SV=1	0.002	2.459	1	1	2	1	499	55.7	5.62	0	1	0	19363.84961	NCDN only
Q9BQT8	Mitochondrial 2-oxodicarboxylate carrier OS=Homo sapiens OX=9606 GN=SLC25A21 PE=1 SV=1	0.008	1.609	2	1	2	1	299	33.3	9.51	4.24	1	0	292857.0391	NCDN only
Q9Y467	Sal-like protein 2 OS=Homo sapiens OX=9606 GN=SALL2 PE=1 SV=4	0.002	2.057	1	1	2	1	1007	105.2	6.3	1.85	1	0	22377.70117	NCDN only
P55789	FAD-linked sulfhydryl oxidase ALR OS=Homo sapiens OX=9606 GN=GFER PE=1 SV=2	0.005	1.818	12	1	2	1	205	23.4	7.62	2.4	1	0	33457.63281	NCDN only
Q8IWS0	PHD finger protein 6 OS=Homo sapiens OX=9606 GN=PHF6 PE=1 SV=1	0.007	1.696	2	1	2	1	365	41.3	8.68	0	1	0	99544.90625	NCDN only
P07195	L-lactate dehydrogenase B chain OS=Homo sapiens OX=9606 GN=LDHB PE=1 SV=2	0	5.53	5	2	2	1	334	36.6	6.05	1.78	2	0	25843.23242	NCDN only
P61221	ATP-binding cassette sub-family E member 1 OS=Homo sapiens OX=9606 GN=ABCE1 PE=1 SV=1	0	6.21	4	2	2	2	599	67.3	8.34	2.63	2	0	74892.33594	NCDN only
O00425	Insulin-like growth factor 2 mRNA-binding protein 3 OS=Homo sapiens OX=9606 GN=IGF2BP3 PE=1 SV=2	0.002	2.341	2	1	2	1	579	63.7	8.87	0	1	0	56045.05664	NCDN only
Q9Y265	RuvB-like 1 OS=Homo sapiens OX=9606 GN=RUVBL1 PE=1 SV=1	0	8.243	5	2	2	2	456	50.2	6.42	5.11	2	0	124550.5234	NCDN only
O94826	Mitochondrial import receptor subunit TOM70 OS=Homo sapiens OX=9606 GN=TOMM70 PE=1 SV=1	0.005	1.872	1	1	2	1	608	67.4	7.12	1.86	1	0	121133.8008	NCDN only
Q96AA3	Protein RFT1 homolog OS=Homo sapiens OX=9606 GN=RFT1 PE=1 SV=1	0.005	1.833	1	1	2	1	541	60.3	8.85	1.91	1	0	21939.17578	NCDN only
Q96L58	Beta-1,3-galactosyltransferase 6 OS=Homo sapiens OX=9606 GN=B3GALT6 PE=1 SV=2	0.002	2.965	2	1	2	1	329	37.1	9.66	4.08	1	0	138043.9063	NCDN only
Q8N4L2	Type 2 phosphatidylinositol 4,5-bisphosphate 4-phosphatase OS=Homo sapiens OX=9606 GN=PIP4P2 PE=1 SV=1	0	4.998	8	2	2	2	257	28.1	8.68	2.15	2	0	59209.73828	NCDN only
Q8TEM1	Nuclear pore membrane glycoprotein 210 OS=Homo sapiens OX=9606 GN=NUP210 PE=1 SV=3	0.001	3.341	1	2	2	2	1887	205	6.81	3.53	2	0	55168.58594	NCDN only
P12277	Creatine kinase B-type OS=Homo sapiens OX=9606 GN=CKB PE=1 SV=1	0	9.075	9	2	2	2	381	42.6	5.59	2.13	2	0	76881.49219	NCDN only
Q5VYK3	Proteasome adapter and scaffold protein ECM29 OS=Homo sapiens OX=9606 GN=ECPAS PE=1 SV=2	0	4.936	1	2	2	2	1845	204.2	7.12	3.85	2	0	56621.89063	NCDN only
Q9ULX6	A-kinase anchor protein 8-like OS=Homo sapiens OX=9606 GN=AKAP8L PE=1 SV=4	0.002	2.578	1	1	2	1	646	71.6	5.05	3.72	1	0	62369.05469	NCDN only
P27544	Ceramide synthase 1 OS=Homo sapiens OX=9606 GN=CERS1 PE=1 SV=1	0	33.208	7	1	2	1	350	39.5	9.04	13.71	1	0	883476.375	NCDN only
P22314	Ubiquitin-like modifier-activating enzyme 1 OS=Homo sapiens OX=9606 GN=UBA1 PE=1 SV=3	0.007	1.752	1	1	2	1	1058	117.8	5.76	1.79	1	0	474668.4219	NCDN only
Q14108	Lysosome membrane protein 2 OS=Homo sapiens OX=9606 GN=SCARB2 PE=1 SV=2	0.002	2.419	2	1	2	1	478	54.3	5.14	3.82	1	0	170770.2656	NCDN only
Q96H79	Zinc finger CCCH-type antiviral protein 1-like OS=Homo sapiens OX=9606 GN=ZC3HAV1L PE=1 SV=2	0.006	1.784	3	1	2	1	300	32.9	8.13	3.63	1	0	133712.6875	NCDN only
Q6KCM7	Calcium-binding mitochondrial carrier protein SCaMC-2 OS=Homo sapiens OX=9606 GN=SLC25A25 PE=1 SV=1	0.017	1.446	2	1	2	1	469	52.6	8.35	4.2	1	0	265012.3281	NCDN only
Q5JTH9	RRP12-like protein OS=Homo sapiens OX=9606 GN=RRP12 PE=1 SV=2	0	3.876	1	1	2	1	1297	143.6	8.75	2.17	1	0	61793.74219	NCDN only
Q15041	ADP-ribosylation factor-like protein 6-interacting protein 1 OS=Homo sapiens OX=9606 GN=ARL6IP1 PE=1 SV=2	0.002	3.007	4	1	2	1	203	23.3	9.32	2.02	1	0	499479.5742	NCDN only
Q86WJ1	Chromodomain-helicase-DNA-binding protein 1-like OS=Homo sapiens OX=9606 GN=CHD1L PE=1 SV=3	0.005	1.954	1	1	2	1	897	100.9	6.9	3.68	1	0	39956.53906	NCDN only
P02774	Vitamin D-binding protein OS=Homo sapiens OX=9606 GN=GC PE=1 SV=2	0.002	2.075	2	1	2	1	474	52.9	5.45	1.78	1	0	86911.75977	NCDN only
P02787	Serotransferrin OS=Homo sapiens OX=9606 GN=TF PE=1 SV=3	0.012	1.506	2	1	2	1	698	77	7.12	0	1	0	573591.6875	NCDN only
Q16181	Septin-7 OS=Homo sapiens OX=9606 GN=SEPTIN7 PE=1 SV=2	0	4.284	2	1	2	1	437	50.6	8.63	4.01	1	0	44350.34375	NCDN only
Q96A72	Protein mago nashi homolog 2 OS=Homo sapiens OX=9606 GN=MAGOHB PE=1 SV=1	0.002	2.514	5	1	2	1	148	17.3	6.39	0	1	0	32874.60547	NCDN only
P83731	60S ribosomal protein L24 OS=Homo sapiens OX=9606 GN=RPL24 PE=1 SV=1	0.008	1.581	5	1	2	1	157	17.8	11.25	1.6	1	0	36747.24609	NCDN only
O95197	Reticulon-3 OS=Homo sapiens OX=9606 GN=RTN3 PE=1 SV=2	0.002	2.392	1	1	2	1	1032	112.5	4.96	4.31	1	0	125959.6094	NCDN only
Q5VUJ6	Leucine-rich repeat and calponin homology domain-containing protein 2 OS=Homo sapiens OX=9606 GN=LRCH2 PE=1 SV=2	0.002	2.88	1	1	2	1	765	84.5	6.55	0	1	0	43354.02734	NCDN only
Q9BW92	Threonine--tRNA ligase, mitochondrial OS=Homo sapiens OX=9606 GN=TARS2 PE=1 SV=1	0.002	3.066	1	1	2	1	718	81	7.3	4.15	1	0	69093.47656	NCDN only
Q14254	Flotillin-2 OS=Homo sapiens OX=9606 GN=FLOT2 PE=1 SV=2	0.005	1.901	2	1	2	1	428	47	5.25	2.1	1	0	48437.32813	NCDN only
P29590	Protein PML OS=Homo sapiens OX=9606 GN=PML PE=1 SV=3	0.002	2.953	1	1	2	1	882	97.5	6.21	4.03	1	0	41379.53516	NCDN only
Q8N6R0	eEF1A lysine and N-terminal methyltransferase OS=Homo sapiens OX=9606 GN=EEF1AKNMT PE=1 SV=1	0.002	2.108	1	1	2	1	699	78.7	6.73	1.83	1	0	47933.76172	NCDN only
P49768	Presenilin-1 OS=Homo sapiens OX=9606 GN=PSEN1 PE=1 SV=1	0.006	1.807	2	1	2	1	467	52.6	5.31	1.78	1	0	37028.99414	NCDN only
P54577	Tyrosine--tRNA ligase, cytoplasmic OS=Homo sapiens OX=9606 GN=YARS1 PE=1 SV=4	0.002	2.562	2	1	2	1	528	59.1	7.05	1.93	1	0	35872.72266	NCDN only
O15126	Secretory carrier-associated membrane protein 1 OS=Homo sapiens OX=9606 GN=SCAMP1 PE=1 SV=2	0	17	9	2	2	2	338	37.9	7.42	7.7	2	0	39226.82813	NCDN only
P30740	Leukocyte elastase inhibitor OS=Homo sapiens OX=9606 GN=SERPINB1 PE=1 SV=1	0.002	2.114	2	1	2	1	379	42.7	6.28	2.03	1	0	35063.4375	NCDN only
P12532	Creatine kinase U-type, mitochondrial OS=Homo sapiens OX=9606 GN=CKMT1A PE=1 SV=1	0.002	3.045	2	1	2	1	417	47	8.34	4.77	1	0	2068033.75	NCDN only
Q14671	Pumilio homolog 1 OS=Homo sapiens OX=9606 GN=PUM1 PE=1 SV=3	0	5.025	2	2	2	2	1186	126.4	6.84	1.96	2	0	192951.8359	NCDN only
Q92598	Heat shock protein 105 kDa OS=Homo sapiens OX=9606 GN=HSPH1 PE=1 SV=1	0	8.797	3	2	2	2	858	96.8	5.39	2.72	2	0	810097.25	NCDN only
Q13464	Rho-associated protein kinase 1 OS=Homo sapiens OX=9606 GN=ROCK1 PE=1 SV=1	0	4.493	1	2	2	2	1354	158.1	5.9	0	2	0	43498.20801	NCDN only
O43805	Sjoegren syndrome nuclear autoantigen 1 OS=Homo sapiens OX=9606 GN=SSNA1 PE=1 SV=2	0.007	1.772	6	1	2	1	119	13.6	5.38	3.98	1	0	121043.9766	NCDN only
P52294	Importin subunit alpha-5 OS=Homo sapiens OX=9606 GN=KPNA1 PE=1 SV=3	0	6.48	2	1	2	1	538	60.2	5.01	6.24	1	0	83438.5918	NCDN only
P30876	DNA-directed RNA polymerase II subunit RPB2 OS=Homo sapiens OX=9606 GN=POLR2B PE=1 SV=1	0.007	1.764	1	1	2	1	1174	133.8	6.87	0	1	0	26773.74414	NCDN only
Q9P289	Serine/threonine-protein kinase 26 OS=Homo sapiens OX=9606 GN=STK26 PE=1 SV=2	0.007	1.69	2	1	2	1	416	46.5	5.29	1.63	1	0	86424.63672	NCDN only
Q96HA1	Nuclear envelope pore membrane protein POM 121 OS=Homo sapiens OX=9606 GN=POM121 PE=1 SV=2	0.002	2.623	1	1	2	1	1249	127.6	10.56	2.42	1	0	41390.66797	NCDN only

Q9UQE7	Structural maintenance of chromosomes protein 3 OS=Homo sapiens OX=9606 GN=SMC3 PE=1 SV=2	0	4.647	1	2	2	2	1217	141.5	7.18	1.99	2	0	20683.53516	NCDN only
P53621	Coatomer subunit alpha OS=Homo sapiens OX=9606 GN=COPA PE=1 SV=2	0	4.523	1	2	2	2	1224	138.3	7.66	2.12	2	0	39572.05469	NCDN only
Q8N5K1	CDGSH iron-sulfur domain-containing protein 2 OS=Homo sapiens OX=9606 GN=CISD2 PE=1 SV=1	0.022	1.413	6	1	2	1	135	15.3	9.61	1.67	1	0	294200.3125	NCDN only
P62826	GTP-binding nuclear protein Ran OS=Homo sapiens OX=9606 GN=RAN PE=1 SV=3	0	3.959	5	1	2	1	216	24.4	7.49	3.02	1	0	1273259.25	NCDN only
O15270	Serine palmitoyltransferase 2 OS=Homo sapiens OX=9606 GN=SPTLC2 PE=1 SV=1	0	4.601	3	2	2	2	562	62.9	7.78	0	2	0	1019123.75	NCDN only
Q6A163	Keratin, type I cytoskeletal 39 OS=Homo sapiens OX=9606 GN=KRT39 PE=1 SV=2	0	3.787	2	1	2	1	491	55.6	5.26	4.21	1	0	163325.7031	NCDN only
Q96JH8	Ras-associating and dilute domain-containing protein OS=Homo sapiens OX=9606 GN=RADIL PE=1 SV=5	0.002	2.688	1	1	2	1	1075	117.4	7.09	2.05	1	0	51629.06641	NCDN only
Q9NRP0	Oligosaccharyltransferase complex subunit OSTC OS=Homo sapiens OX=9606 GN=OSTC PE=1 SV=1	0.011	1.545	5	1	2	1	149	16.8	9.13	1.76	1	0	87803.55469	NCDN only
P40937	Replication factor C subunit 5 OS=Homo sapiens OX=9606 GN=RFC5 PE=1 SV=1	0.002	2.38	2	1	2	1	340	38.5	7.2	4.23	1	0	69827.89063	NCDN only
P60602	Reactive oxygen species modulator 1 OS=Homo sapiens OX=9606 GN=ROMO1 PE=1 SV=1	0.008	1.633	10	1	2	1	79	8.2	9.33	0	1	0	308317.4688	NCDN only
O75439	Mitochondrial-processing peptidase subunit beta OS=Homo sapiens OX=9606 GN=PMPCB PE=1 SV=2	0	3.514	3	2	2	2	489	54.3	6.83	3.4	2	0	499177.625	NCDN only
Q8WUY8	N-acetyltransferase 14 OS=Homo sapiens OX=9606 GN=NAT14 PE=1 SV=1	0.007	1.726	4	1	2	1	206	21.6	10.74	1.75	1	0	20809.99023	NCDN only
Q5VW38	Protein GPR107 OS=Homo sapiens OX=9606 GN=GPR107 PE=1 SV=1	0	3.932	2	1	2	1	600	66.9	7.24	2.72	1	0	2237195.438	NCDN only
O00186	Syntaxin-binding protein 3 OS=Homo sapiens OX=9606 GN=STXBP3 PE=1 SV=2	0	3.755	2	2	2	2	592	67.7	7.8	1.99	2	0	48316.84766	NCDN only
P50416	Carnitine O-palmitoyltransferase 1, liver isoform OS=Homo sapiens OX=9606 GN=CPT1A PE=1 SV=2	0	4.559	2	2	2	2	773	88.3	8.65	4.08	2	0	179946.3906	NCDN only
P41250	Glycine--tRNA ligase OS=Homo sapiens OX=9606 GN=GARS1 PE=1 SV=3	0	3.74	2	2	2	2	739	83.1	7.03	0	2	0	17791.44141	NCDN only
Q7Z5L9	Interferon regulatory factor 2-binding protein 2 OS=Homo sapiens OX=9606 GN=IRF2BP2 PE=1 SV=2	0.004	1.987	1	1	2	1	587	61	8.69	4.04	1	0	107363.3984	NCDN only
Q08554	Desmocollin-1 OS=Homo sapiens OX=9606 GN=DSC1 PE=1 SV=2	0	10.147	3	2	2	2	894	99.9	5.43	5.44	2	0	591006	NCDN only
Q9H7C9	Mth938 domain-containing protein OS=Homo sapiens OX=9606 GN=AAMDC PE=1 SV=1	0.002	2.901	7	1	2	1	122	13.3	8.46	3.74	1	0	159687.5156	NCDN only
Q8TEB1	DDB1- and CUL4-associated factor 11 OS=Homo sapiens OX=9606 GN=DCAF11 PE=1 SV=1	0.007	1.721	1	1	2	1	546	61.6	6.4	0	1	0	45410.66797	NCDN only
Q86VI3	Ras GTPase-activating-like protein IQGAP3 OS=Homo sapiens OX=9606 GN=IQGAP3 PE=1 SV=2	0.002	2.098	1	1	2	1	1631	184.6	7.65	3.77	1	0	237444.5625	NCDN only
Q9H0X4	Protein FAM234A OS=Homo sapiens OX=9606 GN=FAM234A PE=1 SV=1	0.002	2.166	2	1	2	1	552	59.6	6.28	1.85	1	0	400024.25	NCDN only
Q96AV8	Transcription factor E2F7 OS=Homo sapiens OX=9606 GN=E2F7 PE=1 SV=3	0	4.807	1	1	2	1	911	99.8	8.16	4.5	1	0	72114.5918	NCDN only
Q9NWV8	BRISC and BRCA1-A complex member 1 OS=Homo sapiens OX=9606 GN=BABAM1 PE=1 SV=1	0	3.653	3	1	2	1	329	36.5	4.64	2.06	1	0	26002.84375	NCDN only
P09622	Dihydrolipoyl dehydrogenase, mitochondrial OS=Homo sapiens OX=9606 GN=DLD PE=1 SV=2	0	5.039	3	2	2	2	509	54.1	7.85	3.61	2	0	103003.3672	NCDN only
Q13428	Treacle protein OS=Homo sapiens OX=9606 GN=TCOF1 PE=1 SV=3	0	8.072	1	2	2	2	1488	152	9.04	3.98	2	0	89965.82422	NCDN only
Q6ZMG9	Ceramide synthase 6 OS=Homo sapiens OX=9606 GN=CERS6 PE=1 SV=1	0.002	2.977	3	1	1	1	384	44.9	7.65	2.17	1	0	147279.0469	NCDN only
Q06124	Tyrosine-protein phosphatase non-receptor type 11 OS=Homo sapiens OX=9606 GN=PTPN11 PE=1 SV=3	0.007	1.734	1	1	1	1	593	68	7.3	0	1	0	21281.70703	NCDN only
P35658	Nuclear pore complex protein Nup214 OS=Homo sapiens OX=9606 GN=NUP214 PE=1 SV=2	0.002	2.615	0	1	1	1	2090	213.5	7.47	0	1	0	29622.45703	NCDN only
Q96KB5	Lymphokine-activated killer T-cell-originated protein kinase OS=Homo sapiens OX=9606 GN=PBK PE=1 SV=3	0.002	2.114	2	1	1	1	322	36.1	5.12	0	1	0	40885.69141	NCDN only
Q8N2G8	GH3 domain-containing protein OS=Homo sapiens OX=9606 GN=GHDC PE=1 SV=2	0.002	2.059	2	1	1	1	530	57.5	7.88	1.63	1	0	605160.4375	NCDN only
Q9H3U1	Protein unc-45 homolog A OS=Homo sapiens OX=9606 GN=UNC45A PE=1 SV=1	0.002	2.206	1	1	1	1	944	103	6.07	1.88	1	0	49242.61719	NCDN only
Q8IZH2	5'-3' exoribonuclease 1 OS=Homo sapiens OX=9606 GN=XRN1 PE=1 SV=1	0	3.543	1	1	1	1	1706	194	7.21	0	1	0	36862.57422	NCDN only
P98194	Calcium-transporting ATPase type 2C member 1 OS=Homo sapiens OX=9606 GN=ATP2C1 PE=1 SV=3	0.004	2.031	1	1	1	1	919	100.5	6.74	1.66	1	0	45817.75391	NCDN only
P49257	Protein ERGIC-53 OS=Homo sapiens OX=9606 GN=LMAN1 PE=1 SV=2	0.002	2.512	2	1	1	1	510	57.5	6.77	0	1	0	28240.43945	NCDN only
O15182	Centrin-3 OS=Homo sapiens OX=9606 GN=CETN3 PE=1 SV=2	0.002	2.833	5	1	1	1	167	19.5	4.74	0	1	0	20314.0625	NCDN only
P02765	Alpha-2-HS-glycoprotein OS=Homo sapiens OX=9606 GN=AHSG PE=1 SV=2	0	7.393	3	1	1	1	367	39.3	5.72	2.38	1	0	28225.82227	NCDN only
Q5VT06	Centrosome-associated protein 350 OS=Homo sapiens OX=9606 GN=CEP350 PE=1 SV=1	0.002	2.146	0	1	1	1	3117	350.7	6.33	1.75	1	0	17668.54297	NCDN only
P04062	Lysosomal acid glucosylceramidase OS=Homo sapiens OX=9606 GN=GBA PE=1 SV=3	0.002	3.112	1	1	1	1	536	59.7	7.61	2.28	1	0	60665.38672	NCDN only
Q86T03	Type 1 phosphatidylinositol 4,5-bisphosphate 4-phosphatase OS=Homo sapiens OX=9606 GN=PIP4P1 PE=1 SV=1	0	12.276	8	1	1	1	277	29.5	8.91	4.6	1	0	1037975	NCDN only
Q99459	Cell division cycle 5-like protein OS=Homo sapiens OX=9606 GN=CDC5L PE=1 SV=2	0.005	1.975	1	1	1	1	802	92.2	8.18	2.02	1	0	40513.24219	NCDN only
Q8N6I4	Transmembrane protein 251 OS=Homo sapiens OX=9606 GN=TMEM251 PE=2 SV=4	0.005	1.828	4	1	1	1	163	18.7	8.19	1.83	1	0	31499.19922	NCDN only
Q14964	Ras-related protein Rab-39A OS=Homo sapiens OX=9606 GN=RAB39A PE=1 SV=2	0.005	1.934	3	1	1	1	217	25	7.65	0	1	0	101633.0938	NCDN only
P41227	N-alpha-acetyltransferase 10 OS=Homo sapiens OX=9606 GN=NAA10 PE=1 SV=1	0.002	2.277	4	1	1	1	235	26.4	5.64	0	1	0	276568.875	NCDN only
P49792	E3 SUMO-protein ligase RanBP2 OS=Homo sapiens OX=9606 GN=RANBP2 PE=1 SV=2	0.002	2.898	0	1	1	1	3224	358	6.2	1.82	1	0	59041.70703	NCDN only
Q16514	Transcription initiation factor TFIID subunit 12 OS=Homo sapiens OX=9606 GN=TAF12 PE=1 SV=1	0.011	1.553	6	1	1	1	161	17.9	7.99	0	1	0	42044.60938	NCDN only
Q9Y5N6	Origin recognition complex subunit 6 OS=Homo sapiens OX=9606 GN=ORC6 PE=1 SV=1	0	3.529	4	1	1	1	252	28.1	8.66	2.33	1	0	39128.48438	NCDN only
P19367	Hexokinase-1 OS=Homo sapiens OX=9606 GN=HK1 PE=1 SV=3	0.002	2.864	1	1	1	1	917	102.4	6.8	2.02	1	0	36788.78906	NCDN only
P35998	26S proteasome regulatory subunit 7 OS=Homo sapiens OX=9606 GN=PSMC2 PE=1 SV=3	0.001	3.272	2	1	1	1	433	48.6	5.95	0	1	0	29582.69141	NCDN only
Q04837	Single-stranded DNA-binding protein, mitochondrial OS=Homo sapiens OX=9606 GN=SSBP1 PE=1 SV=1	0.007	1.716	5	1	1	1	148	17.2	9.6	1.91	1	0	53594.60156	NCDN only
P17948	Vascular endothelial growth factor receptor 1 OS=Homo sapiens OX=9606 GN=FLT1 PE=1 SV=2	0	4.134	1	1	1	1	1338	150.7	8.38	1.73	1	0	3751268.25	NCDN only
P11908	Ribose-phosphate pyrophosphokinase 2 OS=Homo sapiens OX=9606 GN=PRPS2 PE=1 SV=2	0.002	2.338	3	1	1	1	318	34.7	6.61	1.85	1	0	38796.70313	NCDN only
P10155	60 kDa SS-A/Ro ribonucleoprotein OS=Homo sapiens OX=9606 GN=RO60 PE=1 SV=2	0.001	3.464	1	1	1	1	538	60.6	8.03	2.06	1	0	69656.96875	NCDN only
Q99848	Probable rRNA-processing protein EBP2 OS=Homo sapiens OX=9606 GN=EBNA1BP2 PE=1 SV=2	0.002	2.692	3	1	1	1	306	34.8	10.1	1.94	1	0	34955.64063	NCDN only
Q8N163	Cell cycle and apoptosis regulator protein 2 OS=Homo sapiens OX=9606 GN=CCAR2 PE=1 SV=2	0.002	2.445	1	1	1	1	923	102.8	5.22	1.65	1	0	25768.41992	NCDN only
Q9UBE0	SUMO-activating enzyme subunit 1 OS=Homo sapiens OX=9606 GN=SAE1 PE=1 SV=1	0.002	2.765	4	1	1	1	346	38.4	5.3	0	1	0	37707.76953	NCDN only
O00429	Dynamin-1-like protein OS=Homo sapiens OX=9606 GN=DNM1L PE=1 SV=2	0.008	1.585	1	1	1	1	736	81.8	6.81	0	1	0	48936.6875	NCDN only
Q9UHI6	Probable ATP-dependent RNA helicase DDX20 OS=Homo sapiens OX=9606 GN=DDX20 PE=1 SV=2	0.002	2.702	1	1	1	1	824	92.2	6.95	0	1	0	51795.44141	NCDN only
Q8WVP7	Limb region 1 protein homolog OS=Homo sapiens OX=9606 GN=LMBR1 PE=1 SV=1	0.002	3.003	2	1	1	1	490	55.1	5.94	0	1	0	26707.8418	NCDN only
P11182	Lipoamide acyltransferase component of branched-chain alpha-keto acid dehydrogenase complex, mitochondrial OS=Homo sapiens OX=9606 GN=DBT PE=1 SV=3	0	3.971	2	1	1	1	482	53.5	8.51	2.23	1	0	25983.54883	NCDN only
Q96K76	Ubiquitin carboxyl-terminal hydrolase 47 OS=Homo sapiens OX=9606 GN=USP47 PE=1 SV=3	0.022	1.423	1	1	1	1	1375	157.2	5.08	1.91	1	0	61464.57422	NCDN only
Q5JPE7	Nodal modulator 2 OS=Homo sapiens OX=9606 GN=NOMO2 PE=1 SV=1	0.002	2.531	1	1	1	1	1267	139.4	5.76	1.71	1	0	42662.92188	NCDN only
Q0VDF9	Heat shock 70 kDa protein 14 OS=Homo sapiens OX=9606 GN=HSPA14 PE=1 SV=1	0.005	1.888	2	1	1	1	509	54.8	5.59	1.7	1	0	31321.97461	NCDN only
P00403	Cytochrome c oxidase subunit 2 OS=Homo sapiens OX=9606 GN=MT-CO2 PE=1 SV=1	0	4.278	4	1	1	1	227	25.5	4.82	2.77	1	0	1751765.75	NCDN only
Q8N2U9	Solute carrier family 66 member 2 OS=Homo sapiens OX=9606 GN=SLC66A2 PE=1 SV=1	0.002	2.703	3	1	1	1	271	30.5	8.18	2.08	1	0	50690.99219	NCDN only
Q92922	SWI/SNF complex subunit SMARCC1 OS=Homo sapiens OX=9606 GN=SMARCC1 PE=1 SV=3	0.013	1.487	1	1	1	1	1105	122.8	5.76	0	1	0	28645.36133	NCDN only
Q9UMZ2	Synergin gamma OS=Homo sapiens OX=9606 GN=SYNRG PE=1 SV=2	0.005	1.85	1	1	1	1	1314	140.6	5.03	0	1	0	30450.47656	NCDN only
P59768	Guanine nucleotide-binding protein G(I)/G(S)/G(O) subunit gamma-2 OS=Homo sapiens OX=9606 GN=GNG2 PE=1 SV=2	0.005	1.944	18	1	1	1	71	7.8	7.99	1.6	1	0	23069.89648	NCDN only
Q8N511	Transmembrane protein 199 OS=Homo sapiens OX=9606 GN=TMEM199 PE=1 SV=1	0.007	1.773	3	1	1	1	208	23.1	9.01	1.68	1	0	65033.81641	NCDN only
Q5SQN1	Synaptosomal-associated protein 47 OS=Homo sapiens OX=9606 GN=SNAP47 PE=1 SV=3	0.007	1.71	2	1	1	1	464	52.5	8.69	0	1	0	20184.76367	NCDN only
Q9NTJ3	Structural maintenance of chromosomes protein 4 OS=Homo sapiens OX=9606 GN=SMC4 PE=1 SV=2	0.001	3.283	1	1	1	1	1288	147.1	6.79	0	1	0	38897.30859	NCDN only
Q00587	Cdc42 effector protein 1 OS=Homo sapiens OX=9606 GN=CDC42EP1 PE=1 SV=1	0.002	2.567	2	1	1	1	391	40.3	7.15	2.53	1	0	96180.65625	NCDN only
Q9BRR6	ADP-dependent glucokinase OS=Homo sapiens OX=9606 GN=ADPGK PE=1 SV=1	0.002	2.538	2	1	1	1	497	54.1	6.2	2.01	1	0	230650.5156	NCDN only
Q9BXB4	Oxysterol-binding protein-related protein 11 OS=Homo sapiens OX=9606 GN=OSBPL11 PE=1 SV=2	0.005	1.894	1	1	1	1	747	83.6	7.06	1.73	1	0	20249.27539	NCDN only
P30041	Peroxiredoxin-6 OS=Homo sapiens OX=9606 GN=PRDX6 PE=1 SV=3	0	4.042	5	1	1	1	224	25	6.38	3.09	1	0	2635461.75	NCDN only
Q9UPT5	Exocyst complex component 7 OS=Homo sapiens OX=9606 GN=EXOC7 PE=1 SV=3	0.005	1.842	1	1	1	1	735	83.3	6.79	1.74	1	0	31764.07617	NCDN only
Q70UQ0	Inhibitor of nuclear factor kappa-B kinase-interacting protein OS=Homo sapiens OX=9606 GN=IKBIP PE=1 SV=1	0	4.496	3	1	1	1	350	39.3	9.17	2.02	1	0	48724.50391	NCDN only
Q9NWU2	Glucose-induced degradation protein 8 homolog OS=Homo sapiens OX=9606 GN=GID8 PE=1 SV=1	0.007	1.695	3	1	1	1	228	26.7	4.97	0	1	0	32017.31836	NCDN only
P11234	Ras-related protein Ral-B OS=Homo sapiens OX=9606 GN=RALB PE=1 SV=1	0.006	1.796	4	1	1	1	206	23.4	6.62	0	1	0	37975.69922	NCDN only
O00258	Guided entry of tail-anchored proteins factor 1 OS=Homo sapiens OX=9606 GN=GET1 PE=1 SV=2	0.005	1.917	4	1	1	1	174	19.8	9.67	0	1	0	59293.62891	NCDN only
Q8NCH0	Carbohydrate sulfotransferase 14 OS=Homo sapiens OX=9606 GN=CHST14 PE=1 SV=2	0	4.457	2	1	1	1	376	43	9.48	2.48	1	0	36298.72656	NCDN only
Q9H8H0	Nucleolar protein 11 OS=Homo sapiens OX=9606 GN=NOL11 PE=1 SV=1	0.002	2.619	1	1	1	1	719	81.1	6.07	1.75	1	0	24824.08594	NCDN only
Q9P2X0	Dolichol-phosphate mannosyltransferase subunit 3 OS=Homo sapiens OX=9606 GN=DPM3 PE=1 SV=2	0	4.053	11	1	1	1	92	10.1	5.94	3.19	1	0	1161366.5	NCDN only
O95202	Mitochondrial proton/calcium exchanger protein OS=Homo sapiens OX=9606 GN=LETM1 PE=1 SV=1	0.002	2.189	2	1	1	1	739	83.3	6.7	0	1	0	25739.83594	NCDN only
Q6NXE6	Armadillo repeat-containing protein 6 OS=Homo sapiens OX=9606 GN=ARMC6 PE=1 SV=2	0.006	1.796	1	1	1	1	501	54.1	6.24	1.74	1	0	54166.46484	NCDN only
Q13310	Polyadenylate-binding protein 4 OS=Homo sapiens OX=9606 GN=PABPC4 PE=1 SV=1	0.002	2.227	1	1	1	1	644	70.7	9.26	1.69	1	0	49679.34766	NCDN only
Q15061	WD repeat-containing protein 43 OS=Homo sapiens OX=9606 GN=WDR43 PE=1 SV=3	0.008	1.581	1	1	1	1	677	74.8	5.57	0	1	0	75814.71094	NCDN only
Q9H1X3	DnaJ homolog subfamily C member 25 OS=Homo sapiens OX=9606 GN=DNAJC25 PE=1 SV=1	0.002	2.545	2	1	1	1	360	42.4	9.13	2.53	1	0	139766	NCDN only
P46734	Dual specificity mitogen-activated protein kinase kinase 3 OS=Homo sapiens OX=9606 GN=MAP2K3 PE=1 SV=2	0.002	3.005	2	1	1	1	347	39.3	7.43	1.77	1	0	41901.72266	NCDN only
P26232	Catenin alpha-2 OS=Homo sapiens OX=9606 GN=CTNNA2 PE=1 SV=5	0.002	2.807	1	1	1	1	953	105.2	5.71	1.96	1	0	70570.75	NCDN only
O60832	H/ACA ribonucleoprotein complex subunit DKC1 OS=Homo sapiens OX=9606 GN=DKC1 PE=1 SV=3	0.002	2.646	2	1	1	1	514	57.6	9.42	1.74	1	0	22524.58203	NCDN only
Q96BQ5	Coiled-coil domain-containing protein 127 OS=Homo sapiens OX=9606 GN=CCDC127 PE=1 SV=1	0.002	2.375	3	1	1	1	260	30.8	9.2	0	1	0	75982.28125	NCDN only
O75947	ATP synthase subunit d, mitochondrial OS=Homo sapiens OX=9606 GN=ATP5PD PE=1 SV=3	0.008	1.647	4	1	1	1	161	18.5	5.3	1.61	1	0	23438.87695	NCDN only
P00558	Phosphoglycerate kinase 1 OS=Homo sapiens OX=9606 GN=PGK1 PE=1 SV=3	0.007	1.76	2	1	1	1	417	44.6	8.1	1.74	1	0	30661.41211	NCDN only
P56589	Peroxisomal biogenesis factor 3 OS=Homo sapiens OX=9606 GN=PEX3 PE=1 SV=1	0	4.921	3	1	1	1	373	42.1	8.15	2.88	1	0	408607.9688	NCDN only
Q9BVG9	Phosphatidylserine synthase 2 OS=Homo sapiens OX=9606 GN=PTDSS2 PE=1 SV=1	0	6.091	5	1	1	1	487	56.2	6.25	4.23	1	0	113975.4297	NCDN only
P06702	Protein S100-A9 OS=Homo sapiens OX=9606 GN=S100A9 PE=1 SV=1	0	5.493	11	1	1	1	114	13.2	6.13	2.75	1	0	2126459	NCDN only
P33993	DNA replication licensing factor MCM7 OS=Homo sapiens OX=9606 GN=MCM7 PE=1 SV=4	0.002	2.386	1	1	1	1	719	81.3	6.46	0	1	0	38358.76172	NCDN only
Q04323	UBX domain-containing protein 1 OS=Homo sapiens OX=9606 GN=UBXN1 PE=1 SV=2	0.001	3.294	3	1	1	1	297	33.3	5.25	2.04	1	0	211300.4063	NCDN only
Q13813	Spectrin alpha chain, non-erythrocytic 1 OS=Homo sapiens OX=9606 GN=SPTAN1 PE=1 SV=3	0.022	1.406	0	1	1	1	2472	284.4	5.35	0	1	0	26317.76563	NCDN only
Q01804	OTU domain-containing protein 4 OS=Homo sapiens OX=9606 GN=OTUD4 PE=1 SV=4	0.008	1.646	1	1	1	1	1114	124	6.71	0	1	0	89907.4375	NCDN only
Q9Y241	HIG1 domain family member 1A, mitochondrial OS=Homo sapiens OX=9606 GN=HIGD1A PE=1 SV=1	0	5.318	20	1	1	1	93	10.1	9.79	3.45	1	0	149345.4688	NCDN only
Q8N0U8	Vitamin K epoxide reductase complex subunit 1-like protein 1 OS=Homo sapiens OX=9606 GN=VKORC1L1 PE=1 SV=2	0.006	1.787	5	1	1	1	176	19.8	9.13	2.04	1	0	135827.6719	NCDN only
O94906	Pre-mRNA-processing factor 6 OS=Homo sapiens OX=9606 GN=PRPF6 PE=1 SV=1	0.007	1.715	1	1	1	1	941	106.9	8.25	1.64	1	0	96885.54688	NCDN only
Q8IU81	Interferon regulatory factor 2-binding protein 1 OS=Homo sapiens OX=9606 GN=IRF2BP1 PE=1 SV=1	0.003	2.045	1	1	1	1	584	61.6	8.18	0	1	0	24103.18164	NCDN only
Q9UBB4	Ataxin-10 OS=Homo sapiens OX=9606 GN=ATXN10 PE=1 SV=1	0.022	1.424	2	1	1	1	475	53.5	5.25	1.63	1	0	44706.61328	NCDN only
P40939	Trifunctional enzyme subunit alpha, mitochondrial OS=Homo sapiens OX=9606 GN=HADHA PE=1 SV=2	0	4.642	2	1	1	1	763	82.9	9.04	3.7	1	0	505115.0313	NCDN only
Q9NRZ7	1-acyl-sn-glycerol-3-phosphate acyltransferase gamma OS=Homo sapiens OX=9606 GN=AGPAT3 PE=1 SV=1	0.002	2.955	3	1	1	1	376	43.4	8.72	1.63	1	0	46574.97266	NCDN only
Q8WUJ1	Neuferricin OS=Homo sapiens OX=9606 GN=CYB5D2 PE=1 SV=1	0.011	1.52	3	1	1	1	264	28.7	8.4	0	1	0	49581.68359	NCDN only
Q5SZJ8	BEN domain-containing protein 6 OS=Homo sapiens OX=9606 GN=BEND6 PE=1 SV=2	0.007	1.75	3	1	1	1	279	31.2	8.35	1.81	1	0	219958.4531	NCDN only
Q7KYR7	Butyrophilin subfamily 2 member A1 OS=Homo sapiens OX=9606 GN=BTN2A1 PE=1 SV=3	0.002	2.51	2	1	1	1	527	59.6	6.48	0	1	0	24405.1582	NCDN only
Q9NRG1	Phosphoribosyltransferase domain-containing protein 1 OS=Homo sapiens OX=9606 GN=PRTFDC1 PE=1 SV=1	0.013	1.5	3	1	1	1	225	25.7	6.15	1.8	1	0	974051.5625	NCDN only
Q6NZ67	Mitotic-spindle organizing protein 2B OS=Homo sapiens OX=9606 GN=MZT2B PE=1 SV=1	0	8.765	14	1	1	1	158	16.2	10.15	2.83	1	0	49261.72656	NCDN only
P62424	60S ribosomal protein L7a OS=Homo sapiens OX=9606 GN=RPL7A PE=1 SV=2	0.002	2.135	3	1	1	1	266	30	10.61	1.64	1	0	35822.71484	NCDN only
O94905	Erlin-2 OS=Homo sapiens OX=9606 GN=ERLIN2 PE=1 SV=1	0.007	1.72	2	1	1	1	339	37.8	5.62	1.77	1	0	229753.0781	NCDN only
P30101	Protein disulfide-isomerase A3 OS=Homo sapiens OX=9606 GN=PDIA3 PE=1 SV=4	0.022	1.414	2	1	1	1	505	56.7	6.35	1.78	1	0	59995.21875	NCDN only
O94776	Metastasis-associated protein MTA2 OS=Homo sapiens OX=9606 GN=MTA2 PE=1 SV=1	0	3.605	1	1	1	1	668	75	9.66	2.86	1	0	388900.6875	NCDN only
Q07666	KH domain-containing, RNA-binding, signal transduction-associated protein 1 OS=Homo sapiens OX=9606 GN=KHDRBS1 PE=1 SV=1	0	6.634	3	1	1	1	443	48.2	8.66	3.24	1	0	73019.69531	NCDN only
P54136	Arginine--tRNA ligase, cytoplasmic OS=Homo sapiens OX=9606 GN=RARS1 PE=1 SV=2	0.002	2.957	1	1	1	1	660	75.3	6.68	0	1	0	49300.15234	NCDN only
Q15907	Ras-related protein Rab-11B OS=Homo sapiens OX=9606 GN=RAB11B PE=1 SV=4	0.005	1.834	4	1	1	1	218	24.5	5.94	1.63	1	0	77568.08594	NCDN only
A6NDU8	UPF0600 protein C5orf51 OS=Homo sapiens OX=9606 GN=C5orf51 PE=1 SV=1	0	3.646	3	1	1	1	294	33.6	5.26	2.59	1	0	143177.0938	NCDN only
O95159	Zinc finger protein-like 1 OS=Homo sapiens OX=9606 GN=ZFPL1 PE=1 SV=2	0	8.652	6	1	1	1	310	34.1	8.07	3.58	1	0	788392.4375	NCDN only
Q92747	Actin-related protein 2/3 complex subunit 1A OS=Homo sapiens OX=9606 GN=ARPC1A PE=1 SV=2	0.002	2.196	2	1	1	1	370	41.5	8.18	0	1	0	283100.7813	NCDN only
Q9UKK9	ADP-sugar pyrophosphatase OS=Homo sapiens OX=9606 GN=NUDT5 PE=1 SV=1	0.002	2.218	4	1	1	1	219	24.3	4.94	0	1	0	58337.87891	NCDN only
P30273	High affinity immunoglobulin epsilon receptor subunit gamma OS=Homo sapiens OX=9606 GN=FCER1G PE=1 SV=1	0.011	1.523	9	1	1	1	86	9.7	7.12	1.76	1	0	19547.26563	NCDN only
P12081	Histidine--tRNA ligase, cytoplasmic OS=Homo sapiens OX=9606 GN=HARS1 PE=1 SV=2	0.005	1.902	1	1	1	1	509	57.4	5.88	1.77	1	0	30279.02148	NCDN only

Q8WWM7	Ataxin-2-like protein OS=Homo sapiens OX=9606 GN=ATXN2L PE=1 SV=2	0.002	2.551	1	1	1	1	1075	113.3	8.59	1.76	1	0	21732.38867		NCDN only
P13804	Electron transfer flavoprotein subunit alpha, mitochondrial OS=Homo sapiens OX=9606 GN=ETFA PE=1 SV=1	0.006	1.8	2	1	1	1	333	35.1	8.38	1.67	1	0	80312.71094		NCDN only
Q9UJS0	Calcium-binding mitochondrial carrier protein Aralar2 OS=Homo sapiens OX=9606 GN=SLC25A13 PE=1 SV=2	0.007	1.775	1	1	1	1	675	74.1	8.62	1.76	1	0	3882827.75		NCDN only
P35609	Alpha-actinin-2 OS=Homo sapiens OX=9606 GN=ACTN2 PE=1 SV=1	0.002	3.062	1	1	1	1	894	103.8	5.45	2.27	1	0	747924.1875		NCDN only
O43707	Alpha-actinin-4 OS=Homo sapiens OX=9606 GN=ACTN4 PE=1 SV=2	0.002	2.139	1	1	1	1	911	104.8	5.44	2.11	1	0	36795.78516		NCDN only
Q5JXB2	Putative ubiquitin-conjugating enzyme E2 N-like OS=Homo sapiens OX=9606 GN=UBE2NL PE=1 SV=1	0	4.234	7	1	1	1	153	17.4	5.92	3.14	1	0	561851.875		NCDN only
Q9Y6C9	Mitochondrial carrier homolog 2 OS=Homo sapiens OX=9606 GN=MTCH2 PE=1 SV=1	0.002	2.452	3	1	1	1	303	33.3	7.97	2.2	1	0	887075.25		NCDN only
O75419	Cell division control protein 45 homolog OS=Homo sapiens OX=9606 GN=CDC45 PE=1 SV=1	0.002	2.398	2	1	1	1	566	65.5	5.53	0	1	0	41601.85547		NCDN only
Q9NUU6	Inactive ubiquitin thioesterase OTULINL OS=Homo sapiens OX=9606 GN=OTULINL PE=1 SV=1	0.011	1.515	2	1	1	1	356	42.2	9.29	0	1	0	32842.03516		NCDN only
P50570	Dynamin-2 OS=Homo sapiens OX=9606 GN=DNM2 PE=1 SV=2	0.007	1.768	1	1	1	1	870	98	7.44	2.05	1	0	41115.37109		NCDN only
P35222	Catenin beta-1 OS=Homo sapiens OX=9606 GN=CTNNB1 PE=1 SV=1	0.008	1.673	2	1	1	1	781	85.4	5.86	0	1	0	39846.32813		NCDN only
Q9BTX1	Nucleoporin NDC1 OS=Homo sapiens OX=9606 GN=NDC1 PE=1 SV=2	0.005	1.946	1	1	1	1	674	76.3	9.09	1.71	1	0	48494.23438		NCDN only
P39210	Protein Mpv17 OS=Homo sapiens OX=9606 GN=MPV17 PE=1 SV=1	0.022	1.429	4	1	1	1	176	19.7	9.47	0	1	0	118246.9766		NCDN only
Q6PML9	Zinc transporter 9 OS=Homo sapiens OX=9606 GN=SLC30A9 PE=1 SV=1	0.011	1.518	1	1	1	1	568	63.5	8.32	0	1	0	18874.59766		NCDN only
Q5H9R7	Serine/threonine-protein phosphatase 6 regulatory subunit 3 OS=Homo sapiens OX=9606 GN=PPP6R3 PE=1 SV=2	0.005	1.87	1	1	1	1	873	97.6	4.6	1.82	1	0	46117.10156		NCDN only
P49406	39S ribosomal protein L19, mitochondrial OS=Homo sapiens OX=9606 GN=MRPL19 PE=1 SV=2	0.02	1.433	2	1	1	1	292	33.5	9.5	0	1	0	25611.24414		NCDN only
P41252	Isoleucine--tRNA ligase, cytoplasmic OS=Homo sapiens OX=9606 GN=IARS1 PE=1 SV=2	0.005	1.821	1	1	1	1	1262	144.4	6.15	1.93	1	0	159861.5625		NCDN only
O14975	Very long-chain acyl-CoA synthetase OS=Homo sapiens OX=9606 GN=SLC27A2 PE=1 SV=2	0.014	1.484	1	1	1	1	620	70.3	8.51	0	1	0	163029.7813		NCDN only
P30260	Cell division cycle protein 27 homolog OS=Homo sapiens OX=9606 GN=CDC27 PE=1 SV=2	0.002	3.123	1	1	1	1	824	91.8	7.02	1.85	1	0	58575.73828		NCDN only
P02794	Ferritin heavy chain OS=Homo sapiens OX=9606 GN=FTH1 PE=1 SV=2	0.002	2.861	5	1	1	1	183	21.2	5.55	2.29	1	0	25526.6875		NCDN only
P02788	Lactotransferrin OS=Homo sapiens OX=9606 GN=LTF PE=1 SV=6	0.002	2.202	1	1	1	1	710	78.1	8.12	0	1	0	1493133		NCDN only
P43686	26S proteasome regulatory subunit 6B OS=Homo sapiens OX=9606 GN=PSMC4 PE=1 SV=2	0.007	1.77	2	1	1	1	418	47.3	5.21	0	1	0	18612.43359		NCDN only
Q9Y519	Transmembrane protein 184B OS=Homo sapiens OX=9606 GN=TMEM184B PE=1 SV=2	0.005	1.873	2	1	1	1	407	45.5	6.89	1.64	1	0	52494.14063		NCDN only
P06737	Glycogen phosphorylase, liver form OS=Homo sapiens OX=9606 GN=PYGL PE=1 SV=4	0.011	1.559	1	1	1	1	847	97.1	7.17	0	1	0	135000.875		NCDN only
Q7Z7E8	Ubiquitin-conjugating enzyme E2 Q1 OS=Homo sapiens OX=9606 GN=UBE2Q1 PE=1 SV=1	0.009	1.571	2	1	1	1	422	46.1	5.1	1.84	1	0	70409.73438		NCDN only
Q8N0X7	Spartin OS=Homo sapiens OX=9606 GN=SPART PE=1 SV=1	0	4.96	2	1	1	1	666	72.8	5.91	2.82	1	0	28036.95117		NCDN only
Q8IUC8	Polypeptide N-acetylgalactosaminyltransferase 13 OS=Homo sapiens OX=9606 GN=GALNT13 PE=2 SV=2	0.002	2.117	2	1	1	1	556	64	6.83	0	1	0	373443.9375		NCDN only
O75083	WD repeat-containing protein 1 OS=Homo sapiens OX=9606 GN=WDR1 PE=1 SV=4	0.007	1.737	1	1	1	1	606	66.2	6.65	1.83	1	0	22987.03711		NCDN only
Q9NVE7	4'-phosphopantetheine phosphatase OS=Homo sapiens OX=9606 GN=PANK4 PE=1 SV=1	0.005	1.886	1	1	1	1	773	85.9	6.28	1.61	1	0	33964.46875		NCDN only
Q14152	Eukaryotic translation initiation factor 3 subunit A OS=Homo sapiens OX=9606 GN=EIF3A PE=1 SV=1	0.005	1.91	1	1	1	1	1382	166.5	6.79	0	1	0	61348.73828		NCDN only
Q9NYU2	UDP-glucose:glycoprotein glucosyltransferase 1 OS=Homo sapiens OX=9606 GN=UGGT1 PE=1 SV=3	0.002	2.057	1	1	1	1	1555	177.1	5.63	1.7	1	0	39517.71484		NCDN only
Q14839	Chromodomain-helicase-DNA-binding protein 4 OS=Homo sapiens OX=9606 GN=CHD4 PE=1 SV=2	0.022	1.427	1	1	1	1	1912	217.9	5.86	0	1	0	33464.07031		NCDN only
P40123	Adenylyl cyclase-associated protein 2 OS=Homo sapiens OX=9606 GN=CAP2 PE=1 SV=1	0.005	1.83	2	1	1	1	477	52.8	6.37	0	1	0	16939.25195		NCDN only
Q9UH99	SUN domain-containing protein 2 OS=Homo sapiens OX=9606 GN=SUN2 PE=1 SV=3	0.005	1.942	1	1	1	1	717	80.3	6.73	1.78	1	0	37978.16406		NCDN only
O60256	Phosphoribosyl pyrophosphate synthase-associated protein 2 OS=Homo sapiens OX=9606 GN=PRPSAP2 PE=1 SV=1	0.005	1.933	2	1	1	1	369	40.9	7.44	1.91	1	0	69387.9375		NCDN only
Q14667	Protein KIAA0100 OS=Homo sapiens OX=9606 GN=KIAA0100 PE=1 SV=3	0.002	2.903	0	1	1	1	2235	253.5	7.14	2.05	1	0	33004.67969		NCDN only
P23193	Transcription elongation factor A protein 1 OS=Homo sapiens OX=9606 GN=TCEA1 PE=1 SV=2	0.005	1.828	3	1	1	1	301	33.9	8.38	0	1	0	43942.57031		NCDN only
Q9Y2C4	Nuclease EXOG, mitochondrial OS=Homo sapiens OX=9606 GN=EXOG PE=1 SV=2	0.002	2.262	3	1	1	1	368	41.1	8.27	0	1	0	462940.375		NCDN only
A0A024RBG1	Diphosphoinositol polyphosphate phosphohydrolase NUDT4B OS=Homo sapiens OX=9606 GN=NUDT4B PE=3 SV=1	0.002	3.045	5	1	1	1	181	20.4	6.35	1.75	1	0	46053.20703		NCDN only
Q92797	Symplekin OS=Homo sapiens OX=9606 GN=SYMPK PE=1 SV=2	0.008	1.583	1	1	1	1	1274	141.1	6.13	0	1	0	31101.53711		NCDN only
Q08211	ATP-dependent RNA helicase A OS=Homo sapiens OX=9606 GN=DHX9 PE=1 SV=4	0.011	1.546	1	1	1	1	1270	140.9	6.84	0	1	0	24717.94922		NCDN only
O15258	Protein RER1 OS=Homo sapiens OX=9606 GN=RER1 PE=1 SV=1	0	12.132	10	1	1	1	196	22.9	9.54	4.89	1	0	4399917.5		NCDN only
Q8IYI6	Exocyst complex component 8 OS=Homo sapiens OX=9606 GN=EXOC8 PE=1 SV=2	0.005	1.928	1	1	1	1	725	81.7	5.49	0	1	0	19563.66016		NCDN only
Q13564	NEDD8-activating enzyme E1 regulatory subunit OS=Homo sapiens OX=9606 GN=NAE1 PE=1 SV=1	0.007	1.754	2	1	1	1	534	60.2	5.4	1.69	1	0	26550.49805		NCDN only
Q9Y5B9	FACT complex subunit SPT16 OS=Homo sapiens OX=9606 GN=SUPT16H PE=1 SV=1	0.002	2.249	1	1	1	1	1047	119.8	5.66	2.03	1	0	34406.30078		NCDN only
Q9UPY5	Cystine/glutamate transporter OS=Homo sapiens OX=9606 GN=SLC7A11 PE=1 SV=1	0.002	2.829	2	1	1	1	501	55.4	9.19	2.16	1	0	39141.05469		NCDN only
Q5HYI8	Rab-like protein 3 OS=Homo sapiens OX=9606 GN=RABL3 PE=1 SV=1	0	4.208	5	1	1	1	236	26.4	7.11	2.36	1	0	301363.7188		NCDN only
Q9GZT9	Egl nine homolog 1 OS=Homo sapiens OX=9606 GN=EGLN1 PE=1 SV=1	0	5.297	4	1	1	1	426	46	8.53	3.43	1	0	60510.66016		NCDN only
Q99865	Spindlin-2A OS=Homo sapiens OX=9606 GN=SPIN2A PE=1 SV=3	0.001	3.446	5	1	1	1	258	29.2	7.23	2.17	1	0	21898.52148		NCDN only
P16260	Graves disease carrier protein OS=Homo sapiens OX=9606 GN=SLC25A16 PE=1 SV=3	0.011	1.547	2	1	1	1	332	36.2	9.85	0	1	0	62588.60156		NCDN only
Q1KMD3	Heterogeneous nuclear ribonucleoprotein U-like protein 2 OS=Homo sapiens OX=9606 GN=HNRNPUL2 PE=1 SV=1	0.005	1.821	1	1	1	1	747	85.1	4.91	0	1	0	27391.5625		NCDN only
Q5K4L6	Solute carrier family 27 member 3 OS=Homo sapiens OX=9606 GN=SLC27A3 PE=1 SV=4	0.002	2.946	1	1	1	1	683	73.5	7.09	2.41	1	0	90506.10156		NCDN only
Q15286	Ras-related protein Rab-35 OS=Homo sapiens OX=9606 GN=RAB35 PE=1 SV=1	0.002	2.56	4	1	1	1	201	23	8.29	0	1	0	32163.75977		NCDN only
P52434	DNA-directed RNA polymerases I, II, and III subunit RPABC3 OS=Homo sapiens OX=9606 GN=POLR2H PE=1 SV=4	0.002	2.921	9	1	1	1	150	17.1	4.68	0	1	0	26199.86523		NCDN only
O94766	Galactosylgalactosylxylosylprotein 3-beta-glucuronosyltransferase 3 OS=Homo sapiens OX=9606 GN=B3GAT3 PE=1 SV=2	0.008	1.655	2	1	1	1	335	37.1	8.27	2.01	1	0	90557.59375		NCDN only
Q9Y2R0	Cytochrome c oxidase assembly factor 3 homolog, mitochondrial OS=Homo sapiens OX=9606 GN=COA3 PE=1 SV=1	0.002	2.366	12	1	1	1	106	11.7	9.6	2.19	1	0	105168.1953		NCDN only
Q9H330	Transmembrane protein 245 OS=Homo sapiens OX=9606 GN=TMEM245 PE=1 SV=3	0	3.597	1	1	1	1	879	97.3	8.91	2.22	1	0	27976.83203		NCDN only
Q9H0B6	Kinesin light chain 2 OS=Homo sapiens OX=9606 GN=KLC2 PE=1 SV=1	0.008	1.586	2	1	1	1	622	68.9	7.15	0	1	0	328034.7188		NCDN only
P56385	ATP synthase subunit e, mitochondrial OS=Homo sapiens OX=9606 GN=ATP5ME PE=1 SV=2	0	3.658	14	1	1	1	69	7.9	9.35	2.37	1	0	1774977		NCDN only
Q8WVQ1	Soluble calcium-activated nucleotidase 1 OS=Homo sapiens OX=9606 GN=CANT1 PE=1 SV=1	0.022	1.429	2	1	1	1	401	44.8	6.09	0	1	0	37030.21875		NCDN only
Q9P1Y6	PHD and RING finger domain-containing protein 1 OS=Homo sapiens OX=9606 GN=PHRF1 PE=1 SV=3	0.002	2.61	0	1	1	1	1649	178.6	8.95	1.91	1	0	35595.38281		NCDN only
P28340	DNA polymerase delta catalytic subunit OS=Homo sapiens OX=9606 GN=POLD1 PE=1 SV=2	0.008	1.633	1	1	1	1	1107	123.6	7.03	0	1	0	33364.9375		NCDN only
Q13485	Mothers against decapentaplegic homolog 4 OS=Homo sapiens OX=9606 GN=SMAD4 PE=1 SV=1	0	4.297	2	1	1	1	552	60.4	6.99	2.46	1	0	56434.27734		NCDN only
Q9NQP4	Prefoldin subunit 4 OS=Homo sapiens OX=9606 GN=PFDN4 PE=1 SV=1	0.022	1.417	4	1	1	1	134	15.3	4.53	0	1	0	58430.84766		NCDN only
Q16527	Cysteine and glycine-rich protein 2 OS=Homo sapiens OX=9606 GN=CSRP2 PE=1 SV=3	0.002	3.07	5	1	1	1	193	20.9	8.62	2.36	1	0	284532.75		NCDN only
Q07065	Cytoskeleton-associated protein 4 OS=Homo sapiens OX=9606 GN=CKAP4 PE=1 SV=2	0	139.85	41	23	82	23	602	66	5.92	211.97	23	0	227946385.4	9125.010254	24980.40
O75190	DnaJ homolog subfamily B member 6 OS=Homo sapiens OX=9606 GN=DNAJB6 PE=1 SV=2	0	5.528	5	2	24	2	326	36.1	9.16	3.59	2	0	7422596.711	2045.191284	3629.29
Q9Y277	Voltage-dependent anion-selective channel protein 3 OS=Homo sapiens OX=9606 GN=VDAC3 PE=1 SV=1	0	12.211	10	3	28	2	283	30.6	8.66	48.15	3	0	8723609.391	2645.671875	3297.31
P57088	Transmembrane protein 33 OS=Homo sapiens OX=9606 GN=TMEM33 PE=1 SV=2	0	15.145	13	3	6	3	247	28	9.7	19.95	3	0	36739005.66	14236.81152	2580.56
P51648	Aldehyde dehydrogenase family 3 member A2 OS=Homo sapiens OX=9606 GN=ALDH3A2 PE=1 SV=1	0	18.077	11	6	54	6	485	54.8	7.88	53.8	6	0	20637431.12	8380.341797	2462.60
Q16891	MICOS complex subunit MIC60 OS=Homo sapiens OX=9606 GN=IMMT PE=1 SV=1	0	94.339	23	20	89	20	758	83.6	6.48	175.04	20	0	25553213.65	10837.11523	2357.94
Q9UBB6	Neurochondrin OS=Homo sapiens OX=9606 GN=NCDN PE=1 SV=1	0	311.221	45	32	453	32	729	78.8	5.48	884.24	32	0	29490515057	12646257.81	2331.96
O95831	Apoptosis-inducing factor 1, mitochondrial OS=Homo sapiens OX=9606 GN=AIFM1 PE=1 SV=1	0	14.002	5	3	19	3	613	66.9	8.95	49.32	3	0	11267821.69	6012.748047	1873.99
P04843	Dolichyl-diphosphooligosaccharide--protein glycosyltransferase subunit 1 OS=Homo sapiens OX=9606 GN=RPN1 PE=1 SV=1	0	17.235	9	6	14	6	607	68.5	6.38	22.28	6	0	8403738.836	4762.869141	1764.43
O95292	Vesicle-associated membrane protein-associated protein B/C OS=Homo sapiens OX=9606 GN=VAPB PE=1 SV=3	0	7.655	12	4	16	4	243	27.2	7.3	19.58	4	0	8276544.117	5917.756104	1398.60
O76013	Keratin, type I cuticular Ha6 OS=Homo sapiens OX=9606 GN=KRT36 PE=1 SV=1	0	24.199	10	6	45	4	467	52.2	4.94	78.36	6	0	65644147.38	48684.07324	1348.37
P61619	Protein transport protein Sec61 subunit alpha isoform 1 OS=Homo sapiens OX=9606 GN=SEC61A1 PE=1 SV=2	0	14.874	8	4	22	4	476	52.2	8.06	14.29	4	0	2525966.516	2080.384033	1214.18
Q96IX5	ATP synthase membrane subunit K, mitochondrial OS=Homo sapiens OX=9606 GN=ATP5MK PE=1 SV=1	0	35.675	47	3	7	3	58	6.5	9.76	31.73	3	0	48554393.44	41949.21094	1157.46
O14880	Microsomal glutathione S-transferase 3 OS=Homo sapiens OX=9606 GN=MGST3 PE=1 SV=1	0.002	2.853	5	1	22	1	152	16.5	9.38	46.51	1	0	394725544.2	363438.7578	1086.09
P78385	Keratin, type II cuticular Hb3 OS=Homo sapiens OX=9606 GN=KRT83 PE=1 SV=2	0	59.32	19	14	179	6	493	54.2	5.64	269.31	14	7	812232936.8	760825.8711	1067.57
Q7Z7H5	Transmembrane emp24 domain-containing protein 4 OS=Homo sapiens OX=9606 GN=TMED4 PE=1 SV=1	0	6.097	7	2	47	1	227	25.9	8.28	55.29	2	0	1220986.016	1490.952271	818.93
P08754	Guanine nucleotide-binding protein G(i) subunit alpha-3 OS=Homo sapiens OX=9606 GN=GNAI3 PE=1 SV=3	0	16.729	12	4	10	3	354	40.5	5.69	20.38	4	0	2267440.867	3103.066406	730.71
Q9NSB4	Keratin, type II cuticular Hb2 OS=Homo sapiens OX=9606 GN=KRT82 PE=1 SV=3	0	22.16	9	5	121	3	513	56.6	6.74	195.86	5	0	268867839.7	374316.0781	718.29
Q8N2K0	Lysophosphatidylserine lipase ABHD12 OS=Homo sapiens OX=9606 GN=ABHD12 PE=1 SV=2	0	7.556	5	2	10	2	398	45.1	8.65	22.06	2	0	10749838.94	17756.13794	605.42
P11586	C-1-tetrahydrofolate synthase, cytoplasmic OS=Homo sapiens OX=9606 GN=MTHFD1 PE=1 SV=4	0	6.865	3	3	7	3	935	101.5	7.3	9.51	3	0	2129073.688	3542.905273	600.94
P50402	Emerin OS=Homo sapiens OX=9606 GN=EMD PE=1 SV=1	0	11.543	9	2	20	2	254	29	5.5	46.31	2	0	20651114.44	41075.09082	502.76
Q9BVK6	Transmembrane emp24 domain-containing protein 9 OS=Homo sapiens OX=9606 GN=TMED9 PE=1 SV=2	0	15.169	10	3	41	2	235	27.3	8.02	44.26	3	1	6352180.563	12757.59863	497.91
P62258	14-3-3 protein epsilon OS=Homo sapiens OX=9606 GN=YWHAE PE=1 SV=1	0	9.504	10	3	20	2	255	29.2	4.74	37.39	3	0	4301104.25	8911.736328	482.63
O96008	Mitochondrial import receptor subunit TOM40 homolog OS=Homo sapiens OX=9606 GN=TOMM40 PE=1 SV=1	0	56.771	17	5	97	5	361	37.9	7.25	202.55	5	0	149828793.6	320810.9521	467.03
O96005	Cleft lip and palate transmembrane protein 1 OS=Homo sapiens OX=9606 GN=CLPTM1 PE=1 SV=1	0	13.599	5	3	15	3	669	76	6.3	26.06	3	0	880884.9609	1933.699097	455.54
O75396	Vesicle-trafficking protein SEC22b OS=Homo sapiens OX=9606 GN=SEC22B PE=1 SV=4	0	10.267	13	3	19	3	215	24.6	6.92	23.28	3	0	2097572.953	4963.517578	422.60
Q9NVI7	ATPase family AAA domain-containing protein 3A OS=Homo sapiens OX=9606 GN=ATAD3A PE=1 SV=2	0	15.685	6	4	27	4	634	71.3	8.98	47.01	4	0	2508512.367	5990.097168	418.78
Q53H12	Acylglycerol kinase, mitochondrial OS=Homo sapiens OX=9606 GN=AGK PE=1 SV=2	0	16.18	8	5	62	5	422	47.1	8.09	108.06	5	0	16301456.88	42885.36157	380.12
Q09028	Histone-binding protein RBBP4 OS=Homo sapiens OX=9606 GN=RBBP4 PE=1 SV=3	0.007	1.691	2	1	3	1	425	47.6	4.89	3.71	1	0	3024751.125	8171.345703	370.17
Q15363	Transmembrane emp24 domain-containing protein 2 OS=Homo sapiens OX=9606 GN=TMED2 PE=1 SV=1	0	10.783	8	2	60	2	201	22.7	5.17	63.76	2	0	8301728.168	22601.4043	367.31
P22695	Cytochrome b-c1 complex subunit 2, mitochondrial OS=Homo sapiens OX=9606 GN=UQCRC2 PE=1 SV=3	0	8.446	7	4	12	4	453	48.4	8.63	18.44	4	0	2171332.035	6213.82373	349.44
Q6NUK1	Calcium-binding mitochondrial carrier protein SCaMC-1 OS=Homo sapiens OX=9606 GN=SLC25A24 PE=1 SV=2	0	6.915	5	3	6	3	477	53.3	6.33	11.86	3	0	391511.8438	1168.512817	335.05
Q53GQ0	Very-long-chain 3-oxoacyl-CoA reductase OS=Homo sapiens OX=9606 GN=HSD17B12 PE=1 SV=2	0	5.652	4	2	10	2	312	34.3	9.32	8.12	2	0	2115776.566	6355.140625	332.92
P63244	Receptor of activated protein C kinase 1 OS=Homo sapiens OX=9606 GN=RACK1 PE=1 SV=3	0	7.852	5	2	8	2	317	35.1	7.69	11.92	2	0	813404.3438	2474.416748	328.73
Q96TA2	ATP-dependent zinc metalloprotease YME1L1 OS=Homo sapiens OX=9606 GN=YME1L1 PE=1 SV=2	0	57.552	16	13	62	13	773	86.4	8.76	116.49	13	0	49999970.86	162708.4309	307.30
Q9NS69	Mitochondrial import receptor subunit TOM22 homolog OS=Homo sapiens OX=9606 GN=TOMM22 PE=1 SV=3	0	11.462	24	2	14	2	142	15.5	4.34	23.63	2	0	3227722.68	11714.50586	275.53
Q02978	Mitochondrial 2-oxoglutarate/malate carrier protein OS=Homo sapiens OX=9606 GN=SLC25A11 PE=1 SV=3	0	45.687	20	6	52	6	314	34	9.91	92.2	6	0	40438179.35	150614.5289	268.49
Q9BSF4	Mitochondrial import inner membrane translocase subunit Tim29 OS=Homo sapiens OX=9606 GN=TIMM29 PE=1 SV=2	0	7.732	8	2	5	2	260	29.2	8.09	12.42	2	0	724955.6406	2762.943848	262.39
Q9Y282	Endoplasmic reticulum-Golgi intermediate compartment protein 3 OS=Homo sapiens OX=9606 GN=ERGIC3 PE=1 SV=1	0.002	2.745	2	1	3	1	383	43.2	6.06	4.74	1	0	1310294.555	5046.97998	259.62
P45880	Voltage-dependent anion-selective channel protein 2 OS=Homo sapiens OX=9606 GN=VDAC2 PE=1 SV=2	0	41.746	26	6	28	6	294	31.5	7.56	71.78	6	0	213025681.4	848412.875	251.09
P27824	Calnexin OS=Homo sapiens OX=9606 GN=CANX PE=1 SV=2	0	6.652	3	2	7	2	592	67.5	4.6	10.9	2	0	2438202.08	10032.56738	243.03
Q5TGZ0	MICOS complex subunit MIC10 OS=Homo sapiens OX=9606 GN=MICOS10 PE=1 SV=1	0	3.773	12	2	6	2	78	8.8	8.5	7.39	2	0	520942.6094	2200.312988	236.76
P20700	Lamin-B1 OS=Homo sapiens OX=9606 GN=LMNB1 PE=1 SV=2	0	30.248	15	9	26	7	586	66.4	5.16	51.42	9	2	3733040.68	16726.09106	223.19
P46020	Phosphorylase b kinase regulatory subunit alpha, skeletal muscle isoform OS=Homo sapiens OX=9606 GN=PHKA1 PE=1 SV=2	0	12.301	3	5	18	5	1223	137.2	6.19	28.07	5	0	3778671.57	17196.0564	219.74
P51809	Vesicle-associated membrane protein 7 OS=Homo sapiens OX=9606 GN=VAMP7 PE=1 SV=3	0	5.342	10	3	19	3	220	24.9	8.6	19.25	3	0	1841350.936	8451.568359	217.87
Q9UDW1	Cytochrome b-c1 complex subunit 9 OS=Homo sapiens OX=9606 GN=UQCR10 PE=1 SV=3	0	4.852	14	1	6	1	63	7.3	9.47	8.73	1	0	2957170.126	14119.90625	209.43
Q15366	Poly(rC)-binding protein 2 OS=Homo sapiens OX=9606 GN=PCBP2 PE=1 SV=1	0	33.174	14	4	15	2	365	38.6	6.79	50.47	4	0	11067682.25	53697.73242	206.11
P43243	Matrin-3 OS=Homo sapiens OX=9606 GN=MATR3 PE=1 SV=2	0	10.981	5	5	11	5	847	94.6	6.25	5.49	5	0	460955.3047	2349.533691	196.19
O76009	Keratin, type I cuticular Ha3-I OS=Homo sapiens OX=9606 GN=KRT33A PE=2 SV=2	0	49.725	30	11	67	2	404	45.9	4.82	120.5	11	7	520427798.3	2756183.531	188.82
P13797	Plastin-3 OS=Homo sapiens OX=9606 GN=PLS3 PE=1 SV=4	0	13.642	4	3	14	3	630	70.8	5.6	19.66	3	0	1085403.84	6255.457031	173.51
Q86SK9	Stearoyl-CoA desaturase 5 OS=Homo sapiens OX=9606 GN=SCD5 PE=1 SV=2	0	16.026	12	3	23	3	330	37.6	9.61	47.31	3	0	1036343.273	5989.04834	173.04
P42677	40S ribosomal protein S27 OS=Homo sapiens OX=9606 GN=RPS27 PE=1 SV=3	0.002	2.634	10	1	3	1	84	9.5	9.45	2.06	1	0	460692.6563	2772.229919	166.18
P62851	40S ribosomal protein S25 OS=Homo sapiens OX=9606 GN=RPS25 PE=1 SV=1	0	4.938	13	2	7	2	125	13.7	10.11	10.69	2	0	1613041	9844.50293	163.85
Q13835	Plakophilin-1 OS=Homo sapiens OX=9606 GN=PKP1 PE=1 SV=2	0	17.063	3	2	5	2	747	82.8	9.13	9.51	2	0	1633090.875	10008.31152	163.17
Q99623	Prohibitin-2 OS=Homo sapiens OX=9606 GN=PHB2 PE=1 SV=2	0	21.245	22	7	62	7	299	33.3	9.83	88.57	7	0	12056678.8	76330.33887	157.95
O75643	U5 small nuclear ribonucleoprotein 200 kDa helicase OS=Homo sapiens OX=9606 GN=SNRNP200 PE=1 SV=2	0	24.899	3	6	17	6	2136	244.4	6.06	39.34	6	0	1889646.344	12478.2959	151.43
P0C7P4	Putative cytochrome b-c1 complex subunit Rieske-like protein 1 OS=Homo sapiens OX=9606 GN=UQCRFS1P1 PE=5 SV=1	0	5.05	6	2	9	2	283	30.8	8.87	17.84	2	0	4356168.23	30950.13672	140.75
Q9Y4W6	AFG3-like protein 2 OS=Homo sapiens OX=9606 GN=AFG3L2 PE=1 SV=2	0	14.718	5	5	11	5	797	88.5	8.66	21.57	5	0	1903780.734	13707.75293	138.88
P62913	60S ribosomal protein L11 OS=Homo sapiens OX=9606 GN=RPL11 PE=1 SV=2	0	17.431	16	3	20	3	178	20.2	9.6	34.97	3	0	45390427.19	329641.7246	137.70
Q9Y266	Nuclear migration protein nudC OS=Homo sapiens OX=9606 GN=NUDC PE=1 SV=1	0	5.1	5	2	6	2	331	38.2	5.38	11.45	2	0	1223793.461	8928.061523	137.07
P36542	ATP synthase subunit gamma, mitochondrial OS=Homo sapiens OX=9606 GN=ATP5F1C PE=1 SV=1	0	20.833	16	5	20	5	298	33	9.22	20.26	5	0	1394298.852	10615.02148	131.35

Q99832	T-complex protein 1 subunit eta OS=Homo sapiens OX=9606 GN=CCT7 PE=1 SV=2	0	5.24	4	2	10	2	543	59.3	7.65	23.85	2	0	3702878.125	28342.5625	130.65
Q15392	Delta(24)-sterol reductase OS=Homo sapiens OX=9606 GN=DHCR24 PE=1 SV=2	0	4.602	3	2	8	2	516	60.1	8.16	18.21	2	0	17608787.58	145896.7969	120.69
P18085	ADP-ribosylation factor 4 OS=Homo sapiens OX=9606 GN=ARF4 PE=1 SV=3	0	6.507	9	2	11	1	180	20.5	7.14	19.76	2	1	11411788.53	95749.17676	119.18
O15397	Importin-8 OS=Homo sapiens OX=9606 GN=IPO8 PE=1 SV=2	0	9.754	2	2	9	2	1037	119.9	5.16	14.37	2	0	2576460.781	22109.61133	116.53
Q4VCS5	Angiomotin OS=Homo sapiens OX=9606 GN=AMOT PE=1 SV=1	0	5.802	2	2	5	2	1084	118	7.64	8.32	2	0	319082.6152	2792.374268	114.27
P24539	ATP synthase F(0) complex subunit B1, mitochondrial OS=Homo sapiens OX=9606 GN=ATP5PB PE=1 SV=2	0	5.623	6	2	7	2	256	28.9	9.36	13.8	2	0	1661487.328	14577.59668	113.98
P28288	ATP-binding cassette sub-family D member 3 OS=Homo sapiens OX=9606 GN=ABCD3 PE=1 SV=1	0	39.011	12	11	161	11	659	75.4	9.36	265.52	11	0	82201195.22	741636.6646	110.84
Q8WWC4	m-AAA protease-interacting protein 1, mitochondrial OS=Homo sapiens OX=9606 GN=MAIP1 PE=1 SV=1	0	5.841	6	2	4	2	291	32.5	9.17	7.55	2	0	1299157.215	12063.19336	107.70
Q86VR2	Reticulophagy regulator 3 OS=Homo sapiens OX=9606 GN=RETREG3 PE=1 SV=1	0	6.058	6	2	15	2	466	51.4	4.97	26.67	2	0	898548.2617	8350.348633	107.61
P46782	40S ribosomal protein S5 OS=Homo sapiens OX=9606 GN=RPS5 PE=1 SV=4	0	17.74	18	4	17	4	204	22.9	9.72	31.96	4	0	31757443.95	310539.6289	102.27
Q9NZ01	Very-long-chain enoyl-CoA reductase OS=Homo sapiens OX=9606 GN=TECR PE=1 SV=1	0	16.552	16	6	17	6	308	36	9.45	24.92	6	0	35832228.4	356438.375	100.53
O14929	Histone acetyltransferase type B catalytic subunit OS=Homo sapiens OX=9606 GN=HAT1 PE=1 SV=1	0	14.786	8	3	8	3	419	49.5	5.69	9.2	3	0	662780.9414	6744.159058	98.27
P37108	Signal recognition particle 14 kDa protein OS=Homo sapiens OX=9606 GN=SRP14 PE=1 SV=2	0	4.38	7	2	6	2	136	14.6	10.04	10.45	2	0	1330840.021	13562.99854	98.12
O14773	Tripeptidyl-peptidase 1 OS=Homo sapiens OX=9606 GN=TPP1 PE=1 SV=2	0.008	1.582	1	1	3	1	563	61.2	6.48	1.65	1	0	493459.4453	5147.580078	95.86
O14828	Secretory carrier-associated membrane protein 3 OS=Homo sapiens OX=9606 GN=SCAMP3 PE=1 SV=3	0	42.789	16	5	14	5	347	38.3	7.64	50.06	5	0	13640500.11	148097.6211	92.10
P27816	Microtubule-associated protein 4 OS=Homo sapiens OX=9606 GN=MAP4 PE=1 SV=3	0	4.206	1	1	3	1	1152	120.9	5.43	6.95	1	0	151888.748	1751.147827	86.74
Q9BXP2	Solute carrier family 12 member 9 OS=Homo sapiens OX=9606 GN=SLC12A9 PE=1 SV=1	0.008	1.61	1	1	2	1	914	96	8.07	1.71	1	0	121165.9258	1443.99231	83.91
Q00325	Phosphate carrier protein, mitochondrial OS=Homo sapiens OX=9606 GN=SLC25A3 PE=1 SV=2	0	39.745	18	8	24	8	362	40.1	9.38	41.44	8	0	68408704.4	827015.0098	82.72
P21796	Voltage-dependent anion-selective channel protein 1 OS=Homo sapiens OX=9606 GN=VDAC1 PE=1 SV=2	0	14.068	14	4	11	3	283	30.8	8.54	22.74	4	0	10147377.28	123461.5869	82.19
Q9Y230	RuvB-like 2 OS=Homo sapiens OX=9606 GN=RUVBL2 PE=1 SV=3	0	5.735	4	2	5	2	463	51.1	5.64	4.46	2	0	525464.0625	7026.638184	74.78
P62829	60S ribosomal protein L23 OS=Homo sapiens OX=9606 GN=RPL23 PE=1 SV=1	0.002	3.211	7	1	4	1	140	14.9	10.51	9.7	1	0	5545740	77273.18359	71.77
P12236	ADP/ATP translocase 3 OS=Homo sapiens OX=9606 GN=SLC25A6 PE=1 SV=4	0	31.846	24	8	325	1	298	32.8	9.74	267.67	8	0	14500812	210637.0156	68.84
P69905	Hemoglobin subunit alpha OS=Homo sapiens OX=9606 GN=HBA1 PE=1 SV=2	0	9.443	17	2	6	2	142	15.2	8.68	15.7	2	0	24067682	358958.0781	67.05
O75964	ATP synthase subunit g, mitochondrial OS=Homo sapiens OX=9606 GN=ATP5MG PE=1 SV=3	0	3.494	11	1	1	1	103	11.4	9.64	1.9	1	0	942414.5625	14077.38574	66.95
Q14134	Tripartite motif-containing protein 29 OS=Homo sapiens OX=9606 GN=TRIM29 PE=1 SV=2	0.001	3.413	2	1	2	1	588	65.8	7.15	3.75	1	0	93376.80469	1497.172485	62.37
Q6P2Q9	Pre-mRNA-processing-splicing factor 8 OS=Homo sapiens OX=9606 GN=PRPF8 PE=1 SV=2	0	75.254	8	21	86	21	2335	273.4	8.84	146.75	21	0	40377046.89	658397.0898	61.33
Q96J01	THO complex subunit 3 OS=Homo sapiens OX=9606 GN=THOC3 PE=1 SV=1	0.005	1.954	2	1	2	1	351	38.7	6.09	4.36	1	0	460157.5938	7925.903809	58.06
P37268	Squalene synthase OS=Homo sapiens OX=9606 GN=FDFT1 PE=1 SV=1	0	14.118	9	4	16	4	417	48.1	6.54	26.28	4	0	2364631.805	41522.44922	56.95
Q13257	Mitotic spindle assembly checkpoint protein MAD2A OS=Homo sapiens OX=9606 GN=MAD2L1 PE=1 SV=1	0.002	2.918	4	1	5	1	205	23.5	5.08	9.01	1	0	881605.6406	15482.6123	56.94
P67775	Serine/threonine-protein phosphatase 2A catalytic subunit alpha isoform OS=Homo sapiens OX=9606 GN=PPP2CA PE=1 SV=1	0	6.033	5	2	9	1	309	35.6	5.54	17.08	2	0	136012.125	2519.291992	53.99
P61106	Ras-related protein Rab-14 OS=Homo sapiens OX=9606 GN=RAB14 PE=1 SV=4	0	9.056	15	4	29	4	215	23.9	6.21	55.17	4	0	1058282.539	19667.00684	53.81
Q9UBX3	Mitochondrial dicarboxylate carrier OS=Homo sapiens OX=9606 GN=SLC25A10 PE=1 SV=2	0	7.887	9	3	4	3	287	31.3	9.54	3.37	3	0	5889502.844	110356.7813	53.37
P06748	Nucleophosmin OS=Homo sapiens OX=9606 GN=NPM1 PE=1 SV=2	0	16.4	12	5	36	5	294	32.6	4.78	59.23	5	0	24418151.67	465488.6753	52.46
P53396	ATP-citrate synthase OS=Homo sapiens OX=9606 GN=ACLY PE=1 SV=3	0	10.31	3	4	7	4	1101	120.8	7.33	10.65	4	0	539733.7734	10293.61963	52.43
P46779	60S ribosomal protein L28 OS=Homo sapiens OX=9606 GN=RPL28 PE=1 SV=3	0	12.487	21	3	11	3	137	15.7	12.02	23.56	3	0	3856474.969	77491.24219	49.77
Q9H3K6	BolA-like protein 2 OS=Homo sapiens OX=9606 GN=BOLA2 PE=1 SV=1	0.002	2.212	8	1	3	1	86	10.1	6.52	5.52	1	0	1980010.5	41416.91797	47.81
P84085	ADP-ribosylation factor 5 OS=Homo sapiens OX=9606 GN=ARF5 PE=1 SV=2	0	6.549	9	2	14	1	180	20.5	6.79	28.39	2	0	2963154.609	62606.76953	47.33
Q9NX24	H/ACA ribonucleoprotein complex subunit 2 OS=Homo sapiens OX=9606 GN=NHP2 PE=1 SV=1	0	4.586	7	1	4	1	153	17.2	8.22	8.7	1	0	271215.7109	5797.387207	46.78
Q9BT22	Chitobiosyldiphosphodolichol beta-mannosyltransferase OS=Homo sapiens OX=9606 GN=ALG1 PE=1 SV=2	0	4.568	3	2	11	2	464	52.5	7.23	24.55	2	0	3601754.953	81380.25	44.26
P98175	RNA-binding protein 10 OS=Homo sapiens OX=9606 GN=RBM10 PE=1 SV=3	0	7.317	3	3	13	3	930	103.5	5.97	11.49	3	0	2197576.938	50421.92188	43.58
Q15758	Neutral amino acid transporter B(0) OS=Homo sapiens OX=9606 GN=SLC1A5 PE=1 SV=2	0	7.051	2	1	7	1	541	56.6	5.48	12.73	1	0	226413.8281	5211.504883	43.44
Q9Y6Y0	Influenza virus NS1A-binding protein OS=Homo sapiens OX=9606 GN=IVNS1ABP PE=1 SV=3	0	18.598	6	4	25	4	642	71.7	5.53	10.94	4	0	268876.8809	6275.728516	42.84
P25705	ATP synthase subunit alpha, mitochondrial OS=Homo sapiens OX=9606 GN=ATP5F1A PE=1 SV=1	0	41.674	17	9	70	9	553	59.7	9.13	101.11	9	0	15860404.8	374916.2368	42.30
Q92945	Far upstream element-binding protein 2 OS=Homo sapiens OX=9606 GN=KHSRP PE=1 SV=4	0	34.922	12	5	18	4	711	73.1	7.3	58.84	5	1	4202260.719	99559.50586	42.21
Q9NQE9	Histidine triad nucleotide-binding protein 3 OS=Homo sapiens OX=9606 GN=HINT3 PE=1 SV=1	0.006	1.8	4	1	2	1	182	20.3	6.6	4.23	1	0	259563.2266	6218.539551	41.74
O14735	CDP-diacylglycerol--inositol 3-phosphatidyltransferase OS=Homo sapiens OX=9606 GN=CDIPT PE=1 SV=1	0	14.853	10	2	17	2	213	23.5	8.03	31.98	2	0	15377004.27	381149.4375	40.34
O00483	Cytochrome c oxidase subunit NDUFA4 OS=Homo sapiens OX=9606 GN=NDUFA4 PE=1 SV=1	0	4.306	20	2	9	2	81	9.4	9.38	10.41	2	0	8815705.5	228671.2813	38.55
P24534	Elongation factor 1-beta OS=Homo sapiens OX=9606 GN=EEF1B2 PE=1 SV=3	0.007	1.726	3	1	3	1	225	24.7	4.67	6.17	1	0	1044315.25	27174.79492	38.43
Q8ND83	SLAIN motif-containing protein 1 OS=Homo sapiens OX=9606 GN=SLAIN1 PE=1 SV=3	0	6.521	4	2	4	2	568	60.6	7.64	5.46	2	0	90016.34375	2356.870361	38.19
P29144	Tripeptidyl-peptidase 2 OS=Homo sapiens OX=9606 GN=TPP2 PE=1 SV=4	0.002	2.351	1	1	2	1	1249	138.3	6.32	2	1	0	59798.08594	1569.057983	38.11
P07900	Heat shock protein HSP 90-alpha OS=Homo sapiens OX=9606 GN=HSP90AA1 PE=1 SV=5	0	57.949	11	8	80	3	732	84.6	5.02	107.77	8	0	2057480.671	54089.17383	38.04
P38646	Stress-70 protein, mitochondrial OS=Homo sapiens OX=9606 GN=HSPA9 PE=1 SV=2	0	23.912	8	5	27	5	679	73.6	6.16	50.08	5	0	8977789.375	236486.9961	37.96
Q4G148	Glucoside xylosyltransferase 1 OS=Homo sapiens OX=9606 GN=GXYLT1 PE=1 SV=2	0.002	2.119	2	1	1	1	440	50.5	8.65	1.99	1	0	86292.72656	2310.549316	37.35
Q9P0L0	Vesicle-associated membrane protein-associated protein A OS=Homo sapiens OX=9606 GN=VAPA PE=1 SV=3	0	17.664	14	4	21	4	249	27.9	8.62	26.13	4	0	5047718.031	135541.7976	37.24
Q02487	Desmocollin-2 OS=Homo sapiens OX=9606 GN=DSC2 PE=1 SV=1	0	4.666	2	2	4	2	901	99.9	5.34	8.07	2	0	1013942.297	27621.51758	36.71
P62280	40S ribosomal protein S11 OS=Homo sapiens OX=9606 GN=RPS11 PE=1 SV=3	0	4.291	10	2	15	2	158	18.4	10.3	21.48	2	0	2854064.078	79291.89453	35.99
P11413	Glucose-6-phosphate 1-dehydrogenase OS=Homo sapiens OX=9606 GN=G6PD PE=1 SV=4	0	6.206	4	2	4	2	515	59.2	6.84	5.68	2	0	566263.4531	15878.14844	35.66
P61224	Ras-related protein Rap-1b OS=Homo sapiens OX=9606 GN=RAP1B PE=1 SV=1	0	11.303	16	3	20	3	184	20.8	5.78	31.05	3	0	1806171.219	50938.99609	35.46
Q8WWY3	U4/U6 small nuclear ribonucleoprotein Prp31 OS=Homo sapiens OX=9606 GN=PRPF31 PE=1 SV=2	0	26.032	10	5	23	5	499	55.4	5.78	38.66	5	0	2662880.699	75538.67358	35.25
P06733	Alpha-enolase OS=Homo sapiens OX=9606 GN=ENO1 PE=1 SV=2	0	9.346	7	4	9	4	434	47.1	7.39	10.02	4	0	1401308.121	39812.73145	35.20
P48643	T-complex protein 1 subunit epsilon OS=Homo sapiens OX=9606 GN=CCT5 PE=1 SV=1	0	6.059	3	2	4	2	541	59.6	5.66	8.56	2	0	9735586.18	280292.4375	34.73
Q9H7Z7	Prostaglandin E synthase 2 OS=Homo sapiens OX=9606 GN=PTGES2 PE=1 SV=1	0	4.441	5	2	4	2	377	41.9	9.16	3.91	2	0	113484.7617	3275.143311	34.65
Q53H96	Pyrroline-5-carboxylate reductase 3 OS=Homo sapiens OX=9606 GN=PYCR3 PE=1 SV=3	0.002	2.329	3	1	2	1	274	28.6	7.72	4.01	1	0	287954.6211	8327.948242	34.58
O00232	26S proteasome non-ATPase regulatory subunit 12 OS=Homo sapiens OX=9606 GN=PSMD12 PE=1 SV=3	0	7.009	2	1	6	1	456	52.9	7.65	8.18	1	0	120655.0137	3608.744141	33.43
Q9BTV4	Transmembrane protein 43 OS=Homo sapiens OX=9606 GN=TMEM43 PE=1 SV=1	0	67.747	21	7	55	7	400	44.8	8.13	101.5	7	0	58377098.77	1801150.047	32.41
P04406	Glyceraldehyde-3-phosphate dehydrogenase OS=Homo sapiens OX=9606 GN=GAPDH PE=1 SV=3	0	57.564	30	11	31	11	335	36	8.46	40.33	11	0	40940721.3	1265179.141	32.36
Q9H2H9	Sodium-coupled neutral amino acid transporter 1 OS=Homo sapiens OX=9606 GN=SLC38A1 PE=1 SV=1	0.002	2.063	1	1	3	1	487	54	7.02	0	1	0	296466.0391	9202.03418	32.22
P02545	Prelamin-A/C OS=Homo sapiens OX=9606 GN=LMNA PE=1 SV=1	0	18.125	8	6	7	5	664	74.1	7.02	10.26	6	0	579522.7266	18465.62354	31.38
P78527	DNA-dependent protein kinase catalytic subunit OS=Homo sapiens OX=9606 GN=PRKDC PE=1 SV=3	0	22.903	2	8	13	8	4128	468.8	7.12	17.12	8	0	1246530.08	39923.02246	31.22
P09234	U1 small nuclear ribonucleoprotein C OS=Homo sapiens OX=9606 GN=SNRPC PE=1 SV=1	0	3.55	6	1	3	1	159	17.4	9.67	7.61	1	0	177384.3438	5752.225098	30.84
P16104	Histone H2AX OS=Homo sapiens OX=9606 GN=H2AX PE=1 SV=2	0	9.618	17	3	24	1	143	15.1	10.74	31.29	3	0	733455.4375	24281.49414	30.21
P14635	G2/mitotic-specific cyclin-B1 OS=Homo sapiens OX=9606 GN=CCNB1 PE=1 SV=1	0.002	2.083	2	1	2	1	433	48.3	7.47	1.76	1	0	361836.9375	12031.81641	30.07
Q14203	Dynactin subunit 1 OS=Homo sapiens OX=9606 GN=DCTN1 PE=1 SV=3	0.002	2.054	1	1	2	1	1278	141.6	5.81	0	1	0	127771.1875	4254.470703	30.03
P62910	60S ribosomal protein L32 OS=Homo sapiens OX=9606 GN=RPL32 PE=1 SV=2	0.002	2.158	5	1	12	1	135	15.9	11.33	21.55	1	0	597582.3906	20208.38672	29.57
P04439	HLA class I histocompatibility antigen, A alpha chain OS=Homo sapiens OX=9606 GN=HLA-A PE=1 SV=2	0.001	3.468	2	1	1	1	365	40.8	6	2.65	1	0	169712.8438	5742.751465	29.55
P11142	Heat shock cognate 71 kDa protein OS=Homo sapiens OX=9606 GN=HSPA8 PE=1 SV=1	0	87.627	28	16	149	10	646	70.9	5.52	186.68	16	0	35070954.82	1212951.861	28.91
P62857	40S ribosomal protein S28 OS=Homo sapiens OX=9606 GN=RPS28 PE=1 SV=1	0	9.187	29	2	6	2	69	7.8	10.7	11.43	2	0	3272958.688	113724.9531	28.78
P39019	40S ribosomal protein S19 OS=Homo sapiens OX=9606 GN=RPS19 PE=1 SV=2	0	9.441	20	3	9	3	145	16.1	10.32	19.91	3	0	1513407.922	52902.84375	28.61
Q5JPH6	Probable glutamate--tRNA ligase, mitochondrial OS=Homo sapiens OX=9606 GN=EARS2 PE=1 SV=2	0	4.161	3	2	6	2	523	58.7	8.76	5.06	2	0	4004325.441	144284.5781	27.75
P49755	Transmembrane emp24 domain-containing protein 10 OS=Homo sapiens OX=9606 GN=TMED10 PE=1 SV=2	0	4.894	8	2	3	2	219	25	7.44	6.37	2	0	2304437.688	83049.63281	27.75
Q10472	Polypeptide N-acetylgalactosaminyltransferase 1 OS=Homo sapiens OX=9606 GN=GALNT1 PE=1 SV=1	0.002	3.104	2	1	3	1	559	64.2	7.72	6.49	1	0	1374615.688	49617.17969	27.70
P41091	Eukaryotic translation initiation factor 2 subunit 3 OS=Homo sapiens OX=9606 GN=EIF2S3 PE=1 SV=3	0.007	1.753	1	1	1	1	472	51.1	8.4	0	1	0	48549.28125	1759.088989	27.60
Q13263	Transcription intermediary factor 1-beta OS=Homo sapiens OX=9606 GN=TRIM28 PE=1 SV=5	0	10.012	3	3	10	3	835	88.5	5.77	13.92	3	0	851329.0977	31267.89063	27.23
Q12789	General transcription factor 3C polypeptide 1 OS=Homo sapiens OX=9606 GN=GTF3C1 PE=1 SV=4	0.005	1.87	0	1	2	1	2109	238.7	7.3	0	1	0	48280.89063	1811.439087	26.65
P68104	Elongation factor 1-alpha 1 OS=Homo sapiens OX=9606 GN=EEF1A1 PE=1 SV=1	0	71.108	23	10	40	5	462	50.1	9.01	104.45	10	5	196124414.8	7424022.858	26.42
Q01813	ATP-dependent 6-phosphofructokinase, platelet type OS=Homo sapiens OX=9606 GN=PFKP PE=1 SV=2	0.015	1.483	1	1	2	1	784	85.5	7.55	1.62	1	0	111339.5039	4316.330566	25.79
Q9UQ80	Proliferation-associated protein 2G4 OS=Homo sapiens OX=9606 GN=PA2G4 PE=1 SV=3	0	5.466	4	2	3	2	394	43.8	6.55	5.52	2	0	103991.5781	4038.566406	25.75
Q15029	116 kDa U5 small nuclear ribonucleoprotein component OS=Homo sapiens OX=9606 GN=EFTUD2 PE=1 SV=1	0	54.124	15	15	27	14	972	109.4	5	58.78	15	1	85610405.03	3354952.301	25.52
P62249	40S ribosomal protein S16 OS=Homo sapiens OX=9606 GN=RPS16 PE=1 SV=2	0	16.861	25	5	24	5	146	16.4	10.21	29.9	5	0	10694387.75	422625.9744	25.30
Q9BS26	Endoplasmic reticulum resident protein 44 OS=Homo sapiens OX=9606 GN=ERP44 PE=1 SV=1	0.008	1.667	2	1	2	1	406	46.9	5.26	1.75	1	0	110810.0547	4431.649414	25.00
O95352	Ubiquitin-like modifier-activating enzyme ATG7 OS=Homo sapiens OX=9606 GN=ATG7 PE=1 SV=1	0.006	1.78	2	1	1	1	703	77.9	6.24	1.89	1	0	87947.73438	3521.761963	24.97
P62195	26S proteasome regulatory subunit 8 OS=Homo sapiens OX=9606 GN=PSMC5 PE=1 SV=1	0	10.582	5	2	9	2	406	45.6	7.55	20.8	2	0	461657.5625	18764.01709	24.60
P62714	Serine/threonine-protein phosphatase 2A catalytic subunit beta isoform OS=Homo sapiens OX=9606 GN=PPP2CB PE=1 SV=1	0	6.715	5	2	8	1	309	35.6	5.43	14.74	2	1	140257.1406	5801.619141	24.18
O60814	Histone H2B type 1-K OS=Homo sapiens OX=9606 GN=H2BC12 PE=1 SV=3	0	23.191	29	4	32	4	126	13.9	10.32	76.57	4	0	193080244.6	8222035.383	23.48
P0DN79	Cystathionine beta-synthase-like protein OS=Homo sapiens OX=9606 GN=CBSL PE=1 SV=1	0	9.407	5	3	20	3	551	60.5	6.65	44.12	3	0	1672266.662	72264.48291	23.14
Q7Z739	YTH domain-containing family protein 3 OS=Homo sapiens OX=9606 GN=YTHDF3 PE=1 SV=1	0.002	3.093	2	1	3	1	585	63.8	9.04	6.23	1	0	726456.0625	31997.38086	22.70
P49419	Alpha-aminoadipic semialdehyde dehydrogenase OS=Homo sapiens OX=9606 GN=ALDH7A1 PE=1 SV=5	0.005	1.861	2	1	2	1	539	58.5	7.99	1.64	1	0	64406.14258	2945.163818	21.87
Q9Y262	Eukaryotic translation initiation factor 3 subunit L OS=Homo sapiens OX=9606 GN=EIF3L PE=1 SV=1	0	8.345	5	3	3	3	564	66.7	6.34	6.06	3	0	92752.02734	4248.050781	21.83
O15400	Syntaxin-7 OS=Homo sapiens OX=9606 GN=STX7 PE=1 SV=4	0	10.836	17	4	4	4	261	29.8	5.55	8.04	4	0	189444.1699	8839.189453	21.43
P31947	14-3-3 protein sigma OS=Homo sapiens OX=9606 GN=SFN PE=1 SV=1	0	10.196	13	4	27	2	248	27.8	4.74	42.67	4	2	27926283.71	1303839.517	21.42
Q5VV42	Threonylcarbamoyladenosine tRNA methylthiotransferase OS=Homo sapiens OX=9606 GN=CDKAL1 PE=1 SV=1	0	28.41	10	6	13	6	579	65.1	7.46	20.99	6	0	2279236.109	106718.3633	21.36
Q9Y320	Thioredoxin-related transmembrane protein 2 OS=Homo sapiens OX=9606 GN=TMX2 PE=1 SV=1	0.002	2.333	2	1	7	1	296	34	8.69	8.6	1	0	520637.6875	24531.22754	21.22
Q8WVX9	Fatty acyl-CoA reductase 1 OS=Homo sapiens OX=9606 GN=FAR1 PE=1 SV=1	0	5.156	3	2	8	2	515	59.3	9.17	15.06	2	0	2219619.995	104761.0938	21.19
P49915	GMP synthase [glutamine-hydrolyzing] OS=Homo sapiens OX=9606 GN=GMPS PE=1 SV=1	0.002	3.157	1	1	2	1	693	76.7	6.87	3.83	1	0	54059.38281	2578.712646	20.96
P27708	CAD protein OS=Homo sapiens OX=9606 GN=CAD PE=1 SV=3	0	4.849	1	1	5	1	2225	242.8	6.46	12.57	1	0	113252.8438	5416.987793	20.91
P30050	60S ribosomal protein L12 OS=Homo sapiens OX=9606 GN=RPL12 PE=1 SV=1	0	3.515	5	2	3	2	165	17.8	9.42	6.47	2	0	473893.5781	22905.5249	20.69
O15294	UDP-N-acetylglucosamine--peptide N-acetylglucosaminyltransferase 110 kDa subunit OS=Homo sapiens OX=9606 GN=OGT PE=1 SV=3	0	9.741	2	2	8	2	1046	116.9	6.7	17.21	2	0	2942279.461	143313.7258	20.53
P52701	DNA mismatch repair protein Msh6 OS=Homo sapiens OX=9606 GN=MSH6 PE=1 SV=2	0	3.929	1	1	2	1	1360	152.7	6.9	4.1	1	0	55152.85547	2700.410156	20.42
Q14525	Keratin, type I cuticular Ha3-II OS=Homo sapiens OX=9606 GN=KRT33B PE=1 SV=3	0	47.204	23	9	63	1	404	46.2	4.84	123.36	9	0	9599851	472858.0625	20.30
P35080	Profilin-2 OS=Homo sapiens OX=9606 GN=PFN2 PE=1 SV=3	0	9.963	10	1	1	1	140	15	6.99	4.16	1	0	1422844.75	70225.64063	20.26
P55795	Heterogeneous nuclear ribonucleoprotein H2 OS=Homo sapiens OX=9606 GN=HNRNPH2 PE=1 SV=1	0	26.589	12	4	10	3	449	49.2	6.3	20.79	4	0	527420.1953	26131.20068	20.18
P38117	Electron transfer flavoprotein subunit beta OS=Homo sapiens OX=9606 GN=ETFB PE=1 SV=3	0.005	1.943	4	1	1	1	255	27.8	8.1	1.92	1	0	56324.01563	2794.313232	20.16
P22087	rRNA 2'-O-methyltransferase fibrillarin OS=Homo sapiens OX=9606 GN=FBL PE=1 SV=2	0.002	3.072	3	1	3	1	321	33.8	10.18	5.14	1	0	645838.1563	32706.78711	19.75
P54727	UV excision repair protein RAD23 homolog B OS=Homo sapiens OX=9606 GN=RAD23B PE=1 SV=1	0.002	2.145	2	1	1	1	409	43.1	4.84	0	1	0	34895.46875	1771.02771	19.70
P30519	Heme oxygenase 2 OS=Homo sapiens OX=9606 GN=HMOX2 PE=1 SV=2	0.002	2.936	3	1	2	1	316	36	5.41	4.44	1	0	366511.2734	18676.89258	19.62
P21912	Succinate dehydrogenase [ubiquinone] iron-sulfur subunit, mitochondrial OS=Homo sapiens OX=9606 GN=SDHB PE=1 SV=3	0	5.985	7	3	6	3	280	31.6	8.76	5.81	3	0	1579224.844	81068.35938	19.48
P05023	Sodium/potassium-transporting ATPase subunit alpha-1 OS=Homo sapiens OX=9606 GN=ATP1A1 PE=1 SV=1	0	49.461	13	13	25	13	1023	112.8	5.49	47.61	13	0	16261363.99	835956.1222	19.45
Q9NVH1	DnaJ homolog subfamily C member 11 OS=Homo sapiens OX=9606 GN=DNAJC11 PE=1 SV=2	0	9.547	5	3	10	3	559	63.2	8.4	13.97	3	0	3074726.859	162701.3438	18.90
P62888	60S ribosomal protein L30 OS=Homo sapiens OX=9606 GN=RPL30 PE=1 SV=2	0	3.784	15	2	4	2	115	12.8	9.63	3.77	2	0	362505.8047	19241.34277	18.84
Q3SY84	Keratin, type II cytoskeletal 71 OS=Homo sapiens OX=9606 GN=KRT71 PE=1 SV=3	0	15.87	8	6	11	4	523	57.3	6.61	20.88	6	0	226889.0684	12068.97778	18.80
Q15365	Poly(rC)-binding protein 1 OS=Homo sapiens OX=9606 GN=PCBP1 PE=1 SV=2	0	35.995	17	5	19	3	356	37.5	7.09	55.1	5	3	10801648	583124.4219	18.52
P62070	Ras-related protein R-Ras2 OS=Homo sapiens OX=9606 GN=RRAS2 PE=1 SV=1	0	9.924	10	2	4	2	204	23.4	6.01	5.54	2	0	331719.7676	17950.47461	18.48
Q7KZF4	Staphylococcal nuclease domain-containing protein 1 OS=Homo sapiens OX=9606 GN=SND1 PE=1 SV=1	0	7.96	3	2	3	2	910	101.9	7.17	6.95	2	0	170398.1094	9352.138428	18.22
Q13445	Transmembrane emp24 domain-containing protein 1 OS=Homo sapiens OX=9606 GN=TMED1 PE=1 SV=1	0	6.557	7	2	11	2	227	25.2	4.48	13.94	2	0	5000092.047	285539.0313	17.51
Q06830	Peroxiredoxin-1 OS=Homo sapiens OX=9606 GN=PRDX1 PE=1 SV=1	0	46.325	45	9	39	8	199	22.1	8.13	92.65	9	1	238777385.4	13667098.71	17.47
P49411	Elongation factor Tu, mitochondrial OS=Homo sapiens OX=9606 GN=TUFM PE=1 SV=2	0	16.405	11	5	6	5	452	49.5	7.61	10.94	5	0	6610336.5	383717.1504	17.23

Q9BPX3	Condensin complex subunit 3 OS=Homo sapiens OX=9606 GN=NCAPG PE=1 SV=1	0.002	2.111	1	1	1	1	1015	114.3	5.59	1.62	1	0	535219.375	31627.58594	16.92
Q96QR8	Transcriptional activator protein Pur-beta OS=Homo sapiens OX=9606 GN=PURB PE=1 SV=3	0.007	1.745	3	1	1	1	312	33.2	5.43	0	1	0	587683.25	35395.48438	16.60
P40926	Malate dehydrogenase, mitochondrial OS=Homo sapiens OX=9606 GN=MDH2 PE=1 SV=3	0	5.227	5	2	3	2	338	35.5	8.68	6.17	2	0	580183.5078	34962.13867	16.59
P23396	40S ribosomal protein S3 OS=Homo sapiens OX=9606 GN=RPS3 PE=1 SV=2	0	28.266	30	8	32	8	243	26.7	9.66	65.41	8	0	68160947.38	4108488.769	16.59
P07437	Tubulin beta chain OS=Homo sapiens OX=9606 GN=TUBB PE=1 SV=2	0	114.136	39	14	83	2	444	49.6	4.89	179.99	14	14	184067486.5	11252686.24	16.36
P16401	Histone H1.5 OS=Homo sapiens OX=9606 GN=H1-5 PE=1 SV=3	0.002	3.134	4	1	6	1	226	22.6	10.92	13.74	1	0	2610497.406	162122.0195	16.10
P0C0S8	Histone H2A type 1 OS=Homo sapiens OX=9606 GN=H2AC11 PE=1 SV=2	0	24.359	35	5	29	3	130	14.1	10.9	48.86	5	2	131830803.9	8191953.711	16.09
Q70IA6	MOB kinase activator 2 OS=Homo sapiens OX=9606 GN=MOB2 PE=1 SV=1	0	7.821	11	3	4	3	237	26.9	6.79	6.1	3	0	7055917.879	440247.3516	16.03
P08559	Pyruvate dehydrogenase E1 component subunit alpha, somatic form, mitochondrial OS=Homo sapiens OX=9606 GN=PDHA1 PE=1 SV=3	0	5.242	5	2	2	2	390	43.3	8.06	1.77	2	0	7500137.5	469877.5625	15.96
Q9H7B2	Ribosome production factor 2 homolog OS=Homo sapiens OX=9606 GN=RPF2 PE=1 SV=2	0.002	3.075	3	1	3	1	306	35.6	9.99	5.79	1	0	85936.69531	5465.825684	15.72
P50552	Vasodilator-stimulated phosphoprotein OS=Homo sapiens OX=9606 GN=VASP PE=1 SV=3	0.002	3.084	2	1	3	1	380	39.8	8.94	3.89	1	0	300012.9453	19237.23242	15.60
Q93100	Phosphorylase b kinase regulatory subunit beta OS=Homo sapiens OX=9606 GN=PHKB PE=1 SV=3	0	7.333	2	3	5	3	1093	124.8	6.95	8.48	3	0	760372.0625	49721.27344	15.29
Q8N8A6	ATP-dependent RNA helicase DDX51 OS=Homo sapiens OX=9606 GN=DDX51 PE=1 SV=3	0.007	1.757	1	1	1	1	666	72.4	8.16	0	1	0	33187.5	2178.744873	15.23
P36543	V-type proton ATPase subunit E 1 OS=Homo sapiens OX=9606 GN=ATP6V1E1 PE=1 SV=1	0	4.494	8	2	2	2	226	26.1	8	1.97	2	0	68810.00586	4620.866211	14.89
Q9H583	HEAT repeat-containing protein 1 OS=Homo sapiens OX=9606 GN=HEATR1 PE=1 SV=3	0	5.208	0	1	2	1	2144	242.2	6.54	4.57	1	0	64878.69531	4385.362793	14.79
P17844	Probable ATP-dependent RNA helicase DDX5 OS=Homo sapiens OX=9606 GN=DDX5 PE=1 SV=1	0	16.741	8	5	25	3	614	69.1	8.92	44.05	5	2	5291883.064	359498.7686	14.72
P07355	Annexin A2 OS=Homo sapiens OX=9606 GN=ANXA2 PE=1 SV=2	0	16.939	11	4	10	4	339	38.6	7.75	26	4	0	7728221.375	525061.1875	14.72
O43795	Unconventional myosin-Ib OS=Homo sapiens OX=9606 GN=MYO1B PE=1 SV=3	0	5.156	2	3	6	3	1136	131.9	9.38	3.42	3	0	79543.98242	5425.000977	14.66
O43175	D-3-phosphoglycerate dehydrogenase OS=Homo sapiens OX=9606 GN=PHGDH PE=1 SV=4	0	39.418	15	8	26	8	533	56.6	6.71	44.9	8	0	17492597.47	1207207.315	14.49
P12882	Myosin-1 OS=Homo sapiens OX=9606 GN=MYH1 PE=1 SV=3	0	10.271	1	3	7	1	1939	223	5.74	10.42	3	0	1645944.672	117700.0547	13.98
O43414	ERI1 exoribonuclease 3 OS=Homo sapiens OX=9606 GN=ERI3 PE=1 SV=2	0.004	1.997	4	1	2	1	337	37.2	8.07	1.65	1	0	18983.22852	1368.335327	13.87
P21333	Filamin-A OS=Homo sapiens OX=9606 GN=FLNA PE=1 SV=4	0	20.656	3	8	21	8	2647	280.6	6.06	26.26	8	0	2853865.092	210386.0674	13.56
Q92604	Acyl-CoA:lysophosphatidylglycerol acyltransferase 1 OS=Homo sapiens OX=9606 GN=LPGAT1 PE=1 SV=1	0.004	1.99	2	1	1	1	370	43.1	8.92	1.72	1	0	31335.29492	2392.705811	13.10
P23246	Splicing factor, proline- and glutamine-rich OS=Homo sapiens OX=9606 GN=SFPQ PE=1 SV=2	0	25.205	8	6	23	6	707	76.1	9.44	35.39	6	0	5356549.523	409760.3447	13.07
Q99436	Proteasome subunit beta type-7 OS=Homo sapiens OX=9606 GN=PSMB7 PE=1 SV=1	0.002	3.172	3	1	2	1	277	29.9	7.68	4.2	1	0	80559.34766	6163.063477	13.07
P53992	Protein transport protein Sec24C OS=Homo sapiens OX=9606 GN=SEC24C PE=1 SV=3	0.002	2.328	1	1	1	1	1094	118.2	7.06	1.85	1	0	20556.85156	1575.376099	13.05
Q7Z2W4	Zinc finger CCCH-type antiviral protein 1 OS=Homo sapiens OX=9606 GN=ZC3HAV1 PE=1 SV=3	0	3.49	1	1	10	1	902	101.4	8.4	17.07	1	0	417323.7969	32467.14258	12.85
P14618	Pyruvate kinase PKM OS=Homo sapiens OX=9606 GN=PKM PE=1 SV=4	0	11.902	6	5	27	5	531	57.9	7.84	37.33	5	0	3172751.566	248986.8027	12.74
Q9Y2W1	Thyroid hormone receptor-associated protein 3 OS=Homo sapiens OX=9606 GN=THRAP3 PE=1 SV=2	0	28.883	8	8	32	8	955	108.6	10.15	61.95	8	0	8087175.445	635200.4917	12.73
O96011	Peroxisomal membrane protein 11B OS=Homo sapiens OX=9606 GN=PEX11B PE=1 SV=1	0	25.298	20	3	7	3	259	28.4	9.85	23.62	3	0	5147404.469	409124.0059	12.58
P17812	CTP synthase 1 OS=Homo sapiens OX=9606 GN=CTPS1 PE=1 SV=2	0.002	2.586	1	1	10	1	591	66.6	6.46	14.25	1	0	1085354.18	87366.77344	12.42
P08238	Heat shock protein HSP 90-beta OS=Homo sapiens OX=9606 GN=HSP90AB1 PE=1 SV=4	0	83.199	17	12	105	6	724	83.2	5.03	183.83	12	7	120515940.8	9722086.355	12.40
P63104	14-3-3 protein zeta/delta OS=Homo sapiens OX=9606 GN=YWHAZ PE=1 SV=1	0	12.053	12	3	21	2	245	27.7	4.79	38.61	3	0	2935979.234	241125.668	12.18
P62805	Histone H4 OS=Homo sapiens OX=9606 GN=H4C1 PE=1 SV=2	0	28.452	39	4	15	4	103	11.4	11.36	36.34	4	0	73821786.13	6101351.018	12.10
O43819	Protein SCO2 homolog, mitochondrial OS=Homo sapiens OX=9606 GN=SCO2 PE=1 SV=3	0	5.309	7	2	4	2	266	29.8	8.85	4.83	2	0	812649.1563	67965.17969	11.96
Q15233	Non-POU domain-containing octamer-binding protein OS=Homo sapiens OX=9606 GN=NONO PE=1 SV=4	0	5.901	6	3	9	3	471	54.2	8.95	9.19	3	0	477510.5977	40379.86182	11.83
Q92841	Probable ATP-dependent RNA helicase DDX17 OS=Homo sapiens OX=9606 GN=DDX17 PE=1 SV=2	0	21.702	6	4	7	2	729	80.2	8.27	20.1	4	0	1722874.008	146669.9219	11.75
P50991	T-complex protein 1 subunit delta OS=Homo sapiens OX=9606 GN=CCT4 PE=1 SV=4	0	14.745	9	6	38	6	539	57.9	7.83	87.57	6	0	7484187.342	639645.7695	11.70
P61247	40S ribosomal protein S3a OS=Homo sapiens OX=9606 GN=RPS3A PE=1 SV=2	0	16.387	22	6	23	6	264	29.9	9.73	24.2	6	0	4109307.684	354827.7246	11.58
P42025	Beta-centractin OS=Homo sapiens OX=9606 GN=ACTR1B PE=1 SV=1	0.011	1.531	2	1	1	1	376	42.3	6.4	0	1	0	42683.33594	3929.604004	10.86
Q8TCT9	Minor histocompatibility antigen H13 OS=Homo sapiens OX=9606 GN=HM13 PE=1 SV=1	0	8.896	6	2	6	2	377	41.5	6.43	11.13	2	0	1402797.055	129665.7656	10.82
P25788	Proteasome subunit alpha type-3 OS=Homo sapiens OX=9606 GN=PSMA3 PE=1 SV=2	0.005	1.957	3	1	1	1	255	28.4	5.33	1.72	1	0	53145.80469	4948.38623	10.74
P53804	E3 ubiquitin-protein ligase TTC3 OS=Homo sapiens OX=9606 GN=TTC3 PE=1 SV=2	0.012	1.503	0	1	1	1	2025	229.7	7.52	0	1	0	85720.42969	8000.288086	10.71
P09874	Poly [ADP-ribose] polymerase 1 OS=Homo sapiens OX=9606 GN=PARP1 PE=1 SV=4	0	11.251	3	4	29	4	1014	113	8.88	35.46	4	0	1423594.672	133023.0645	10.70
P62191	26S proteasome regulatory subunit 4 OS=Homo sapiens OX=9606 GN=PSMC1 PE=1 SV=1	0.002	2.321	2	1	1	1	440	49.2	6.21	1.78	1	0	24615.2793	2328.272949	10.57
O75880	Protein SCO1 homolog, mitochondrial OS=Homo sapiens OX=9606 GN=SCO1 PE=1 SV=1	0.005	1.852	3	1	1	1	301	33.8	8.88	2.26	1	0	1083508.5	102617.6328	10.56
Q5T160	Probable arginine--tRNA ligase, mitochondrial OS=Homo sapiens OX=9606 GN=RARS2 PE=1 SV=1	0	6.945	5	3	9	3	578	65.5	8.21	7.8	3	0	736303.1465	70225.03125	10.48
Q9Y618	Nuclear receptor corepressor 2 OS=Homo sapiens OX=9606 GN=NCOR2 PE=1 SV=3	0.015	1.481	0	1	1	1	2514	273.5	7.59	0	1	0	96766.25	9238.508789	10.47
Q9BZL1	Ubiquitin-like protein 5 OS=Homo sapiens OX=9606 GN=UBL5 PE=1 SV=1	0.002	3.164	12	1	2	1	73	8.5	8.44	4.75	1	0	481480.5781	47233.11914	10.19
P52292	Importin subunit alpha-1 OS=Homo sapiens OX=9606 GN=KPNA2 PE=1 SV=1	0	7.964	2	1	4	1	529	57.8	5.4	11.12	1	0	274479.748	27228.70215	10.08
P10809	60 kDa heat shock protein, mitochondrial OS=Homo sapiens OX=9606 GN=HSPD1 PE=1 SV=2	0	37.503	16	11	26	11	573	61	5.87	56.65	11	0	16186668.39	1631782.563	9.92
P22626	Heterogeneous nuclear ribonucleoproteins A2/B1 OS=Homo sapiens OX=9606 GN=HNRNPA2B1 PE=1 SV=2	0	20.112	13	3	12	3	353	37.4	8.95	10.21	3	0	326914.8613	33070.83203	9.89
P18124	60S ribosomal protein L7 OS=Homo sapiens OX=9606 GN=RPL7 PE=1 SV=1	0	3.832	6	2	3	2	248	29.2	10.65	3.48	2	0	178860.207	18267.14307	9.79
Q53FV1	ORM1-like protein 2 OS=Homo sapiens OX=9606 GN=ORMDL2 PE=1 SV=2	0	3.53	7	1	1	1	153	17.4	9.64	2.04	1	0	432794.75	44627.99219	9.70
Q9NPL8	Complex I assembly factor TIMMDC1, mitochondrial OS=Homo sapiens OX=9606 GN=TIMMDC1 PE=1 SV=2	0	8.327	7	3	20	3	285	32.2	8.5	26.48	3	0	974330.4297	101753.7212	9.58
P10599	Thioredoxin OS=Homo sapiens OX=9606 GN=TXN PE=1 SV=3	0	17.196	23	3	20	3	105	11.7	4.92	45.1	3	0	61798471.56	6784709.017	9.11
P68371	Tubulin beta-4B chain OS=Homo sapiens OX=9606 GN=TUBB4B PE=1 SV=1	0	107.317	32	12	81	1	445	49.8	4.89	177.05	12	0	9784384.188	1077911	9.08
P04075	Fructose-bisphosphate aldolase A OS=Homo sapiens OX=9606 GN=ALDOA PE=1 SV=2	0	6.584	5	3	4	3	364	39.4	8.09	2.16	3	0	74665.82813	8666.426758	8.62
P46776	60S ribosomal protein L27a OS=Homo sapiens OX=9606 GN=RPL27A PE=1 SV=2	0	5.536	7	1	3	1	148	16.6	11	8.6	1	0	14655298.5	1713025.813	8.56
P39023	60S ribosomal protein L3 OS=Homo sapiens OX=9606 GN=RPL3 PE=1 SV=2	0	10.592	6	3	8	3	403	46.1	10.18	13.07	3	0	971700.4375	114369.5352	8.50
P52272	Heterogeneous nuclear ribonucleoprotein M OS=Homo sapiens OX=9606 GN=HNRNPM PE=1 SV=3	0	38.823	13	10	44	10	730	77.5	8.7	52.53	10	0	9518988.377	1124361.001	8.47
P62244	40S ribosomal protein S15a OS=Homo sapiens OX=9606 GN=RPS15A PE=1 SV=2	0	6.821	13	2	3	2	130	14.8	10.13	6.64	2	0	1144525.375	135756.9844	8.43
Q9Y224	RNA transcription, translation and transport factor protein OS=Homo sapiens OX=9606 GN=RTRAF PE=1 SV=1	0.002	2.438	3	1	4	1	244	28.1	6.65	5.41	1	0	407014.8281	48352.44141	8.42
Q9BQG0	Myb-binding protein 1A OS=Homo sapiens OX=9606 GN=MYBBP1A PE=1 SV=2	0.002	2.378	1	1	3	1	1328	148.8	9.28	0	1	0	407014.8281	48352.44141	8.42
Q9BZE4	GTP-binding protein 4 OS=Homo sapiens OX=9606 GN=GTPBP4 PE=1 SV=3	0.005	1.916	1	1	1	1	634	73.9	9.5	1.98	1	0	53995.16797	6508.702637	8.30
P18206	Vinculin OS=Homo sapiens OX=9606 GN=VCL PE=1 SV=4	0	3.814	1	2	2	2	1134	123.7	5.66	1.86	2	0	49902.07813	6020.921387	8.29
P51153	Ras-related protein Rab-13 OS=Homo sapiens OX=9606 GN=RAB13 PE=1 SV=1	0	6.495	11	3	11	2	203	22.8	9.19	8.55	3	1	1130418.277	138233.3652	8.18
P06753	Tropomyosin alpha-3 chain OS=Homo sapiens OX=9606 GN=TPM3 PE=1 SV=2	0.002	2.187	2	1	5	1	285	32.9	4.72	7.97	1	0	982131.9688	120731.8203	8.13
P15880	40S ribosomal protein S2 OS=Homo sapiens OX=9606 GN=RPS2 PE=1 SV=2	0	11.753	10	3	5	3	293	31.3	10.24	9.93	3	0	12451418.1	1531607.25	8.13
P62701	40S ribosomal protein S4, X isoform OS=Homo sapiens OX=9606 GN=RPS4X PE=1 SV=2	0	9.403	10	3	19	3	263	29.6	10.15	26.36	3	0	6574998.719	812705.6113	8.09
P51571	Translocon-associated protein subunit delta OS=Homo sapiens OX=9606 GN=SSR4 PE=1 SV=1	0	3.519	6	1	1	1	173	19	6.15	2.38	1	0	42812.90234	5322.740723	8.04
P01008	Antithrombin-III OS=Homo sapiens OX=9606 GN=SERPINC1 PE=1 SV=1	0	5.45	3	2	8	2	464	52.6	6.71	12.11	2	0	5329125.438	667214.207	7.99
P84157	Matrix-remodeling-associated protein 7 OS=Homo sapiens OX=9606 GN=MXRA7 PE=1 SV=1	0.001	3.39	5	1	12	1	204	21.5	4.26	22.09	1	0	518682.375	65099.64453	7.97
Q8WUM4	Programmed cell death 6-interacting protein OS=Homo sapiens OX=9606 GN=PDCD6IP PE=1 SV=1	0.016	1.472	1	1	1	1	868	96	6.52	0	1	0	47008.20703	6050.107422	7.77
Q03393	6-pyruvoyl tetrahydrobiopterin synthase OS=Homo sapiens OX=9606 GN=PTS PE=1 SV=1	0	7.312	12	2	10	2	145	16.4	6.68	8.51	2	0	6187882.29	828953.9688	7.46
P68363	Tubulin alpha-1B chain OS=Homo sapiens OX=9606 GN=TUBA1B PE=1 SV=1	0	28.435	12	7	73	7	451	50.1	5.06	135.75	7	0	101671859.8	14137448.76	7.19
P62263	40S ribosomal protein S14 OS=Homo sapiens OX=9606 GN=RPS14 PE=1 SV=3	0	6.182	7	1	6	1	151	16.3	10.05	16.95	1	0	11089472	1551721.938	7.15
P13639	Elongation factor 2 OS=Homo sapiens OX=9606 GN=EEF2 PE=1 SV=4	0	11.02	4	4	19	3	858	95.3	6.83	40	4	0	8789007.527	1231697.997	7.14
O94919	Endonuclease domain-containing 1 protein OS=Homo sapiens OX=9606 GN=ENDOD1 PE=1 SV=2	0	6.915	4	2	4	2	500	55	5.71	9.54	2	0	1632678.625	230169.2188	7.09
P22234	Multifunctional protein ADE2 OS=Homo sapiens OX=9606 GN=PAICS PE=1 SV=3	0	4.173	2	1	20	1	425	47	7.23	31.73	1	0	1405797.688	198673.7061	7.08
P08865	40S ribosomal protein SA OS=Homo sapiens OX=9606 GN=RPSA PE=1 SV=4	0	5.324	4	1	2	1	295	32.8	4.87	7.08	1	0	1664598.875	238663.6875	6.97
P31153	S-adenosylmethionine synthase isoform type-2 OS=Homo sapiens OX=9606 GN=MAT2A PE=1 SV=1	0	4.502	4	2	16	2	395	43.6	6.48	23.84	2	0	2617218.227	381664.6877	6.86
O95573	Long-chain-fatty-acid--CoA ligase 3 OS=Homo sapiens OX=9606 GN=ACSL3 PE=1 SV=3	0.002	3.208	1	1	1	1	720	80.4	8.38	0	1	0	209802.4375	30721.25781	6.83
Q15181	Inorganic pyrophosphatase OS=Homo sapiens OX=9606 GN=PPA1 PE=1 SV=2	0.002	2.386	3	1	1	1	289	32.6	5.86	0	1	0	105324.1328	15455.16797	6.81
P61978	Heterogeneous nuclear ribonucleoprotein K OS=Homo sapiens OX=9606 GN=HNRNPK PE=1 SV=1	0	17.465	11	5	44	5	463	50.9	5.54	63.27	5	0	5928185.385	873944.7891	6.78
P04792	Heat shock protein beta-1 OS=Homo sapiens OX=9606 GN=HSPB1 PE=1 SV=2	0.002	2.201	5	1	3	1	205	22.8	6.4	3.92	1	0	135756.5957	20110.80249	6.75
P33992	DNA replication licensing factor MCM5 OS=Homo sapiens OX=9606 GN=MCM5 PE=1 SV=5	0	4.557	2	2	4	2	734	82.2	8.37	9.07	2	0	1469368.361	219501.9834	6.69
Q9UBV7	Beta-1,4-galactosyltransferase 7 OS=Homo sapiens OX=9606 GN=B4GALT7 PE=1 SV=1	0.002	2.678	2	1	3	1	327	37.4	8.98	3.99	1	0	1013423.625	152861.3125	6.63
P26373	60S ribosomal protein L13 OS=Homo sapiens OX=9606 GN=RPL13 PE=1 SV=4	0	6.664	9	2	4	2	211	24.2	11.65	8.14	2	0	2966506.891	448058.8438	6.62
Q9GZM8	Nuclear distribution protein nudE-like 1 OS=Homo sapiens OX=9606 GN=NDEL1 PE=1 SV=1	0.006	1.788	2	1	1	1	345	38.4	5.24	0	1	0	141943.4844	21589.18945	6.57
Q9UIW2	Plexin-A1 OS=Homo sapiens OX=9606 GN=PLXNA1 PE=1 SV=3	0	10.514	2	5	6	5	1896	210.9	6.92	6.32	5	0	402981.4805	62188.87463	6.48
P36578	60S ribosomal protein L4 OS=Homo sapiens OX=9606 GN=RPL4 PE=1 SV=5	0	7.661	4	2	4	2	427	47.7	11.06	10.92	2	0	2035796.387	321403.3125	6.33
P23458	Tyrosine-protein kinase JAK1 OS=Homo sapiens OX=9606 GN=JAK1 PE=1 SV=2	0	14.637	3	4	10	4	1154	133.2	7.55	14.63	4	0	1425028.648	225292.1406	6.33
O75122	CLIP-associating protein 2 OS=Homo sapiens OX=9606 GN=CLASP2 PE=1 SV=3	0.002	2.761	1	1	18	1	1294	141	8.4	13.68	1	0	288734.0098	46230.55969	6.25
Q8NC51	Plasminogen activator inhibitor 1 RNA-binding protein OS=Homo sapiens OX=9606 GN=SERBP1 PE=1 SV=2	0	29.413	10	3	43	3	408	44.9	8.65	126.68	3	0	7471074.633	1197866.352	6.24
Q9Y383	Putative RNA-binding protein Luc7-like 2 OS=Homo sapiens OX=9606 GN=LUC7L2 PE=1 SV=2	0	4.405	4	2	3	2	392	46.5	10.01	3.79	2	0	205812.6953	33085.59521	6.22
Q9P219	Protein Daple OS=Homo sapiens OX=9606 GN=CCDC88C PE=1 SV=3	0	16.957	3	6	16	6	2028	228.1	6.23	31.41	6	0	3801655.433	620906.9082	6.12
Q92499	ATP-dependent RNA helicase DDX1 OS=Homo sapiens OX=9606 GN=DDX1 PE=1 SV=2	0.002	2.658	1	1	6	1	740	82.4	7.23	11.89	1	0	359989.5625	59410.17773	6.06
P05141	ADP/ATP translocase 2 OS=Homo sapiens OX=9606 GN=SLC25A5 PE=1 SV=7	0	35.271	24	8	339	1	298	32.8	9.69	296.91	8	9	386625753.2	63931154.2	6.05
Q6DKI1	60S ribosomal protein L7-like 1 OS=Homo sapiens OX=9606 GN=RPL7L1 PE=1 SV=2	0.007	1.697	3	1	1	1	255	29.7	10.52	0	1	0	35919.89063	6016.572266	5.97
P49207	60S ribosomal protein L34 OS=Homo sapiens OX=9606 GN=RPL34 PE=1 SV=3	0.001	3.334	8	1	1	1	117	13.3	11.47	2.53	1	0	454451.0938	76466.48438	5.94
P25786	Proteasome subunit alpha type-1 OS=Homo sapiens OX=9606 GN=PSMA1 PE=1 SV=1	0.001	3.458	3	1	1	1	263	29.5	6.61	2.21	1	0	38517.08203	6558.914063	5.87
P07339	Cathepsin D OS=Homo sapiens OX=9606 GN=CTSD PE=1 SV=1	0.008	1.673	2	1	2	1	412	44.5	6.54	1.76	1	0	1766338.188	302723.9453	5.83
P61626	Lysozyme C OS=Homo sapiens OX=9606 GN=LYZ PE=1 SV=1	0	3.949	6	1	31	1	148	16.5	9.16	67.84	1	0	5389734.25	933691.0703	5.77
P62979	Ubiquitin-40S ribosomal protein S27a OS=Homo sapiens OX=9606 GN=RPS27A PE=1 SV=2	0	9.962	14	3	45	3	156	18	9.64	52.02	3	0	62460134.83	10826021.57	5.77
P04004	Vitronectin OS=Homo sapiens OX=9606 GN=VTN PE=1 SV=1	0.007	1.706	2	1	1	1	478	54.3	5.8	2.24	1	0	133180.0156	23223.01172	5.73
Q9Y2H1	Serine/threonine-protein kinase 38-like OS=Homo sapiens OX=9606 GN=STK38L PE=1 SV=3	0	60.459	27	11	18	9	464	54	6.81	45.41	11	0	14368120.43	2507598.11	5.73
Q5T749	Keratinocyte proline-rich protein OS=Homo sapiens OX=9606 GN=KPRP PE=1 SV=1	0.008	1.649	1	1	2	1	579	64.1	8.27	1.76	1	0	241548.1406	42657.16016	5.66
O94903	Pyridoxal phosphate homeostasis protein OS=Homo sapiens OX=9606 GN=PLPBP PE=1 SV=1	0	3.496	3	1	2	1	275	30.3	7.5	6.02	1	0	1214433.438	223600.75	5.43
Q14694	Ubiquitin carboxyl-terminal hydrolase 10 OS=Homo sapiens OX=9606 GN=USP10 PE=1 SV=2	0	5.54	2	2	6	2	798	87.1	5.31	6.02	2	0	152351.9531	28162.41992	5.41
Q14697	Neutral alpha-glucosidase AB OS=Homo sapiens OX=9606 GN=GANAB PE=1 SV=3	0.016	1.47	1	1	2	1	944	106.8	6.14	3.42	1	0	73865.74023	13728.68457	5.38
Q6KB66	Keratin, type II cytoskeletal 80 OS=Homo sapiens OX=9606 GN=KRT80 PE=1 SV=2	0	20.902	13	6	20	5	452	50.5	5.67	30.98	6	0	7669032.156	1429304.131	5.37
P08670	Vimentin OS=Homo sapiens OX=9606 GN=VIM PE=1 SV=4	0	26.465	15	9	43	7	466	53.6	5.12	63.75	9	1	2529084.281	471949.3066	5.36
Q14247	Src substrate cortactin OS=Homo sapiens OX=9606 GN=CTTN PE=1 SV=2	0	9.827	6	4	11	4	550	61.5	5.4	11.34	4	0	1884561.211	357734.7734	5.27
P26639	Threonine--tRNA ligase 1, cytoplasmic OS=Homo sapiens OX=9606 GN=TARS1 PE=1 SV=3	0.002	2.244	1	1	1	1	723	83.4	6.67	0	1	0	24317.06445	4653.681641	5.23
P32119	Peroxiredoxin-2 OS=Homo sapiens OX=9606 GN=PRDX2 PE=1 SV=5	0	17.541	27	6	21	5	198	21.9	5.97	47.11	6	0	20643135.88	3991549.547	5.17
Q8N3U4	Cohesin subunit SA-2 OS=Homo sapiens OX=9606 GN=STAG2 PE=1 SV=3	0	4.582	1	2	3	2	1231	141.2	5.43	5.22	2	0	93049.77734	18079.66992	5.15
O15127	Secretory carrier-associated membrane protein 2 OS=Homo sapiens OX=9606 GN=SCAMP2 PE=1 SV=2	0.002	2.727	4	1	1	1	329	36.6	6.1	2.67	1	0	152805.0469	29741.85742	5.14
P52597	Heterogeneous nuclear ribonucleoprotein F OS=Homo sapiens OX=9606 GN=HNRNPF PE=1 SV=3	0	6.897	5	2	4	2	415	45.6	5.58	5.68	2	0	851730.6914	168647.8281	5.05
P25789	Proteasome subunit alpha type-4 OS=Homo sapiens OX=9606 GN=PSMA4 PE=1 SV=1	0	9.297	10	3	20	3	261	29.5	7.72	31.55	3	0	12165929.91	2416075.031	5.04
P0DMV9	Heat shock 70 kDa protein 1B OS=Homo sapiens OX=9606 GN=HSPA1B PE=1 SV=1	0	70.734	21	17	160	11	641	70	5.66	191.43	17	6	130980002.7	26019139.44	5.03
P18621	60S ribosomal protein L17 OS=Homo sapiens OX=9606 GN=RPL17 PE=1 SV=3	0	7.557	10	2	10	2	184	21.4	10.17	18.56	2	0	5388752.034	1072853.362	5.02
P03951	Coagulation factor XI OS=Homo sapiens OX=9606 GN=F11 PE=1 SV=1	0.002	2.344	1	1	1	1	625	70.1	8.1	2.17	1	0	37829.76953	7635.265625	4.95
Q8ND56	Protein LSM14 homolog A OS=Homo sapiens OX=9606 GN=LSM14A PE=1 SV=3	0	4.903	2	1	3	1	463	50.5	9.52	4.67	1	0	466600.7422	94811.10742	4.92
Q15717	ELAV-like protein 1 OS=Homo sapiens OX=9606 GN=ELAVL1 PE=1 SV=2	0	3.662	3	1	2	1	326	36.1	9.17	1.75	1	0	42162.87891	8617.458984	4.89
Q969X6	U3 small nucleolar RNA-associated protein 4 homolog OS=Homo sapiens OX=9606 GN=UTP4 PE=1 SV=1	0.002	2.136	1	1	1	1	686	76.8	8.85	0	1	0	35592.08203	7324.608887	4.86

P10619	Lysosomal protective protein OS=Homo sapiens OX=9606 GN=CTSA PE=1 SV=2	0.002	2.304	2	1	1	1	480	54.4	6.61	0	1	0	36894.16406	7861.265137	4.69
P02768	Albumin OS=Homo sapiens OX=9606 GN=ALB PE=1 SV=2	0	64.629	16	11	68	11	609	69.3	6.28	162.96	11	0	1115281923	241500890	4.62
P60174	Triosephosphate isomerase OS=Homo sapiens OX=9606 GN=TPI1 PE=1 SV=4	0	13.219	13	3	6	3	249	26.7	6.9	6.84	3	0	297287.9844	65505.73438	4.54
Q7RTV0	PHD finger-like domain-containing protein 5A OS=Homo sapiens OX=9606 GN=PHF5A PE=1 SV=1	0.002	2.883	7	1	2	1	110	12.4	8.41	2.08	1	0	151272.6797	33529.67285	4.51
Q13155	Aminoacyl tRNA synthase complex-interacting multifunctional protein 2 OS=Homo sapiens OX=9606 GN=AIMP2 PE=1 SV=2	0	4.376	5	2	4	2	320	35.3	8.22	4.29	2	0	2482406.188	552885.8174	4.49
P50990	T-complex protein 1 subunit theta OS=Homo sapiens OX=9606 GN=CCT8 PE=1 SV=4	0	29.952	15	8	33	8	548	59.6	5.6	59.34	8	0	4853111.123	1101323.666	4.41
Q5BKZ1	DBIRD complex subunit ZNF326 OS=Homo sapiens OX=9606 GN=ZNF326 PE=1 SV=2	0.002	2.392	2	1	1	1	582	65.6	5.15	2.27	1	0	38175.43359	8805.227539	4.34
P22102	Trifunctional purine biosynthetic protein adenosine-3 OS=Homo sapiens OX=9606 GN=GART PE=1 SV=1	0	5.088	2	2	2	2	1010	107.7	6.7	3.97	2	0	448180.2598	104852.3359	4.27
P78371	T-complex protein 1 subunit beta OS=Homo sapiens OX=9606 GN=CCT2 PE=1 SV=4	0	15.576	8	5	21	5	535	57.5	6.46	23.16	5	0	3248278.813	759961.2842	4.27
P33527	Multidrug resistance-associated protein 1 OS=Homo sapiens OX=9606 GN=ABCC1 PE=1 SV=3	0	11.29	2	3	4	3	1531	171.5	7.11	8.44	3	0	158413.6406	37094.62109	4.27
P01024	Complement C3 OS=Homo sapiens OX=9606 GN=C3 PE=1 SV=2	0.005	1.856	0	1	3	1	1663	187	6.4	3.9	1	0	374408.4531	88482.70313	4.23
P35580	Myosin-10 OS=Homo sapiens OX=9606 GN=MYH10 PE=1 SV=3	0	18.721	3	7	20	6	1976	228.9	5.54	34.07	7	0	4874190.725	1184379.246	4.12
P29508	Serpin B3 OS=Homo sapiens OX=9606 GN=SERPINB3 PE=1 SV=2	0	5.203	4	2	13	1	390	44.5	6.81	24.31	2	1	3358938.172	816938.3359	4.11
P56182	Ribosomal RNA processing protein 1 homolog A OS=Homo sapiens OX=9606 GN=RRP1 PE=1 SV=1	0	3.842	2	1	1	1	461	52.8	9.33	2.25	1	0	44047.61328	10809.43652	4.07
Q00839	Heterogeneous nuclear ribonucleoprotein U OS=Homo sapiens OX=9606 GN=HNRNPU PE=1 SV=6	0.007	1.777	1	1	7	1	825	90.5	6	6.81	1	0	1322119.617	331181.1719	3.99
Q14642	Inositol polyphosphate-5-phosphatase A OS=Homo sapiens OX=9606 GN=INPP5A PE=1 SV=1	0	4.009	2	1	1	1	412	47.8	7.03	2.04	1	0	33908.02734	8565.787109	3.96
P29401	Transketolase OS=Homo sapiens OX=9606 GN=TKT PE=1 SV=3	0.002	2.788	1	1	2	1	623	67.8	7.66	4.19	1	0	121667.2773	30763.6377	3.95
Q15427	Splicing factor 3B subunit 4 OS=Homo sapiens OX=9606 GN=SF3B4 PE=1 SV=1	0.002	2.639	2	1	4	1	424	44.4	8.56	3.67	1	0	99637.6875	25436.57861	3.92
P23528	Cofilin-1 OS=Homo sapiens OX=9606 GN=CFL1 PE=1 SV=3	0.001	3.241	4	1	10	1	166	18.5	8.09	14	1	0	2546436.844	656596.9707	3.88
Q8N1N4	Keratin, type II cytoskeletal 78 OS=Homo sapiens OX=9606 GN=KRT78 PE=1 SV=2	0	24.907	12	8	43	5	520	56.8	6.02	58.62	8	1	3317207.078	874214.5566	3.79
Q9H0E2	Toll-interacting protein OS=Homo sapiens OX=9606 GN=TOLLIP PE=1 SV=1	0.007	1.69	3	1	1	1	274	30.3	5.97	0	1	0	99204.30469	26251.96289	3.78
Q7Z353	Highly divergent homeobox OS=Homo sapiens OX=9606 GN=HDX PE=1 SV=1	0	8.71	4	3	7	3	690	77.2	5.87	11.74	3	0	650224.2031	177875.1875	3.66
Q9HAV0	Guanine nucleotide-binding protein subunit beta-4 OS=Homo sapiens OX=9606 GN=GNB4 PE=1 SV=3	0	10.222	9	4	18	3	340	37.5	6	31.82	4	1	2411299.773	660504.0625	3.65
P68431	Histone H3.1 OS=Homo sapiens OX=9606 GN=H3C1 PE=1 SV=2	0	4.663	13	2	9	2	136	15.4	11.12	13.58	2	0	28916674.66	8133167.072	3.56
P52732	Kinesin-like protein KIF11 OS=Homo sapiens OX=9606 GN=KIF11 PE=1 SV=2	0	23.527	7	9	49	9	1056	119.1	5.64	88.1	9	0	5578590.621	1599723.362	3.49
Q6P3X3	Tetratricopeptide repeat protein 27 OS=Homo sapiens OX=9606 GN=TTC27 PE=1 SV=1	0	5.388	2	2	6	2	843	96.6	5.59	11.21	2	0	1824393.965	529141.2422	3.45
Q15054	DNA polymerase delta subunit 3 OS=Homo sapiens OX=9606 GN=POLD3 PE=1 SV=2	0.002	2.26	2	1	1	1	466	51.4	9.35	0	1	0	24893.6582	7280.422363	3.42
Q9BRS2	Serine/threonine-protein kinase RIO1 OS=Homo sapiens OX=9606 GN=RIOK1 PE=1 SV=2	0	17.512	7	5	56	5	568	65.5	6.19	57.02	5	0	11513048.39	3398897.332	3.39
Q15208	Serine/threonine-protein kinase 38 OS=Homo sapiens OX=9606 GN=STK38 PE=1 SV=1	0	94.892	38	15	49	13	465	54.2	7.15	116.13	15	1	171591649.5	50737118	3.38
Q16643	Drebrin OS=Homo sapiens OX=9606 GN=DBN1 PE=1 SV=4	0	12.435	5	4	20	4	649	71.4	4.45	29.38	4	0	1633213.232	515734.5146	3.17
O14744	Protein arginine N-methyltransferase 5 OS=Homo sapiens OX=9606 GN=PRMT5 PE=1 SV=4	0	167.964	33	20	220	20	637	72.6	6.29	404.12	20	0	1176983418	373743482.1	3.15
O00268	Transcription initiation factor TFIID subunit 4 OS=Homo sapiens OX=9606 GN=TAF4 PE=1 SV=2	0	10.614	3	4	19	4	1085	110	9.94	23.39	4	0	1238251.43	398079.5266	3.11
P62847	40S ribosomal protein S24 OS=Homo sapiens OX=9606 GN=RPS24 PE=1 SV=1	0.002	2.688	6	1	3	1	133	15.4	10.78	4.03	1	0	86639.78906	28597.01953	3.03
Q9UMS4	Pre-mRNA-processing factor 19 OS=Homo sapiens OX=9606 GN=PRPF19 PE=1 SV=1	0.007	1.774	1	1	5	1	504	55.1	6.61	9.95	1	0	334370.3281	115089.5234	2.91
Q9NYF8	Bcl-2-associated transcription factor 1 OS=Homo sapiens OX=9606 GN=BCLAF1 PE=1 SV=2	0	24.958	6	6	35	6	920	106.1	9.98	62.1	6	0	18060557.35	6249806.688	2.89
Q9Y657	Spindlin-1 OS=Homo sapiens OX=9606 GN=SPIN1 PE=1 SV=3	0	8.785	9	2	2	2	262	29.6	6.96	5.38	2	0	1966160.375	725463.625	2.71
Q9HDC5	Junctophilin-1 OS=Homo sapiens OX=9606 GN=JPH1 PE=1 SV=2	0	5.361	2	1	4	1	661	71.6	9.32	8.39	1	0	458016.3359	169276.0313	2.71
Q02413	Desmoglein-1 OS=Homo sapiens OX=9606 GN=DSG1 PE=1 SV=2	0	18.966	4	5	30	5	1049	113.7	5.03	58.96	5	0	10498707.39	3902221.816	2.69
P55769	NHP2-like protein 1 OS=Homo sapiens OX=9606 GN=SNU13 PE=1 SV=3	0	3.57	7	1	9	1	128	14.2	8.46	16.06	1	0	478595.4277	178403.8945	2.68
Q01518	Adenylyl cyclase-associated protein 1 OS=Homo sapiens OX=9606 GN=CAP1 PE=1 SV=5	0.011	1.561	2	1	1	1	475	51.9	8.06	0	1	0	22362.32813	8405.287109	2.66
P62899	60S ribosomal protein L31 OS=Homo sapiens OX=9606 GN=RPL31 PE=1 SV=1	0.002	2.621	7	1	1	1	125	14.5	10.54	2.83	1	0	832465	316237.875	2.63
Q92520	Protein FAM3C OS=Homo sapiens OX=9606 GN=FAM3C PE=1 SV=1	0.002	2.5	4	1	1	1	227	24.7	8.29	1.84	1	0	19525.63281	7460.873047	2.62
P48594	Serpin B4 OS=Homo sapiens OX=9606 GN=SERPINB4 PE=1 SV=2	0	6.29	4	2	18	1	390	44.8	6.21	23.96	2	0	152469.7148	58855	2.59
P14923	Junction plakoglobin OS=Homo sapiens OX=9606 GN=JUP PE=1 SV=3	0	17.095	6	5	27	5	745	81.7	6.14	37.71	5	0	3664029.666	1440462.754	2.54
P31943	Heterogeneous nuclear ribonucleoprotein H OS=Homo sapiens OX=9606 GN=HNRNPH1 PE=1 SV=4	0	9.759	6	2	11	1	449	49.2	6.3	26.2	2	0	4116189.137	1643981.276	2.50
P28072	Proteasome subunit beta type-6 OS=Homo sapiens OX=9606 GN=PSMB6 PE=1 SV=4	0	7.555	13	3	6	3	239	25.3	4.92	4.7	3	0	482278.75	194815.0156	2.48
Q8NHQ1	Centrosomal protein of 70 kDa OS=Homo sapiens OX=9606 GN=CEP70 PE=1 SV=2	0	17.016	7	6	14	6	597	69.7	5.71	20.98	6	0	817268.5215	330476.0161	2.47
Q9H061	Transmembrane protein 126A OS=Homo sapiens OX=9606 GN=TMEM126A PE=1 SV=1	0.002	2.3	5	1	1	1	195	21.5	9.26	2.42	1	0	760570.5625	307711.1563	2.47
Q5W0B1	ORC ubiquitin ligase 1 OS=Homo sapiens OX=9606 GN=OBI1 PE=1 SV=1	0	10.64	4	3	26	3	726	81.1	5.72	47.13	3	0	2364691.695	984013.5566	2.40
P84090	Enhancer of rudimentary homolog OS=Homo sapiens OX=9606 GN=ERH PE=1 SV=1	0	22.224	26	3	14	3	104	12.3	5.92	45.7	3	0	22897282.59	9787673.5	2.34
P31025	Lipocalin-1 OS=Homo sapiens OX=9606 GN=LCN1 PE=1 SV=1	0	5.275	12	2	3	2	176	19.2	5.58	6.4	2	0	1848565.609	792730.6475	2.33
Q9BUA3	Spindlin interactor and repressor of chromatin-binding protein OS=Homo sapiens OX=9606 GN=SPINDOC PE=1 SV=3	0.002	2.551	2	1	15	1	381	41	5.01	15.82	1	0	1977110.414	853411.1339	2.32
P26641	Elongation factor 1-gamma OS=Homo sapiens OX=9606 GN=EEF1G PE=1 SV=3	0	9.938	7	4	10	4	437	50.1	6.67	17.04	4	0	2902401.57	1255911.167	2.31
Q6E0U4	Dermokine OS=Homo sapiens OX=9606 GN=DMKN PE=1 SV=3	0.017	1.456	1	1	3	1	476	47.1	7.28	3.54	1	0	165501.5742	72569.39648	2.28
Q15459	Splicing factor 3A subunit 1 OS=Homo sapiens OX=9606 GN=SF3A1 PE=1 SV=1	0	10.436	4	3	6	3	793	88.8	5.22	10.62	3	0	153881.3906	67515.10938	2.28
O75688	Protein phosphatase 1B OS=Homo sapiens OX=9606 GN=PPM1B PE=1 SV=1	0	148.921	34	16	189	13	479	52.6	5.05	376.05	16	4	360235004.3	159192420.4	2.26
P08621	U1 small nuclear ribonucleoprotein 70 kDa OS=Homo sapiens OX=9606 GN=SNRNP70 PE=1 SV=2	0	5.833	4	2	11	2	437	51.5	9.94	12.17	2	0	846021.3359	376027.7744	2.25
Q16875	6-phosphofructo-2-kinase/fructose-2,6-bisphosphatase 3 OS=Homo sapiens OX=9606 GN=PFKFB3 PE=1 SV=1	0	48.009	20	11	45	9	520	59.6	8.21	94.31	11	2	104212120.6	46538670.01	2.24
P02656	Apolipoprotein C-III OS=Homo sapiens OX=9606 GN=APOC3 PE=1 SV=1	0	12.257	16	1	7	1	99	10.8	5.41	27.56	1	0	5291843	2391364.75	2.21
P35579	Myosin-9 OS=Homo sapiens OX=9606 GN=MYH9 PE=1 SV=4	0	36.626	4	9	26	7	1960	226.4	5.6	34.25	9	2	3346294.215	1519117.351	2.20
P13591	Neural cell adhesion molecule 1 OS=Homo sapiens OX=9606 GN=NCAM1 PE=1 SV=3	0.001	3.423	1	1	3	1	858	94.5	4.87	1.7	1	0	120826.6914	55724.59766	2.17
P49368	T-complex protein 1 subunit gamma OS=Homo sapiens OX=9606 GN=CCT3 PE=1 SV=4	0	7.275	4	2	15	2	545	60.5	6.49	25.37	2	0	475813.6641	221100.5664	2.15
P08727	Keratin, type I cytoskeletal 19 OS=Homo sapiens OX=9606 GN=KRT19 PE=1 SV=4	0	17.381	13	6	44	1	400	44.1	5.14	82.96	6	0	33694.31641	15709.71484	2.14
P13645	Keratin, type I cytoskeletal 10 OS=Homo sapiens OX=9606 GN=KRT10 PE=1 SV=6	0	140.094	38	22	185	18	584	58.8	5.21	443.45	22	4	1211094747	583034867.4	2.08
P05089	Arginase-1 OS=Homo sapiens OX=9606 GN=ARG1 PE=1 SV=2	0	6.634	8	3	23	3	322	34.7	7.21	28.76	3	0	4634097.617	2233617.213	2.07
P07910	Heterogeneous nuclear ribonucleoproteins C1/C2 OS=Homo sapiens OX=9606 GN=HNRNPC PE=1 SV=4	0	7.008	8	3	27	3	306	33.7	5.08	43.31	3	0	3094696.523	1540148.533	2.01
Q9C0H9	SRC kinase signaling inhibitor 1 OS=Homo sapiens OX=9606 GN=SRCIN1 PE=1 SV=4	0.008	1.642	1	1	2	1	1183	127	9.38	3.81	1	0	163229.5859	84329.07422	1.94
P35813	Protein phosphatase 1A OS=Homo sapiens OX=9606 GN=PPM1A PE=1 SV=1	0	35.462	13	5	37	2	382	42.4	5.36	90.83	5	0	1539635.188	812735.5	1.89
P12004	Proliferating cell nuclear antigen OS=Homo sapiens OX=9606 GN=PCNA PE=1 SV=1	0	7.484	6	2	13	2	261	28.8	4.69	5.34	2	0	558724.9219	296363.7605	1.89
P62316	Small nuclear ribonucleoprotein Sm D2 OS=Homo sapiens OX=9606 GN=SNRPD2 PE=1 SV=1	0	6.18	14	2	8	2	118	13.5	9.91	13.62	2	0	1363018.953	726665.6875	1.88
P98164	Low-density lipoprotein receptor-related protein 2 OS=Homo sapiens OX=9606 GN=LRP2 PE=1 SV=3	0.019	1.437	0	1	2	1	4655	521.6	5.08	1.66	1	0	700251.4219	393211.9727	1.78
P19823	Inter-alpha-trypsin inhibitor heavy chain H2 OS=Homo sapiens OX=9606 GN=ITIH2 PE=1 SV=2	0	4.718	2	2	9	2	946	106.4	6.86	16.15	2	0	6682155.281	3800159.461	1.76
O00571	ATP-dependent RNA helicase DDX3X OS=Homo sapiens OX=9606 GN=DDX3X PE=1 SV=3	0	9.594	3	2	14	2	662	73.2	7.18	35.28	2	0	1670494.156	973487.9102	1.72
P19338	Nucleolin OS=Homo sapiens OX=9606 GN=NCL PE=1 SV=3	0	4.706	2	2	4	2	710	76.6	4.7	3.91	2	0	163530.375	97240.16699	1.68
Q9BQA1	Methylosome protein 50 OS=Homo sapiens OX=9606 GN=WDR77 PE=1 SV=1	0	33.538	15	4	15	4	342	36.7	5.17	42.91	4	0	161315970	96456667.25	1.67
Q9NZT1	Calmodulin-like protein 5 OS=Homo sapiens OX=9606 GN=CALML5 PE=1 SV=2	0	13.755	31	3	8	3	146	15.9	4.44	15.91	3	0	3357163.668	2063963.625	1.63
P13647	Keratin, type II cytoskeletal 5 OS=Homo sapiens OX=9606 GN=KRT5 PE=1 SV=3	0	130.548	42	28	166	20	590	62.3	7.74	352.78	28	7	397294724.2	246334032.1	1.61
P01040	Cystatin-A OS=Homo sapiens OX=9606 GN=CSTA PE=1 SV=1	0	30.555	57	5	18	5	98	11	5.5	19.87	5	0	5126711.41	3222946.246	1.59
P35908	Keratin, type II cytoskeletal 2 epidermal OS=Homo sapiens OX=9606 GN=KRT2 PE=1 SV=2	0	230.566	43	26	227	18	639	65.4	8	604.33	26	2	561432863	358204962.4	1.57
O43324	Eukaryotic translation elongation factor 1 epsilon-1 OS=Homo sapiens OX=9606 GN=EEF1E1 PE=1 SV=1	0	8.169	11	2	6	2	174	19.8	8.54	10.65	2	0	426813.375	275711.0625	1.55
P01876	Immunoglobulin heavy constant alpha 1 OS=Homo sapiens OX=9606 GN=IGHA1 PE=1 SV=2	0	5.708	5	2	10	2	353	37.6	6.51	18.11	2	0	821006.3867	548154.7422	1.50
Q96QA5	Gasdermin-A OS=Homo sapiens OX=9606 GN=GSDMA PE=1 SV=4	0	5.436	4	2	14	2	445	49.3	5.29	19.83	2	0	526827.5938	360183.6289	1.46
P54105	Methylosome subunit pICln OS=Homo sapiens OX=9606 GN=CLNS1A PE=1 SV=1	0	28.043	13	2	18	2	237	26.2	4.11	57.93	2	0	10923036.88	7608161.398	1.44
Q9HD45	Transmembrane 9 superfamily member 3 OS=Homo sapiens OX=9606 GN=TM9SF3 PE=1 SV=2	0.005	1.831	1	1	2	1	589	67.8	7.21	3.98	1	0	857906.3555	598574.875	1.43
Q15942	Zyxin OS=Homo sapiens OX=9606 GN=ZYX PE=1 SV=1	0	13.803	6	4	40	4	572	61.2	6.67	78.64	4	0	8816480.5	6157924.547	1.43
Q9Y4Z0	U6 snRNA-associated Sm-like protein LSm4 OS=Homo sapiens OX=9606 GN=LSM4 PE=1 SV=1	0.002	2.536	6	1	3	1	139	15.3	9.99	0	1	0	230628.4219	161349.7539	1.43
O60825	6-phosphofructo-2-kinase/fructose-2,6-bisphosphatase 2 OS=Homo sapiens OX=9606 GN=PFKFB2 PE=1 SV=2	0	20.102	12	7	23	5	505	58.4	8.38	42.19	7	0	6464053.666	4678180.582	1.38
P06396	Gelsolin OS=Homo sapiens OX=9606 GN=GSN PE=1 SV=1	0	16.445	6	5	21	5	782	85.6	6.28	44.15	5	0	1578031.496	1159254.191	1.36
Q96HS1	Serine/threonine-protein phosphatase PGAM5, mitochondrial OS=Homo sapiens OX=9606 GN=PGAM5 PE=1 SV=2	0	8.005	11	3	3	3	289	32	8.68	2.83	3	0	17740451.25	13407380	1.32
P15924	Desmoplakin OS=Homo sapiens OX=9606 GN=DSP PE=1 SV=3	0	62.758	7	23	112	23	2871	331.6	6.81	178.16	23	0	17300267.74	13150695.22	1.32
P02452	Collagen alpha-1(I) chain OS=Homo sapiens OX=9606 GN=COL1A1 PE=1 SV=5	0	7.495	2	3	17	3	1464	138.9	5.8	27	3	0	1289481.137	996682.3301	1.29
P60709	Actin, cytoplasmic 1 OS=Homo sapiens OX=9606 GN=ACTB PE=1 SV=1	0	146.293	37	13	196	4	375	41.7	5.48	440.35	13	12	880853102.9	690792637.6	1.28
P05109	Protein S100-A8 OS=Homo sapiens OX=9606 GN=S100A8 PE=1 SV=1	0.005	1.911	8	1	5	1	93	10.8	7.03	10.59	1	0	3940650.375	3180520.25	1.24
P37802	Transgelin-2 OS=Homo sapiens OX=9606 GN=TAGLN2 PE=1 SV=3	0	5.717	12	2	3	2	199	22.4	8.25	1.68	2	0	225852.2969	183719.5156	1.23
P47756	F-actin-capping protein subunit beta OS=Homo sapiens OX=9606 GN=CAPZB PE=1 SV=4	0	5.375	3	1	10	1	277	31.3	5.59	9.24	1	0	485025.3555	408485.4897	1.19
P02649	Apolipoprotein E OS=Homo sapiens OX=9606 GN=APOE PE=1 SV=1	0	5.582	5	2	13	2	317	36.1	5.73	17.11	2	0	2607813.297	2285272.336	1.14
Q9UHR6	Zinc finger HIT domain-containing protein 2 OS=Homo sapiens OX=9606 GN=ZNHIT2 PE=1 SV=1	0	9.82	7	3	13	3	403	42.9	5.99	22.65	3	0	408250278	359550176	1.14
P07996	Thrombospondin-1 OS=Homo sapiens OX=9606 GN=THBS1 PE=1 SV=2	0.001	3.324	1	1	1	1	1170	129.3	4.94	2.5	1	0	280682.9688	261654.7813	1.07
Q01469	Fatty acid-binding protein 5 OS=Homo sapiens OX=9606 GN=FABP5 PE=1 SV=3	0	11.301	19	3	18	3	135	15.2	7.01	37.02	3	0	3756735.688	3507457.629	1.07
Q9UGL9	Cysteine-rich C-terminal protein 1 OS=Homo sapiens OX=9606 GN=CRCT1 PE=1 SV=1	0.002	2.447	11	1	2	1	99	9.7	8.66	1.72	1	0	57799.96875	54063.80859	1.07
Q13867	Bleomycin hydrolase OS=Homo sapiens OX=9606 GN=BLMH PE=1 SV=1	0.002	2.135	2	1	2	1	455	52.5	6.27	2.12	1	0	46041.40625	43482.625	1.06
