# Supplementary material for: Neurochondrin promotes U5 snRNP maturation by regulating AAR2 release from PRPF8
Source: Nucleic Acids Res. 2026 Jul 7;54(13):gkag685. doi: 10.1093/nar/gkag685 (PMC13338715; doi:10.1093/nar/gkag685)
Supplement: gkag685_Supplemental_Files [file gkag685_supplemental_files.zip › Supplementary Figures (gkag685).pdf]

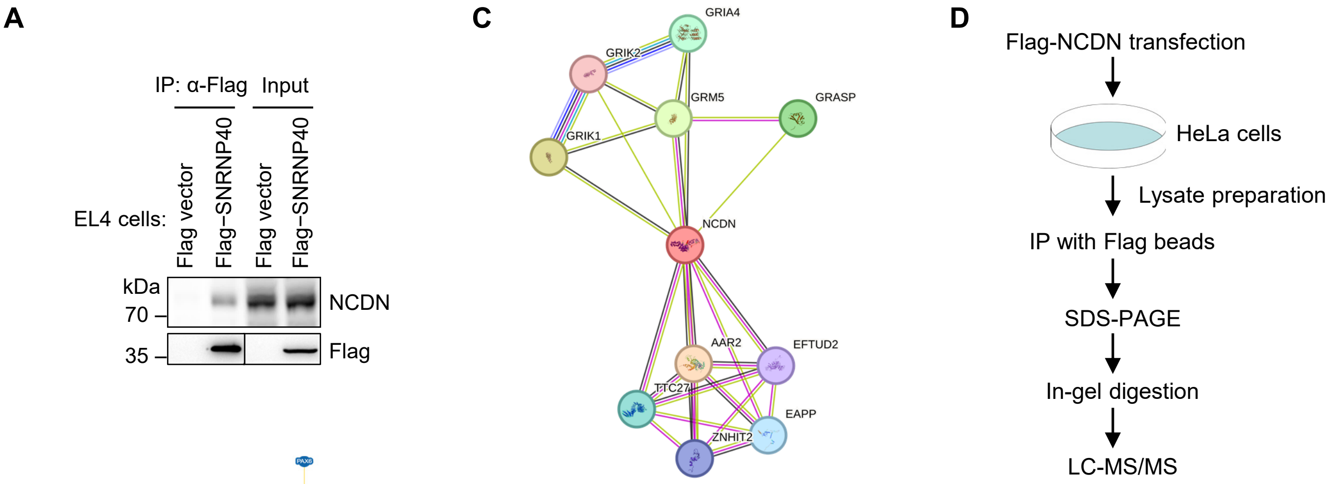


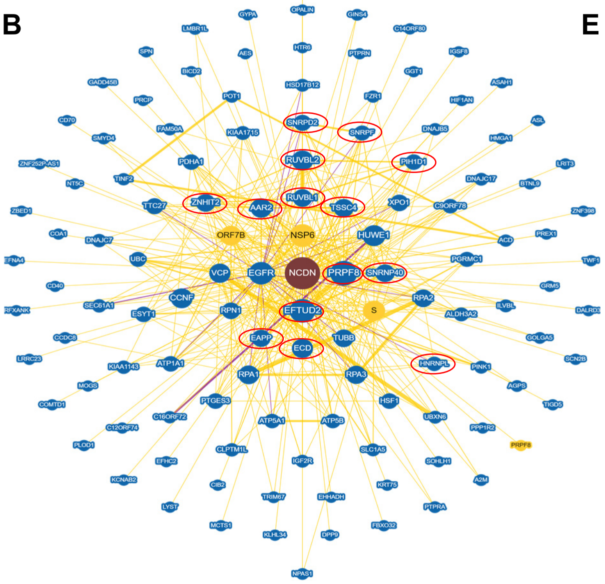


E

| Protein | #PSMs | Complex | Protein | #PSMs | Complex |
|---|---|---|---|---|---|
| NCDN | 453 | U5 assembly factor | HNRNPH2 | 10 | hnRNP |
| HSPA8 | 149 | PRP19 complex | SNRPD2 | 8 | Sm |
| PRPF8 | 86 | U5 snRNP | HNRNPU | 7 | hnRNP |
| HNRNPM | 44 | hnRNP | RUVBL2 | 5 | U5 assembly factor |
| HNRNPK | 44 | hnRNP | PRPF19 | 5 | PRP19 complex |
| EFTUD2 | 27 | U5 snRNP | HNRNPF | 4 | hnRNP |
| HNRNPC | 27 | hnRNP | SF3B4 | 4 | U2 snRNP |
| SF3B6 | 26 | U2 snRNP | U2AF1 | 4 | U2 snRNP associated |
| PRPF31 | 23 | U4/U6 snRNP | SNRPC | 3 | U1 snRNP |
| SFPQ | 23 | Splicing factor | HNRNPL | 3 | hnRNP |
| AAR2 | 18 | U5 assembly factor | SF3B2 | 3 | U2 snRNP |
| ECD | 15 | U5 assembly factor | HNRNPA1 | 3 | hnRNP |
| SNRNP200 | 17 | U5 snRNP | RUVBL1 | 2 | U5 assembly factor |
| RBM10 | 13 | Recruited at A complex | CDC5L | 1 | PRP19 complex |
| HNRNPA2B1 | 12 | hnRNP | DDX20 | 1 | GEM |
| HNRNPH1 | 11 | hnRNP | PRPF6 | 1 | U5 snRNP |
| SNRNP70 | 11 | U1 snRNP | HNRNPUL2 | 1 | hnRNP |

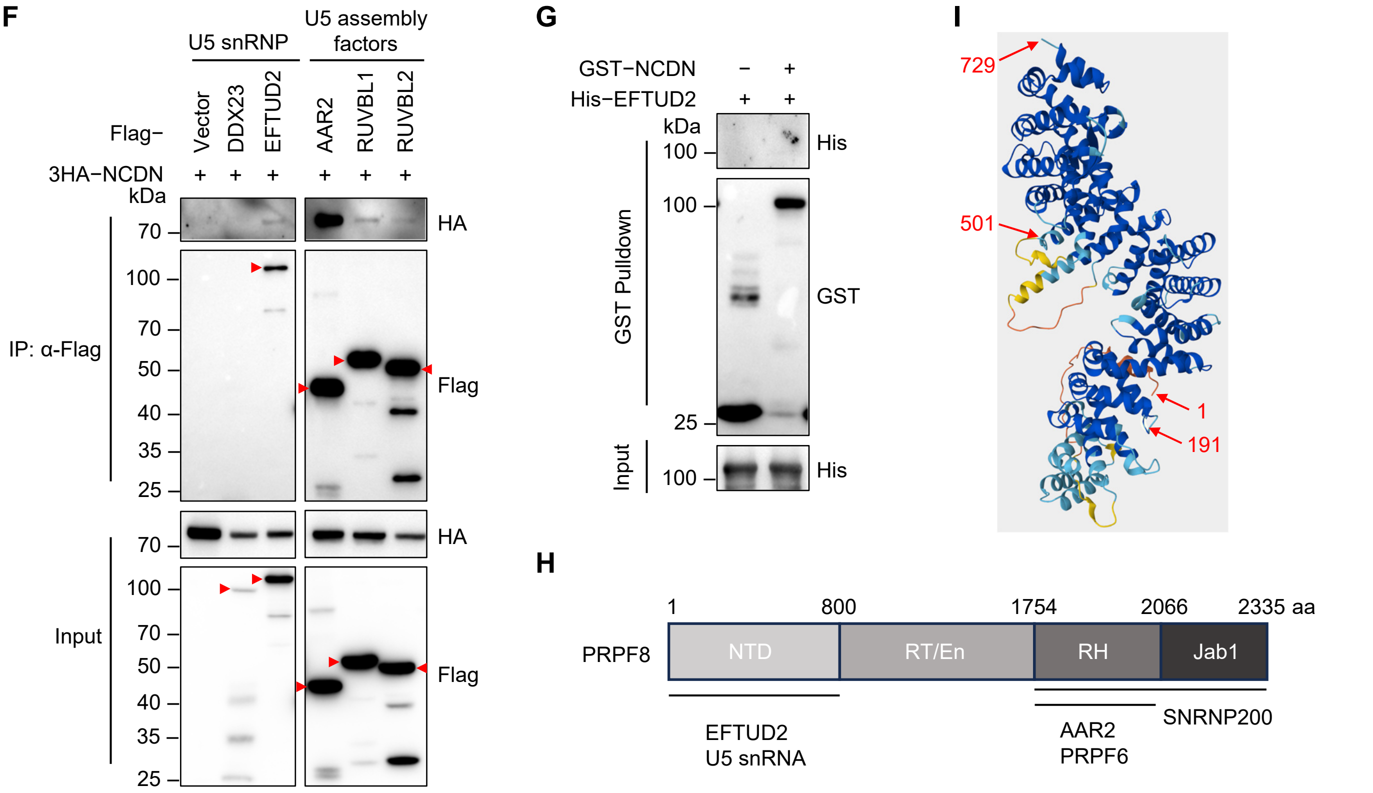


**Fig. S1 | NCDN associates with spliceosomal components.**

**Fig. S1 | NCDN associates with spliceosomal components.**

**(A)** EL4 cells were infected with lentivirus expressing Flag (vector) or Flag-tagged SNRNP40. Cell lysates were subjected to immunoprecipitation (IP) using anti-Flag beads, and bound proteins were analyzed by immunoblotting (IB) with the indicated antibodies.

**(B)** Network of NCDN-interacting candidates retrieved from the BioGRID database (https://thebiogrid.org). Splicing-related proteins are highlighted with red circles.

**(C)** Protein–protein interaction network of NCDN-interacting proteins generated using the STRING database (version 12.0) (https://cn.string-db.org).

**(D)** Schematic workflow for the identification of NCDN-interacting proteins by co-immunoprecipitation followed by mass spectrometry (Co−IP/MS).

**(E)** Subset of splicing-related proteins identified in NCDN immunoprecipitates by Co−IP/MS. PSMs, peptide spectrum matches.

(**F**) HEK293T cells were co-transfected with 3×HA-tagged NCDN and individual Flag-tagged spliceosome components. Cell lysates were subjected to anti-Flag IP and analyzed by immunoblotting with the indicated antibodies. The red triangle marks the position of the overexpressed protein band.

(**G**) Bacterially expressed GST or GST-tagged NCDN proteins were incubated with purified His-tagged EFTUD2, followed by GST pulldown. Bound proteins were detected by immunoblotting using for anti-His and anti-GST antibodies.

(**H**) Schematic representation of PRPF8 domains, including the N-terminal domain (NTD), reverse transcriptase–like/endonuclease–like domain (RT/En), RNase H–like domain (RH), and Jab1/MPN domain (Jab1).

(**I**) Predicted three-dimensional structure of human NCDN generated using AlphaFold3.

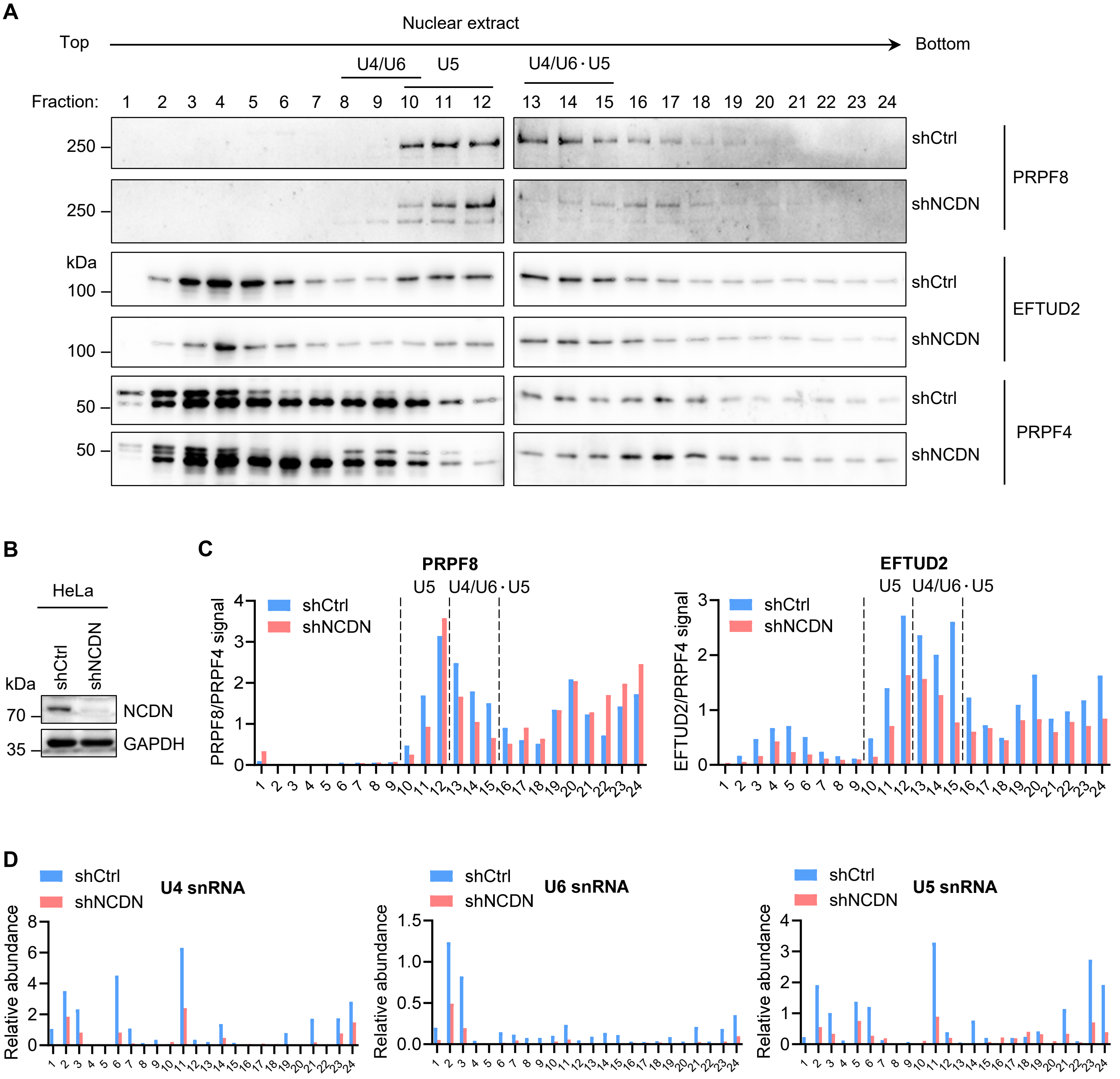


**Fig. S2 | NCDN deficiency hinders U5 snRNP biogenesis.**

(**A**) Glycerol gradient centrifugation (10-30%) of nuclear extracts from control (shCtrl) and NCDN-knockdown (shNCDN) HeLa cells, followed by immunoblotting with the indicated antibodies to determine the distribution and relative abundance across the gradient.

(**B**) Immunoblot analysis of NCDN expression in lysates of HeLa cells infected with lentivirus encoding control shRNA (shCtrl) or shRNA targeting NCDN (shNCDN).

(**C**) Glycerol gradient fractions were analyzed by immunoblotting, and the intensity of each protein band was measured using ImageJ. For each fraction, the intensity of the indicated protein was normalized to the intensity of PRPF4 within the same fraction, which served as an internal control due to its stable expression across different samples. The graph shows the distribution of indicated proteins in representative experiment.

(**D**) Nuclear extracts from U251 cells were fractionated by 10–30% glycerol gradient centrifugation. RNA was extracted from each fraction, and equal volumes of RNA from each fraction were subjected to reverse transcription. The levels of snRNAs were quantified by RT–qPCR and are presented as $2^{-CT}$ values.

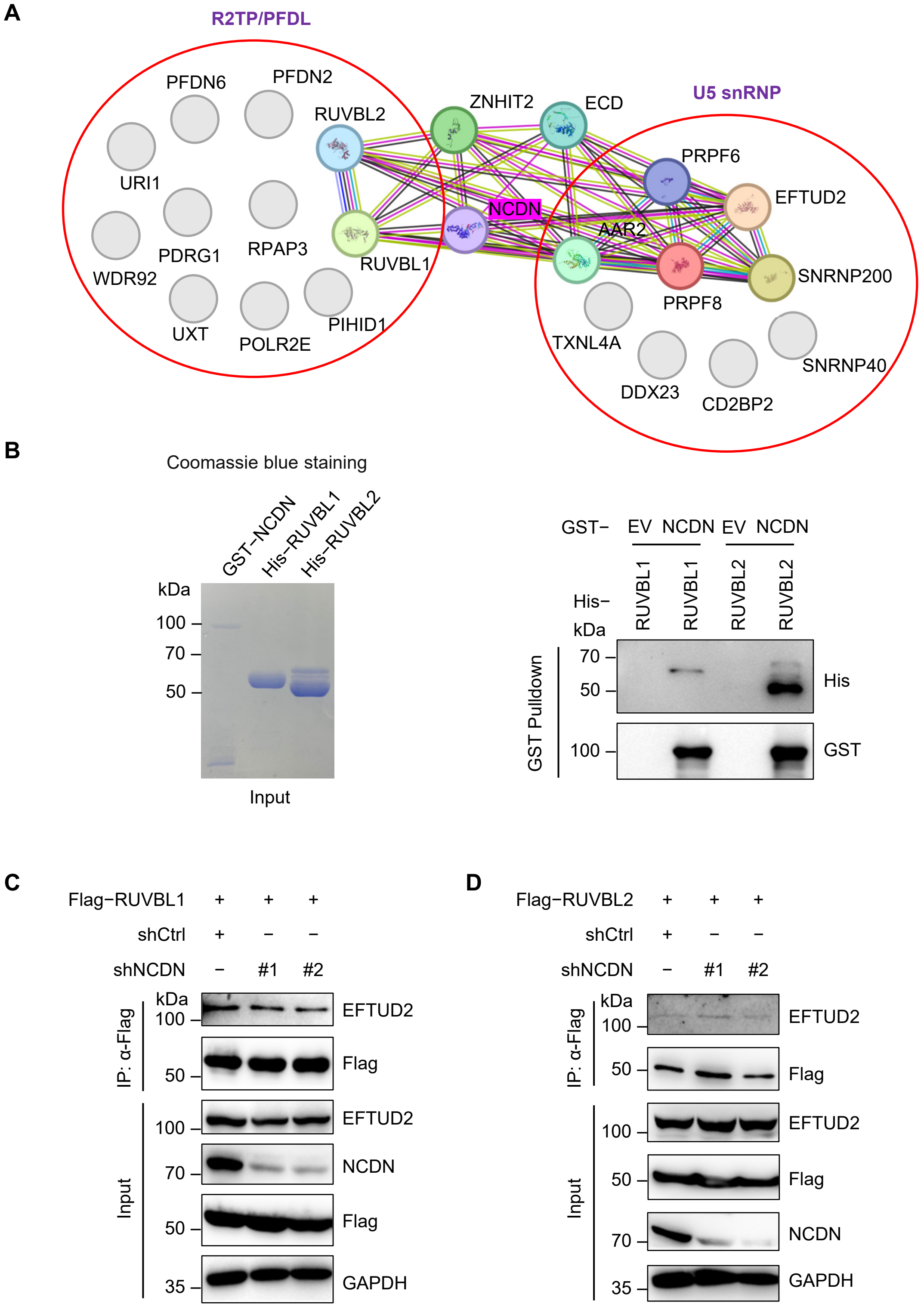


**Fig. S3 | NCDN does not affect R2TP complex recruitment to U5 snRNP.**

(**A**) Interaction network centered on NCDN. Colored nodes represent proteins identified in NCDN purifications. White nodes represent known protein complex subunits that were not detected.

(**B**) Bacterially purified GST or GST-NCDN proteins were incubated with His-RUVBL1 or His-RUVBL2, followed by GST pulldown and immunoblotting with anti-His and anti-GST antibodies.

(**C–D**) HEK293T cells expressing FLAG-RUVBL1 **(C)** and FLAG-RUVBL2 **(D)** were infected with control or NCDN-targeting shRNAs. Semi-endogenous Flag IPs were analyzed by immunoblotting for the indicated endogenous proteins.

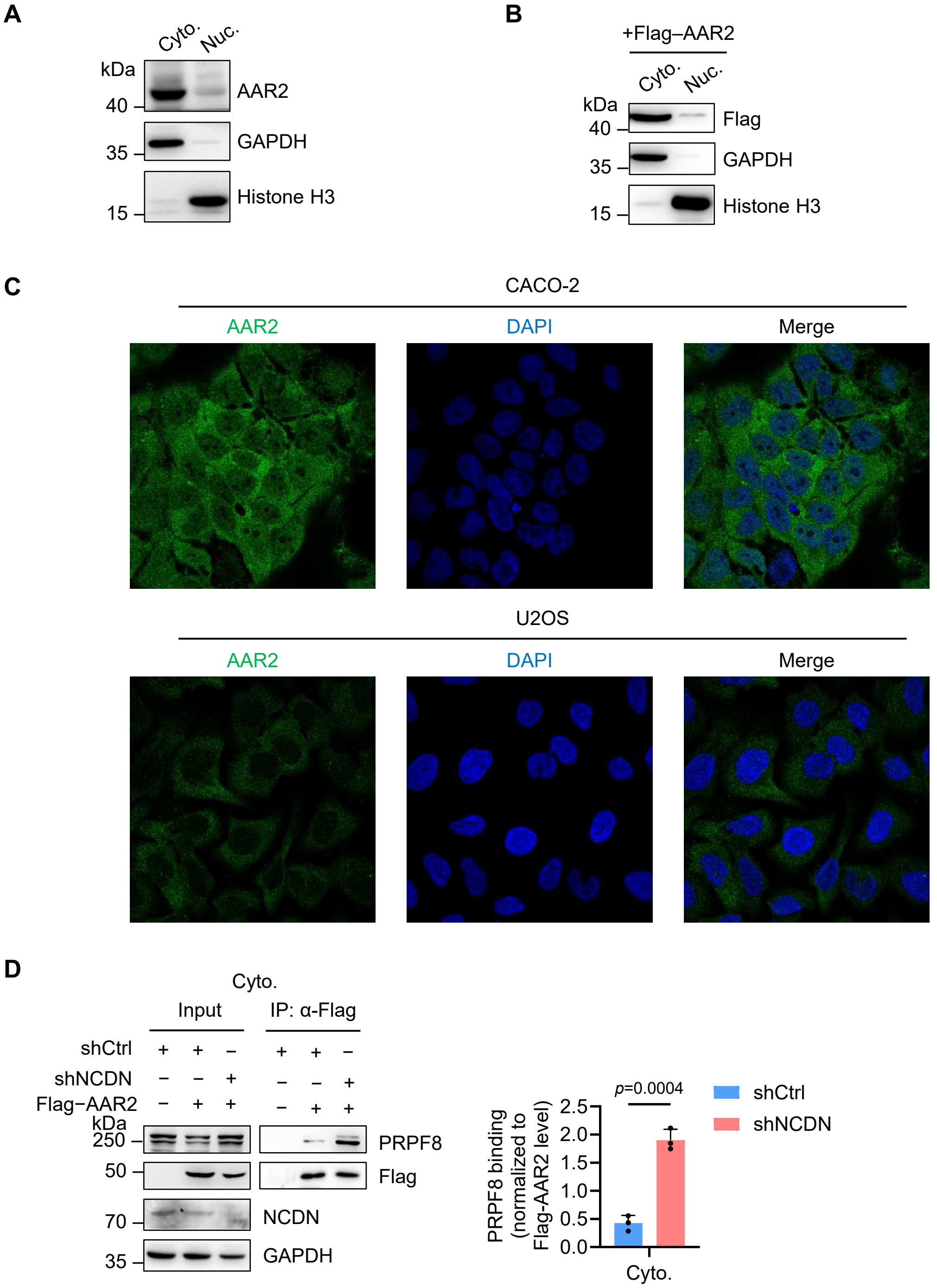


**Fig. S4 | AAR2 is mainly located in the cytoplasm.**

(**A**) U251 cells were fractionated into cytoplasmic (Cyto.) and nuclear (Nuc.) fractions, followed by immunoblotting to assess the subcellular distribution of AAR2. Histone H3 and GAPDH were used as markers for nuclear and cytoplasmic fractions, respectively.

(**B**) HEK293T cells were transfected with Flag-tagged AAR2 and then fractionated into cytoplasmic (Cyto.) and nuclear (Nuc.) fractions, followed by immunoblotting to assess the subcellular distribution of AAR2.

(**C**) Subcellular localization of AAR2 in CACO-2 and U2OS cells. Data were obtained from the Human Protein Atlas (https://www.proteinatlas.org/). Green, AAR2 signal; blue, DAPI-stained nuclei.

(**D**) HEK293T cells infected with lentivirus encoding control shRNA (shCtrl) or NCDN-targeting shRNA (shNCDN) were transfected with Flag-AAR2. Cytoplasmic lysates were subjected to immunoprecipitation with anti-Flag antibodies and analyzed by immunoblotting with the indicated antibodies. Quantification of co-precipitated proteins is shown on the right.

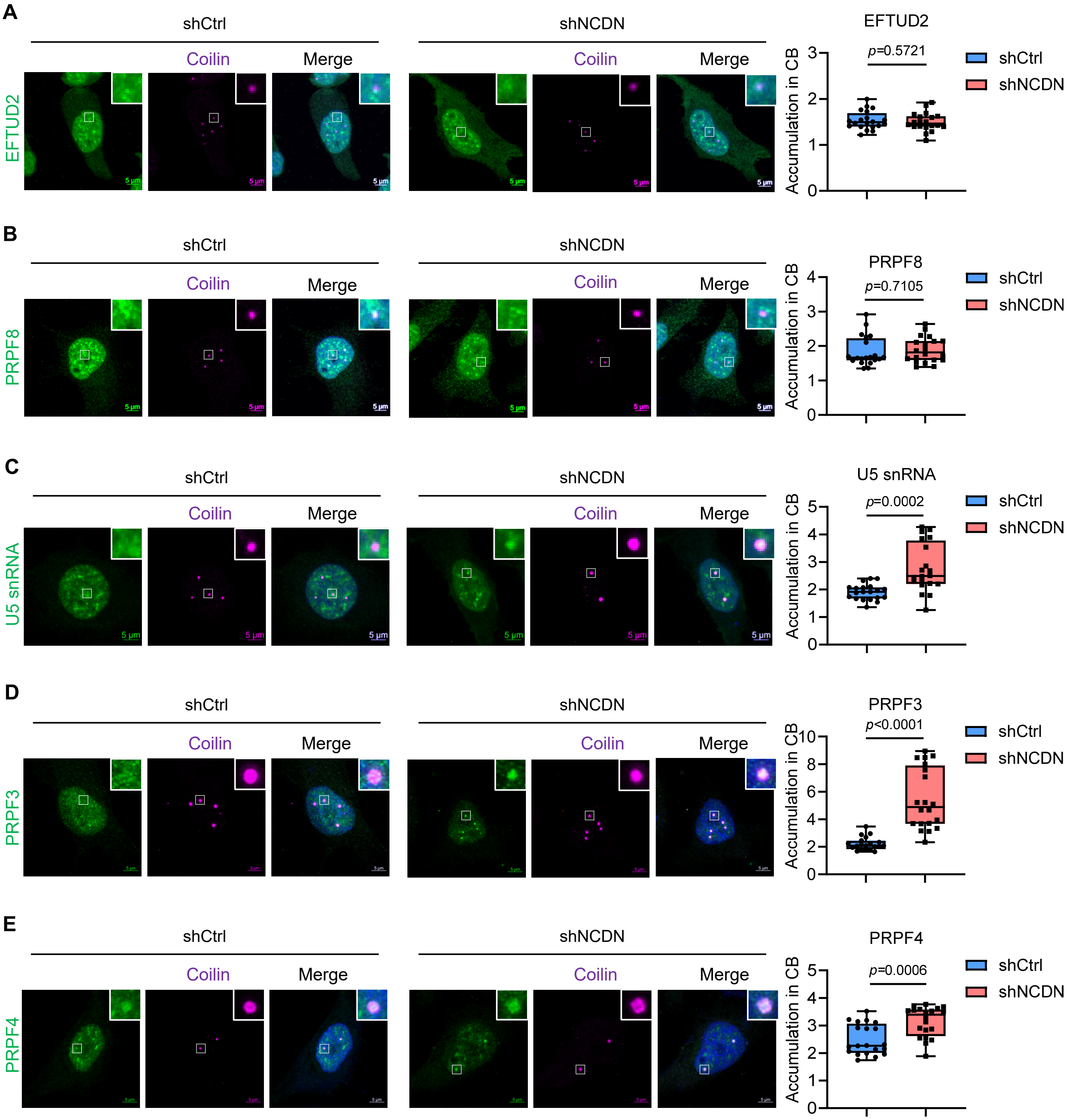


**Fig. S5 | Depletion of NCDN induces accumulation of U5 snRNA, PRPF3 and PRPF4 in Cajal bodies.** (**A−E**) Immunofluorescence analysis of Cajal bodies (anti-Coilin, magenta; red/green fluorescence was recolored to magenta/green for color-blind accessibility) alongside staining for EFTUD2 **(A)**, PRPF8 **(B)**, U5 snRNA **(C)**, PRPF3 **(D)** and PRPF4 **(E)** (green) in control and NCDN-depleted HeLa cells following lentiviral shRNA-mediated knockdown for 3 days. Nuclei were counterstained with DAPI (blue). Images show individual fluorescence channels, merged channels, and enlarged views of the boxed regions. Scale bars, 5 μm. Quantification of Cajal body accumulation is shown in right. The fluorescence signal of indicated protein or snRNA in Cajal bodies was normalized to signal in the nucleoplasm. Each dot represents one cell, and 20 cells per condition (control and NCDN knockdown) were analyzed. Data were obtained in two biologically independent experiments. *P* values were determined by unpaired two-tailed Student's *t*-test.

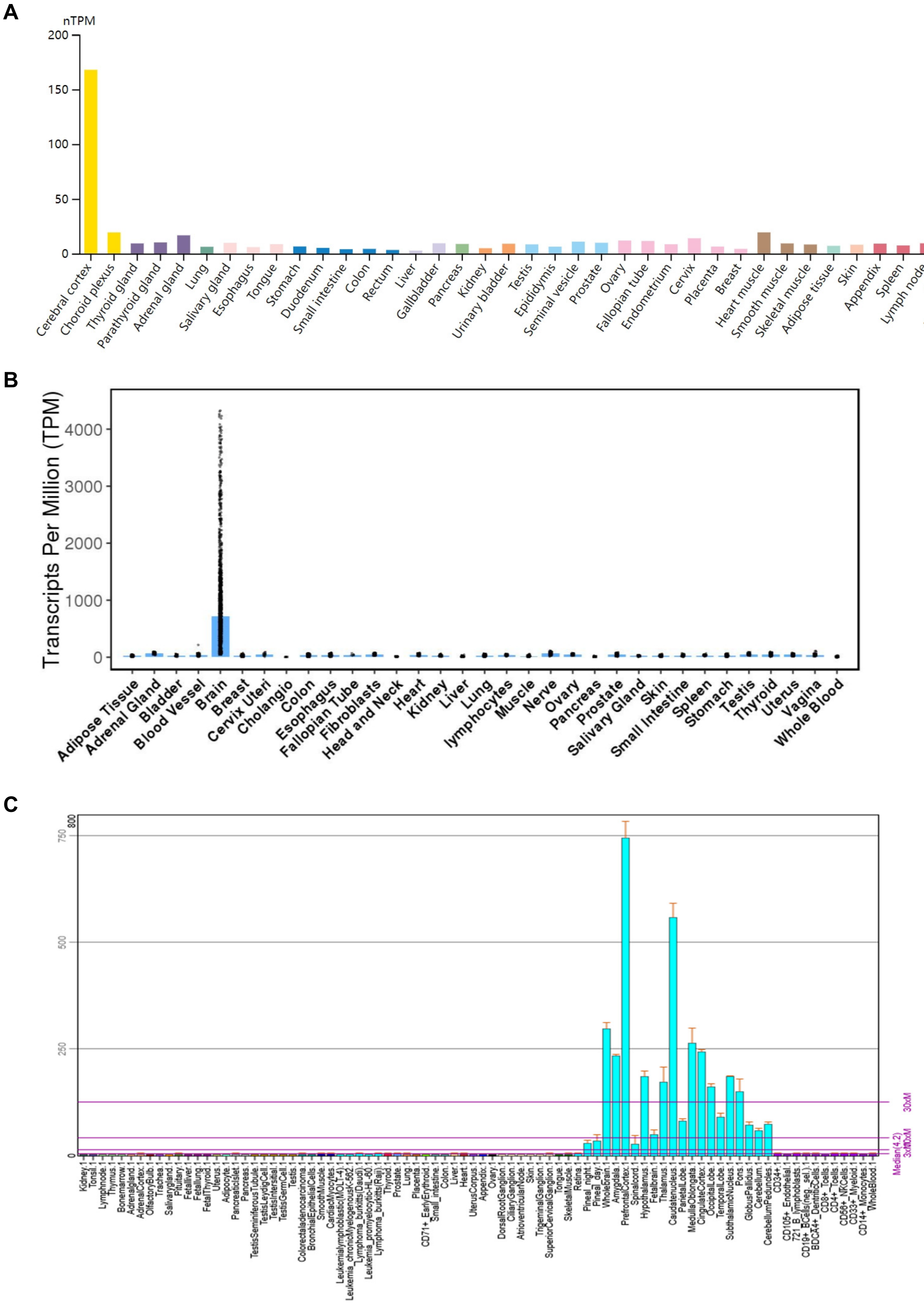


**Fig. S6 | NCDN expression profile.**

(**A**) NCDN transcript levels in various tissues from the Human Protein Atlas (HPA) dataset. The RNA-seq tissue data are reported as nTPM (normalized protein-coding transcripts per million). Color-coding is based on tissue groups, each consisting of tissues with functional features in common.

(**B**) NCDN transcript levels in various tissues. Data are from the website (http://gemini.cancer-pku.cn/).

(**C**) NCDN transcript levels in various tissues. Data are from the website (http://biogps.org/).

**Table S1.** Oligonucleotides used for RT-qPCR

| RT-qPCR primers | Sequence 5'–3' | RT-qPCR primers | Sequence 5'–3' |
|---|---|---|---|
| U4-snRNA-F | GCCAATGAGGTCTATCCGAGG | LPAR6-F | CCCACTGCTTCTATAATGACTCC |
| U4-snRNA-R | TCAAAAATTGCCAGTGCCG | LPAR6-R | GGACTTTGAGGACGCAGATG |
| U5-snRNA-F | TGGTTTCTCTTCAGATCGCATAAA | GADD45A-F | GGAGAGCAGAAGACCGAAAG |
| U5-snRNA-R | CCAAGGCAAGGCTCAAAAAAT | GADD45A-R | AGGCACAACACCACGTTATC |
| U6-snRNA-F | CTCGCTTCGGCAGCACA | PDGFA-F | GATACCTCGCCCATGTTCTG |
| U6-snRNA-R | AACGCTTCACGAATTTGCGT | PDGFA-R | CAAAGAATCCTCACTCCCTACG |
| 18S-F | CGGCTACCACATCCAAGGAA | NFKB2-F | GAAGCCAGTCATCTCCCAG |
| 18S-R | GCTGGAATTACCGCGGCT | NFKB2-R | CATCTTTCTGCACCTTGTCAC |
| CDK6-F | TCGATGAACTAGGCAAAGACC | FZD4-F | TTTCACACCGCTCATCCAG |
| CDK6-R | AGGTGGGAATCCAGGTTTTC | FZD4-R | TGACTGAAAGACACATGCCG |
| BCL2-F | GTGGATGACTGAGTACCTGAAC | ICAM-F | CAATGTGCTATTCAAACTGCCC |
| BCL2-R | GCCAGGAGAAATCAAACAGAGG | ICAM-R | CAGCGTAGGGTAAGGTTCTTG |
| KRAS-F | GGAGTACAGTGCAATGAGGG | ITGA5-F | ATACTCTGTGGCTGTTGGTG |
| KRAS-R | CCATAGGTACATCTTCAGAGTCC | ITGA5-R | CTGTTCCCCTGAGAAGTTGTAG |
| LAMC1-F | GACCTCTATCAAGATACGTGGG | ALCAM-F | TGGCAATATCACATGGTACAGG |
| LAMC1-R | AAAACTGCCCTCCATATCCC | ALCAM-R | AGCCTTGGTTGTCTTGTACTC |
| ETS1-F | TCCAGTCCAATTATCACCAGC | FAS-F | AAGCTCTTTCACTTCGGAGG |
| ETS1-R | TGCTTGGAGTTAATAGTGGGAC | FAS-R | GGGCATTAACACTTTTGGACG |
| RASSF1-F | ACACCTGACCTTTCTCAAGC | | |
| RASSF1-R | TGAAGCCTGTGTAAGAACCG | | |
| ACTB-F | ACCTTCTACAATGAGCTGCG | | |
| ACTB-R | CCTGGATAGCAACGTACATGG | | |

**Table S2.** Oligonucleotides used for RT-PCR

| RT-PCR primers | Sequence 5'–3' |
|---|---|
| BBC3-SE-Exon2-F | GTCTGCCCAGGCATGTCCATGCC |
| BBC3-SE-Exon2-R | TTCCTCCTCCCCGCGGACTCCC |
| MCL1-SE-Exon2-F | TACCGGCAGTCGCTGGAGATTATC |
| MCL1-SE-Exon2-R | ACCTGCAAAAGCCAGCAGCACA |
| CADM1-SE-Exon10-F | AACGGCGACGACAGAACCAG |
| CADM1-SE-Exon10-R | TTATGTCTGGCAAAATAGCGCCC |
| CD44-SE-Exon11-F | GGAAGAAACAGCTACCCAGA |
| CD44-SE-Exon11-R | CATTGAAAGAGGTCCTGTCC |
